# Supplementary material for: ALS mutations disrupt self-association between the ubiquilin STI1 hydrophobic groove and internal placeholder sequences
Source: EMBO J. 2026 Mar 20;45(8):2694–711. doi: 10.1038/s44318-026-00745-9 (PMC13083928; doi:10.1038/s44318-026-00745-9)
Supplement: Supplementary file 1 — Appendix [file 44318_2026_745_MOESM1_ESM.pdf]

Apppendix for Onwunma et al, ALS mutations disrupt self-association between the Ubiquilin STI1 hydrophobic groove and internal placeholder sequences

Table of Contents

Appendix Figure S1 2
Appendix Figure S2 3
Appendix Figure S3 4
Appendix Figure S4 5
Appendix Figure S5 6
Appendix Figure S6 7
Appendix Figure S7 8
Appendix Figure S8 9
Appendix Figure S9 10
Appendix Figure S10 11

Appendix Figure S1

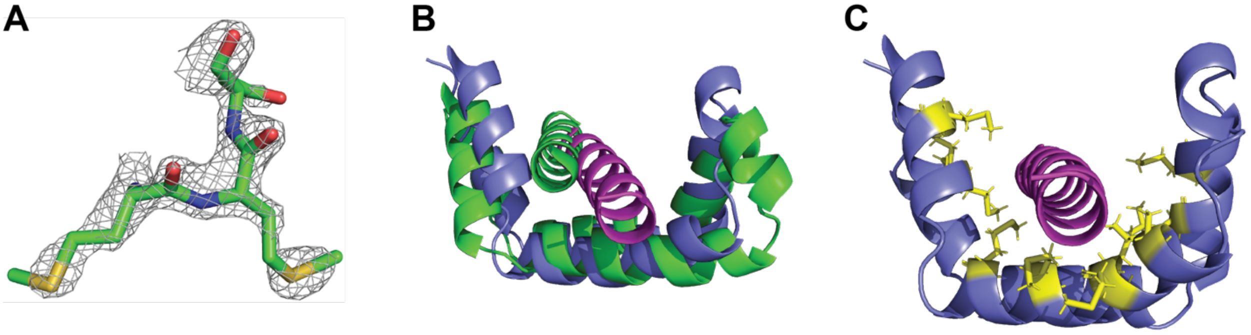

Appendix Figure S1: Crystal structure of the Dsk2 STI1 domain bound to a TMD

A) Fitting of the final refined model for residues 68-70 in the σA-weighted 2*F*o-*F*c electron density map contoured at 3 sigma.

B) Comparison of the crystal structure (slate and magenta) with the AlphaFold 3 prediction of a crystallization construct dimer (green). For clarity, only one STI1 domain and TMD are shown.

C) STI1 residues that contact the TMD are shown as yellow sticks. These include residues 30, 33, 47, 50, 51, 54, 58, 65, 68, and 69 in the crystallization construct.

Appendix Figure S2

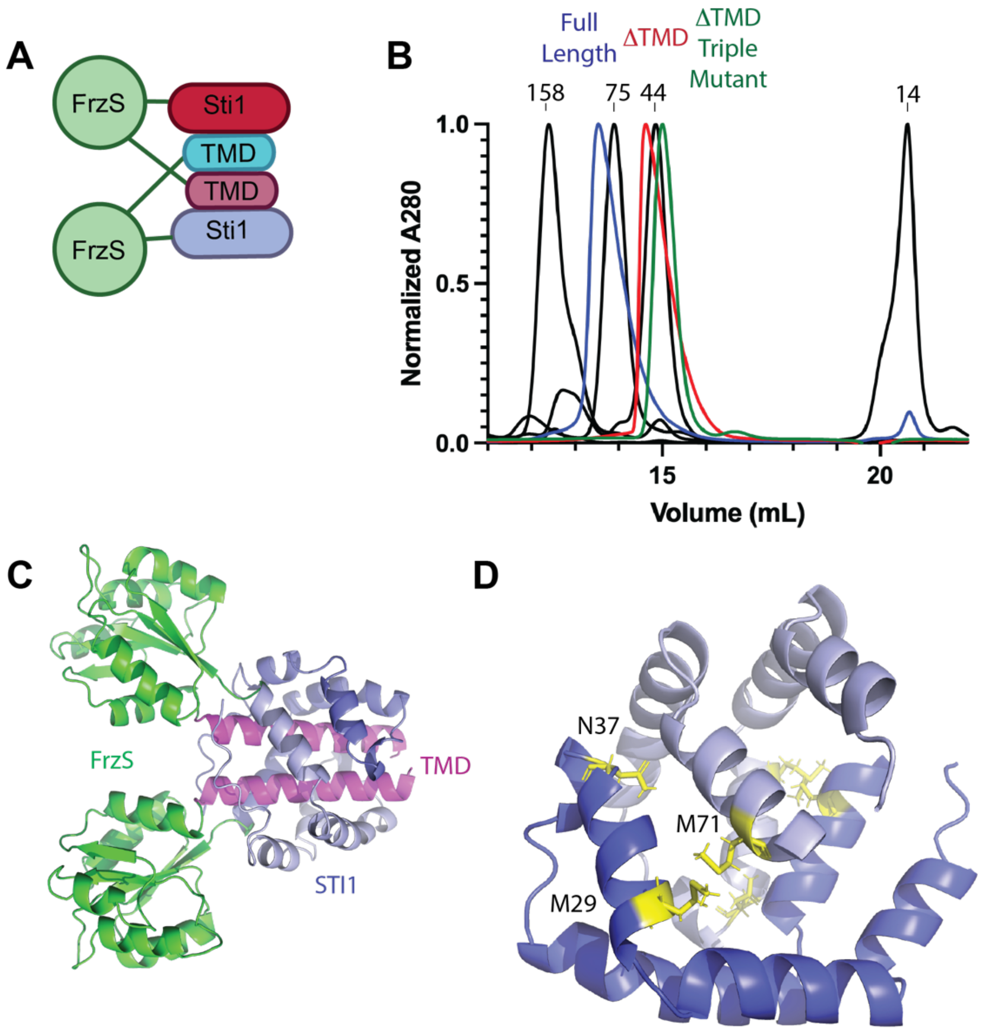

Appendix Figure S2: The asymmetric unit contains a domain-swapped dimer.

A) Diagram showing the domain swap in the crystal structure.
B) Size exclusion chromatography of crystallization construct variants. The crystallization construct STI1-FrzS-TMD is shown in blue and has a predicted molecular weight of 24.4 kDa. The STI1-FrzS-ΔTMD and STI1-FrzS-ΔTMD M29D, N37A, M71D triple mutant have a predicted molecular weight of 22 kDa and are shown in red and green, respectively. Standards of Aldolase, Conalbumin, Ovalbumin, and Lysozyme are shown in black with molecular weight noted on the chart.
C) Structure of the STI1-FrzS-TMD domain swapped dimer observed in the asymmetric unit. STI1 domain is slate, FrzS domain is green, and TMD is magenta. Note that there is no contact between the two FrzS domains.
D) Residues M29, N37, and M71, shown as yellow sticks, mediate contacts between the two STI1 domains in the crystal structure.

Appendix Figure S3

A

```
                      P189T
MB Dsk2 165 EQLEGIMLSPAFQEQMNALMLDPRMLDMMIDQNPQLRNMG  204
UBQLN1  184 EMMVQIMENPFVQ----SMLSNPDLMRQLIMANPQMQQLI  219
UBQLN2  180 EMMIQIMENPFVQ----SMLSNPDLMRQLIMANPQMQQLI  215
UBQLN4  194 EMLSQIMENPLVQ----DMMSNPDLMRHMIMANPQMQQLM  229

MB Dsk2 205 PEARQMLRPMFREMMSNPESMRSMMNMGRA  234
UBQLN1  220 Q-----RNPEISHMLNNPDIMRQTLELARN  244
UBQLN2  216 Q-----RNPEISHLLNNPDIMRQTLEIARN  240
UBQLN4  230 E-----RNPEISHMLNNPELMRQTMELARN  254
```

B

```
MB Dsk2 165 EQLEGIMLSPAFQEQMNALMLDPRMLDMMI------DQNPQLRN  202
UBQLN1  389 QLMQNMLSAPYMRSMMQSLSQNPDLAAQMMLNNPLFAGNPQLQE  432
UBQLN2  381 QLIQNMLSAPYMRSMMQSLSQNPDLAAQMMLNSPLFTANPQLQE  424
UBQLN4  395 QLMQNVISAPYMRSMMQTLAQNPDFAAQMMVNVPLFAGNPQLQE  438

                Q425R           P440L M446R
MB Dsk2 203 MGPEARQMLRPMFREMMSNPESMRSM---------MNMGRA-  234
UBQLN1  433 ----QMRQQLPTFLQQMQNPDTLSAMSNPRAMQALLQIQQGL  470
UBQLN2  425 ----QMRPQLPAFLQQMQNPDTLSAMSNPRAMQALMQIQQGL  462
UBQLN4  439 ----QLRLQLPVFLQQMQNPESLSILTNPRAMQALLQIQQGL  476
```

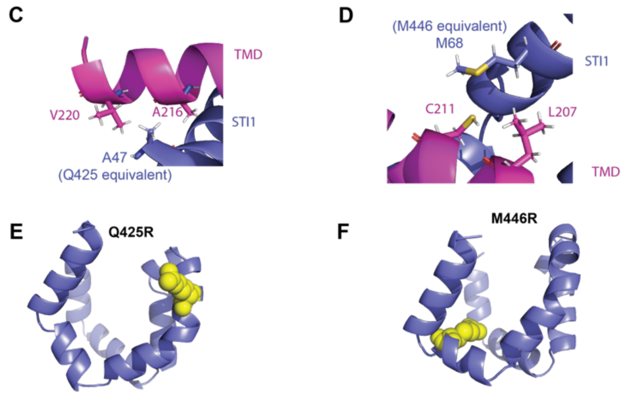

Appendix Figure S3: Sequence alignment of *M. bicuspidata* Dsk2 with UBQLN2

A) Sequence alignment between *M. bicuspidata* Dsk2 and the Ubiquilin STI1-I domains. Alignments were performed in Clustal Omega using the annotated domains from UBQLN1, UBQLN2, & UBQLN4. ALS causing mutations in UBQLN2 are highlighted in bold. This alignment was used to map UBQLN2 ALS mutations onto the Dsk2 structure for analysis in Figure 2.

B) Sequence alignment of *M. bicuspidata* Dsk2 and Ubiquilin STI1-II domains. Alignments performed as in A.

C) Analysis of the crystal structure shows that A47 from the STI1 domain forms hydrophobic contacts with A216 and V220 in the TMD. A47 in the crystal construct aligns with Q425 in UBQLN2.

D) Analysis of the crystal structure shows that M68 from the STI1 domain forms hydrophobic contacts with L207 and C211 in the TMD. M68 in the crystal construct aligns with M446 in UBQLN2.

E) AlphaFold 3 model of the human UBQLN2 STI1-II domain shows that the Q425R mutation is predicted to disrupt the hydrophobic groove via insertion of a charged residue.

F) AlphaFold 3 model of the human UBQLN2 STI1-II domain shows that the M446R mutation is predicted to disrupt the hydrophobic groove via insertion of a charged residue.

Appendix Figure S4

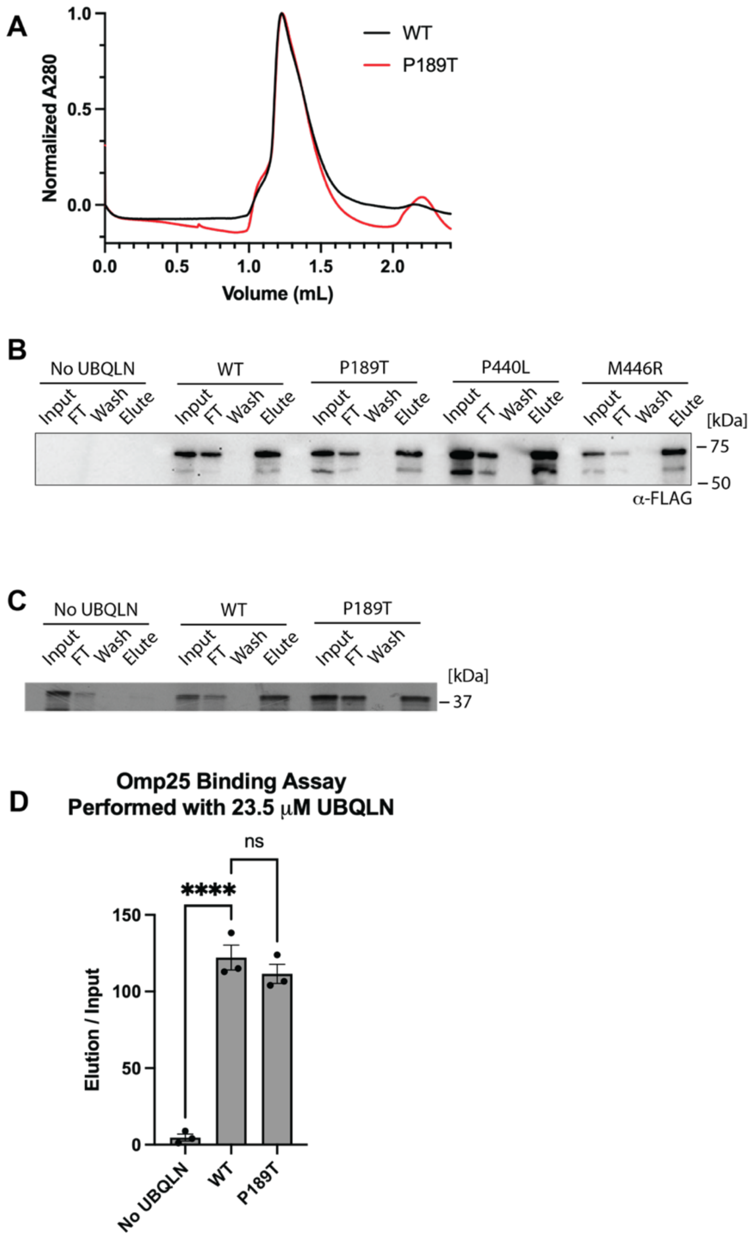

Appendix Figure S4: The STI1-I domain has higher affinity for substrate binding than the STI1-II domain.

A) Size exclusion chromatography shows that P189T UBQLN2 behaves identically to WT UBQLN2.
B) Anti-Flag western blot shows that UBQLN2 mutants have no defect in immunoprecipitation efficiency. Omp25 binding assay was performed as in Figure 2F, except sample was analyzed by anti-FLAG western blot.
C) Using a higher concentration of UBQLN2 allows P189T to bind to Omp25. Binding assay was performed as in Figure 2F, except 23.5 μM UBQLN2 was used instead of 3 μM.
D) Quantification of data in C, error bars are standard deviation, $N \geq 3$. P-values were calculated by 1-way ANOVA with Dunnett's post hoc test; **** $P < 0.0001$, ns: not significant.

Appendix Figure S5

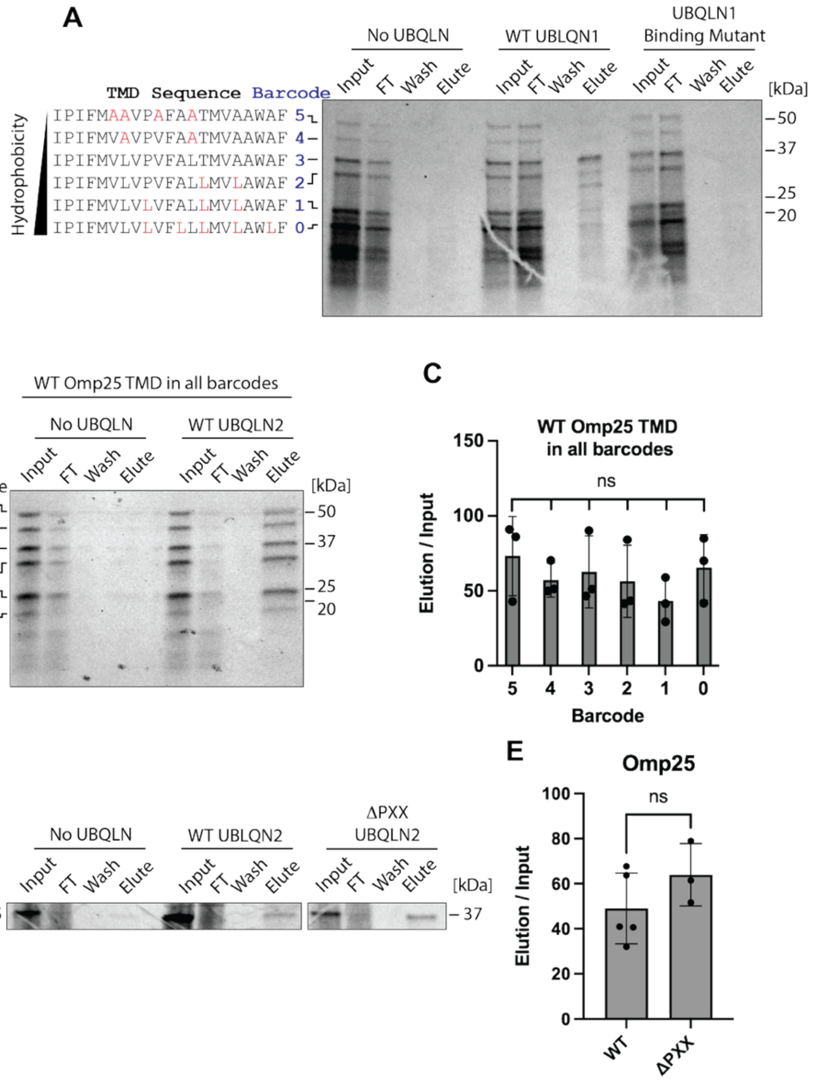

Appendix Figure S5: The barcoded binding assay is driven by the interaction of the TMD with Ubiquilin STI1 domains

A) Substrate binding depends on interaction with the STI1 domains. Binding assay with the Omp25 substrate series with no Ubiquilin control, WT UBQLN1, and the UBQLN1 binding mutant. The binding mutant contains two mutations in each STI1 domain that are predicted to disrupt the hydrophobic groove (M186D, M228D, M394D, M454D).
B) Barcoded binding assay with the WT Omp25 sequence cloned into all six barcodes shows no significant difference in the amount of substrate bound.
C) Quantification of the data in B, error bars are standard deviation. P-values were calculated by 1-way ANOVA with Dunnett's post hoc test; ns: not significant.
D) WT and ΔPXX UBQLN2 bind to Omp25 with equal affinity. Images cropped from the same film.
E) Quantification of the data in D, error bars are standard deviation. P-values were calculated by unpaired two-tailed T-test; ns: not significant.

Appendix Figure S6

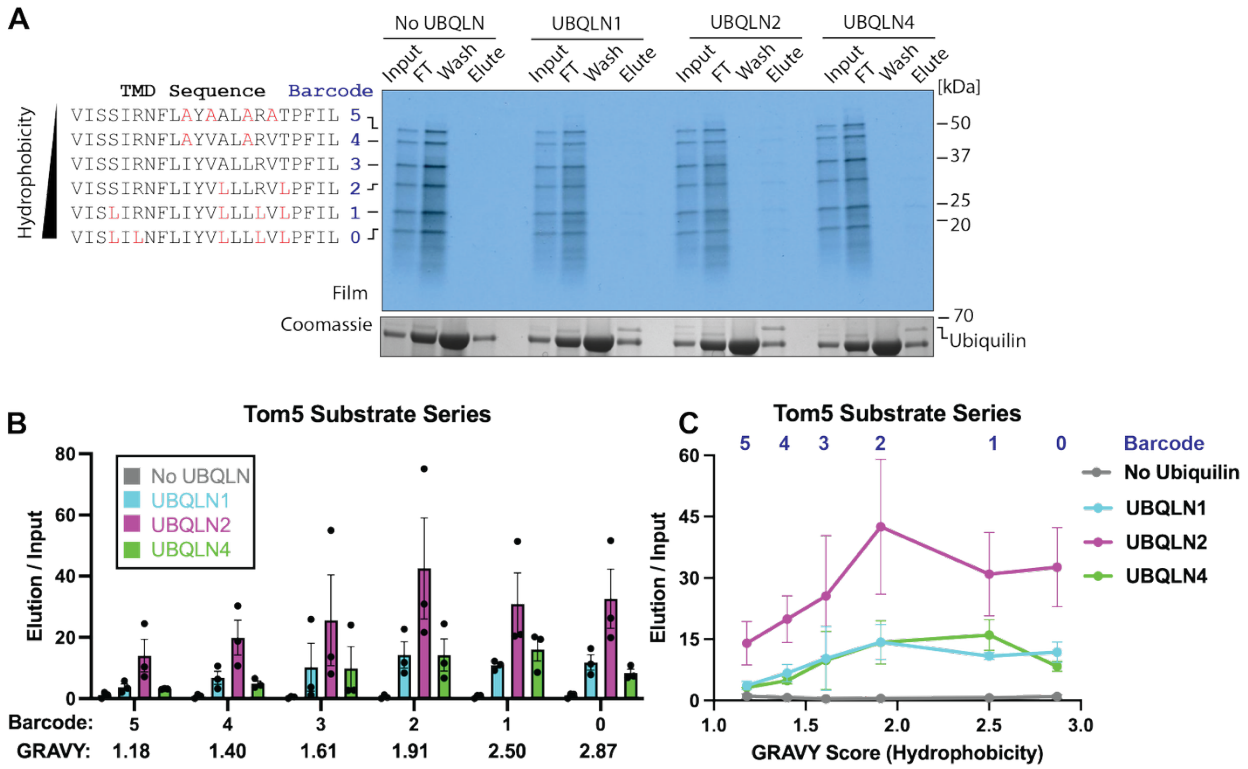

Appendix Figure S6: Barcoded binding data with the Tom5 substrate series

A) Barcoded binding assay with the Tom5 substrate series. The wild type Tom5 TMD has a barcode of 3.

B) Quantification of the data in A, organized by barcode. Error bars are standard error of the mean. A value of 100 corresponds to equal intensity of input and elution bands.

C) Quantification of the data in A, organized by TMD hydrophobicity (GRAVY score). A higher GRAVY score indicates a more hydrophobic TMD. Barcode is noted at the top of the graph. Error bars are standard error of the mean.

Appendix Figure S7

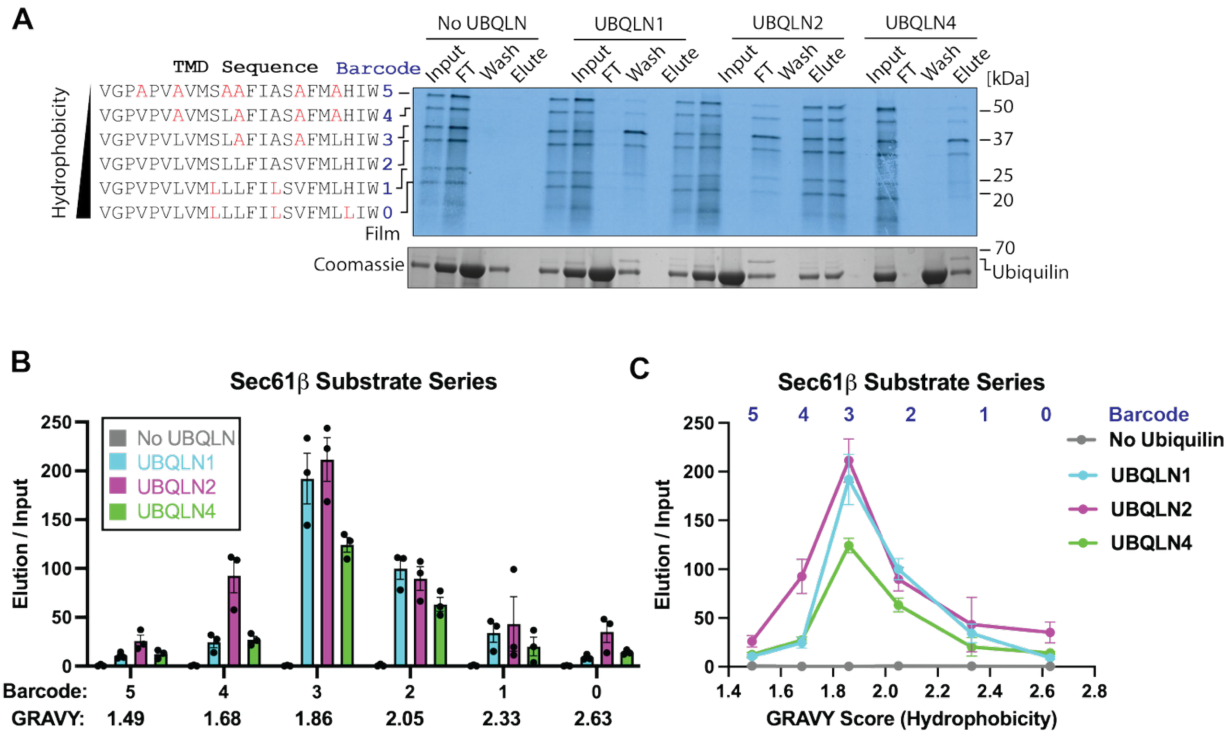

Appendix Figure S7: Barcoded binding data with the Sec61β substrate series

A) Barcoded binding assay with the Sec61β substrate series. The wild type Sec61β TMD has a barcode of 2. Note that the elution fraction is 5x more concentrated than the input fraction.

B) Quantification of the data in A, organized by barcode. Error bars are standard error of the mean. A value of 100 corresponds to equal intensity of input and elution bands.

C) Quantification of the data in A, organized by TMD hydrophobicity (GRAVY score). A higher GRAVY score indicates a more hydrophobic TMD. Barcode is noted at the top of the graph. Error bars are standard error of the mean.

Appendix Figure S8

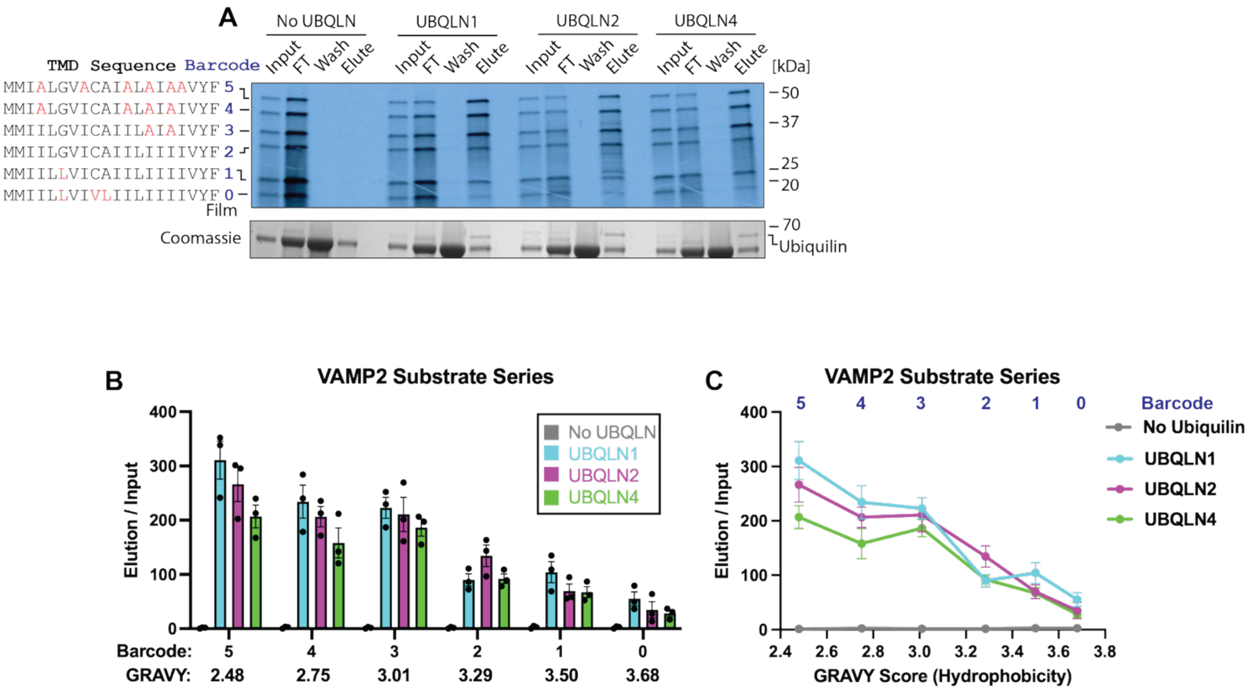

Appendix Figure S8: Barcoded binding data with the VAMP2 substrate series

A) Barcoded binding assay with the VAMP2 substrate series. The wild type VAMP2 TMD has a barcode of 2.
B) Quantification of the data in A, organized by barcode. Error bars are standard error of the mean. A value of 100 corresponds to equal intensity of input and elution bands.
C) Quantification of the data in A, organized by TMD hydrophobicity (GRAVY score). A higher GRAVY score indicates a more hydrophobic TMD. Barcode is noted at the top of the graph. Error bars are standard error of the mean.

Appendix Figure S9

```
Dsk2    ------------------------------MSLNIHIKSGQDKWEVNVAPESTVLQFKEA 30
Ubqln2  MAENGESSGPPRPSRGPAAAQGSAAAPAEPKIIKVTVKTPKEKEEFAVPENSSVQQFKEA 60
                                      ::: :*: ::* *. *   :*:* *****
          UBL
Dsk2    INKANGIPVANQRLIYSGKILKDDQTVESYHIQDGHSVHLVKSQPKPQTGSAAEANNATA 90
Ubqln2  ISKRFKSQTDQLVLIFAGKILKDQDTLIQHGIHDGLTVHLVIKSQNRPQGQSTQPSNAAG 120
        *.*       . :   **::******::*: .: *:** :**** .. :    *.::: .**:.
                                    Placeholder 1
Dsk2    TGAAA----GTGATPNMSSGQSAGFNPLADLTSARYAGYLNMPSADMFGPDGGALNNDSN 146
Ubqln2  TNTTSASTPRSNSTPISTNSNPFGLGSLGGLAGLSSLGL---SSTNFSELQSQMQQQLMA 177
        *.:::      :.:**  :..:  *:. *..*:.     *      *:::    :.    ::
          Sti1
Dsk2    NQDELLRMMENPIFQSQMNEMLSNPQMLDFMIQSNPQLQAMGPQARQMLQSPMFRQMLTN 206
Ubqln2  SPEMMIQIMENPFVQ----SMLSNPDLMRQLIMANPQMQQLI------QRNPEISHLLNN 227
        . : ::::****:.*    .*****:::  :* :***:* :         :.* : ::*.*

Dsk2    PDMIRQSMQFARMMDPNAGMG-------------------------------------SA 229
Ubqln2  PDIMRQTLEIARNPAMMQEMMRNQDLALSNLESIPGGYNALRRMYTDIQEPMLNAAQEQF 287
        **::**::::**       *                                      .

Dsk2    GGA----------------------ASAFPAPGGDAPEEGSNTNTTSSSNTGNNAGTNAG 267
Ubqln2  GGNPFASVGSSSSSGEGTQPSRTENRDPLPNPWAPPPATQSSATTSTTTSTGSGSGNSSS 347
            **                       . :* *  .  *    *.:.*::::.**..:*..:.
                  Placeholder 2            Placeholder 3
Dsk2    TNAGANTAANPF-ASLLNPALNPFANAGNAASTGMPAFDPALLASMFQPPAQA------- 319
Ubqln2  NATGNTVAAANYVASI-------FSTPGMQSLLQQITENPQLIQNMLSAPYMRSMMQSLS 400
        . :* ..**  : **:        *:. *  :      : :* *: .*:. *

Dsk2    ------------------------------------------------------------ 319
Ubqln2  QNPDLAAQMMLNSPLFTANPQLQEQMRPQLPAFLQQMQNPDTLSAMSNPRAMQALMQIQQ 460


Dsk2    ------------------------------------------------------------ 319
Ubqln2  GLQTLATEAPGLIPSFTPGVGVGVLGTAIGPVGPVTPIGPIGPIVPFTPIGPIGPIGPTG 520


Dsk2    ------------------------------------------------SQAEDTRPPEE 330
Ubqln2  PAAPPGSTGSGGPTGPTVSSAAPSETTSPTSESGPNQQFIQQMVQALAGANAPQLPNPEV 580
                                                        ::* :    **
             UBA
Dsk2        RYEHQLRQLNDMGFFDFDRNVAALRRSGGSVQGALDSLLNGDV--   373
Ubqln2      RFQQQLEQLNAMGFLNREANLQALIATGGDINAAIERLLGSQPS*   624
            *:::**.*** ***:: : *: **  :**.::.*:: **..:
```

Appendix Figure S9: Sequence alignment shows moderate conservation of placeholder sequences in Dsk2 and UBQLN2.

Sequence alignment of *S. cerevisiae* Dsk2 with human UBQLN2. Dsk2 placeholders 1, 2, and 3, highlighted in cyan, magenta, and yellow, were identified by Acharya et al. as potential STI1 interacting motifs(Acharya *et al*, 2025). Putative UBQLN2 placeholder sequences were chosen based on a combination of sequence alignments, AlphaFold predictions, GRAVY, and Agadir scores.

Appendix Figure S10

A

Name	Residues	Sequence
PH3	377-387	TENPQLIQNML

B

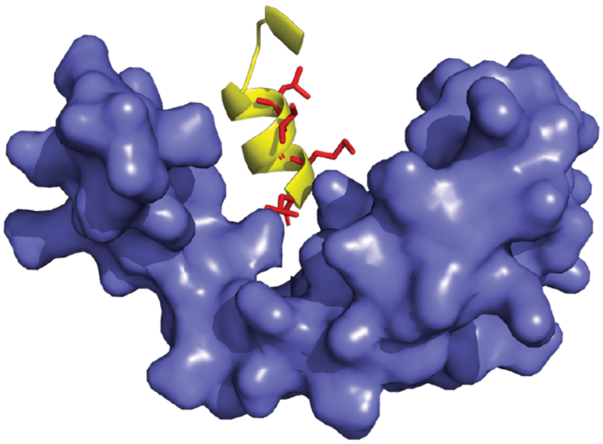

Appendix Figure S10: Placeholder 3 has a hydrophobic face and is positioned to interact with STI1

A) Placeholder 3 sequence with hydrophobic residues highlighted in red.
B) AlphaFold 3 model of UBQLN2 shows that PH3 forms an amphipathic helix with hydrophobic residues, shown in red, well positioned to interact with the hydrophobic groove of STI1-II surface.
