# Supplementary figures and images for: ALS mutations disrupt self-association between the ubiquilin STI1 hydrophobic groove and internal placeholder sequences
Source: EMBO J. 2026 Mar 20;45(8):2694–711. doi: 10.1038/s44318-026-00745-9 (PMC13083928; doi:10.1038/s44318-026-00745-9)

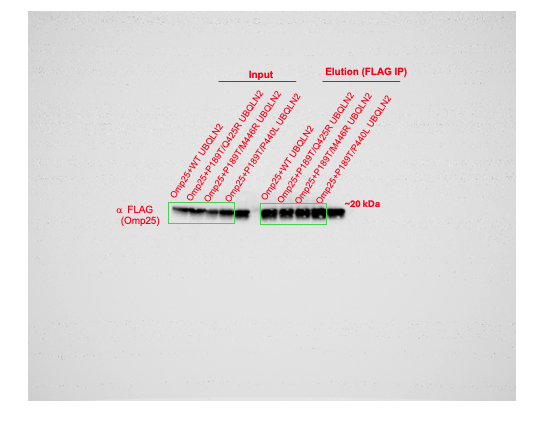

Supplement: Supplementary file 3 — Source data Fig. 2 [file 44318_2026_745_MOESM3_ESM.zip › Figure 2/2I/2I_FLAG_Input_Elution_Annotated.png]

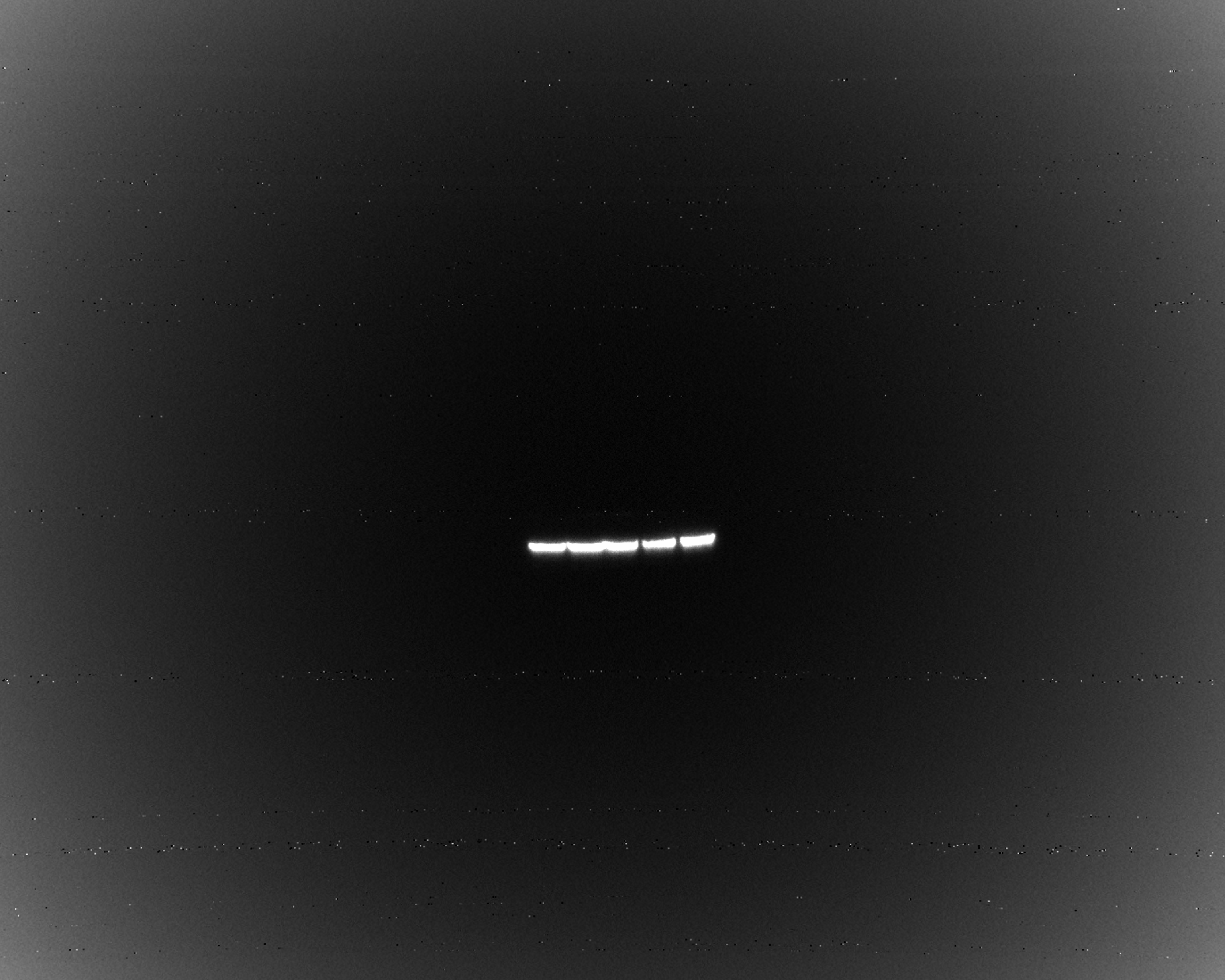

Supplement: Supplementary file 3 — Source data Fig. 2 [file 44318_2026_745_MOESM3_ESM.zip › Figure 2/2I/2I_Actin_Original.tif]

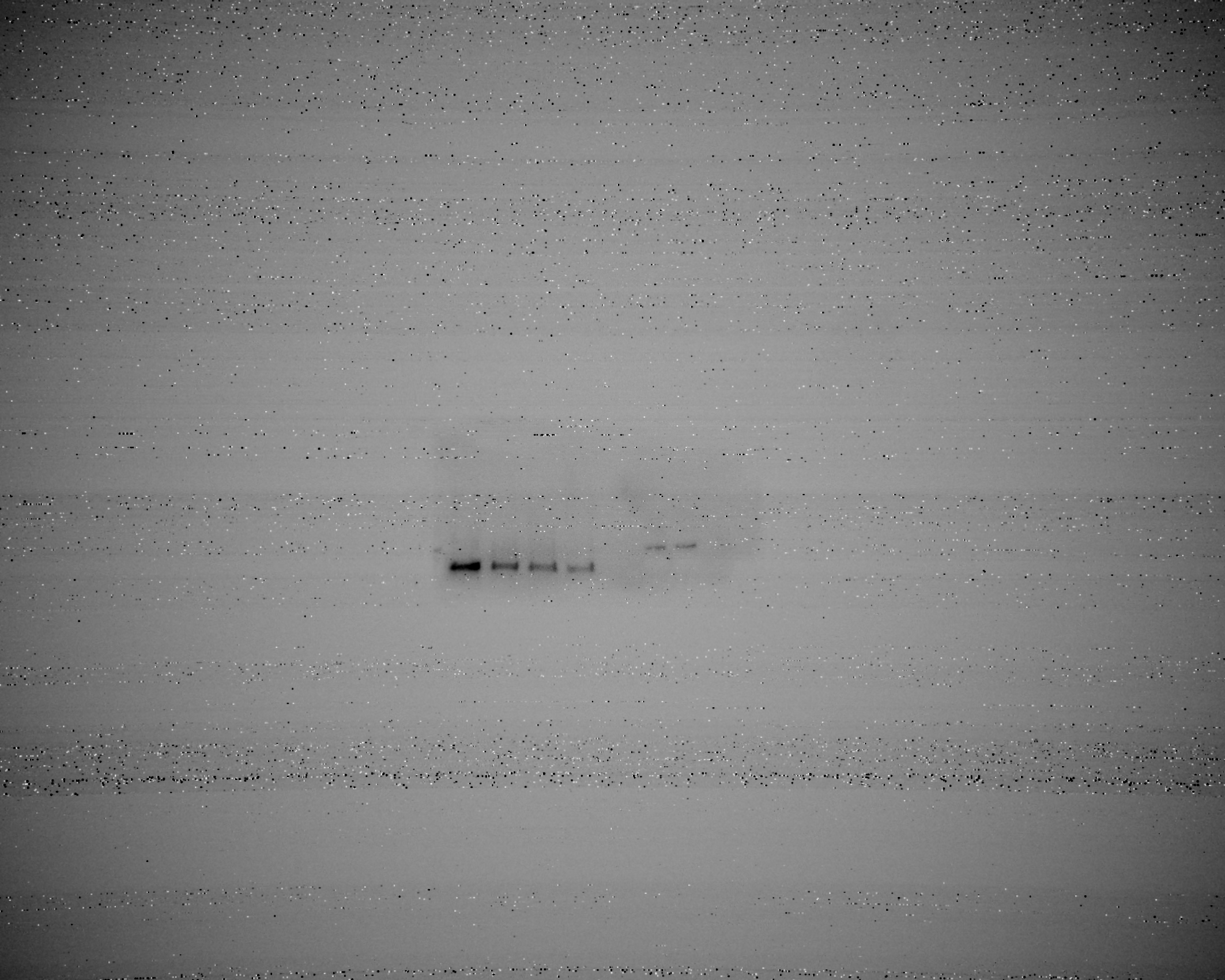

Supplement: Supplementary file 3 — Source data Fig. 2 [file 44318_2026_745_MOESM3_ESM.zip › Figure 2/2I/2I_HA_Elution_Original.tif]

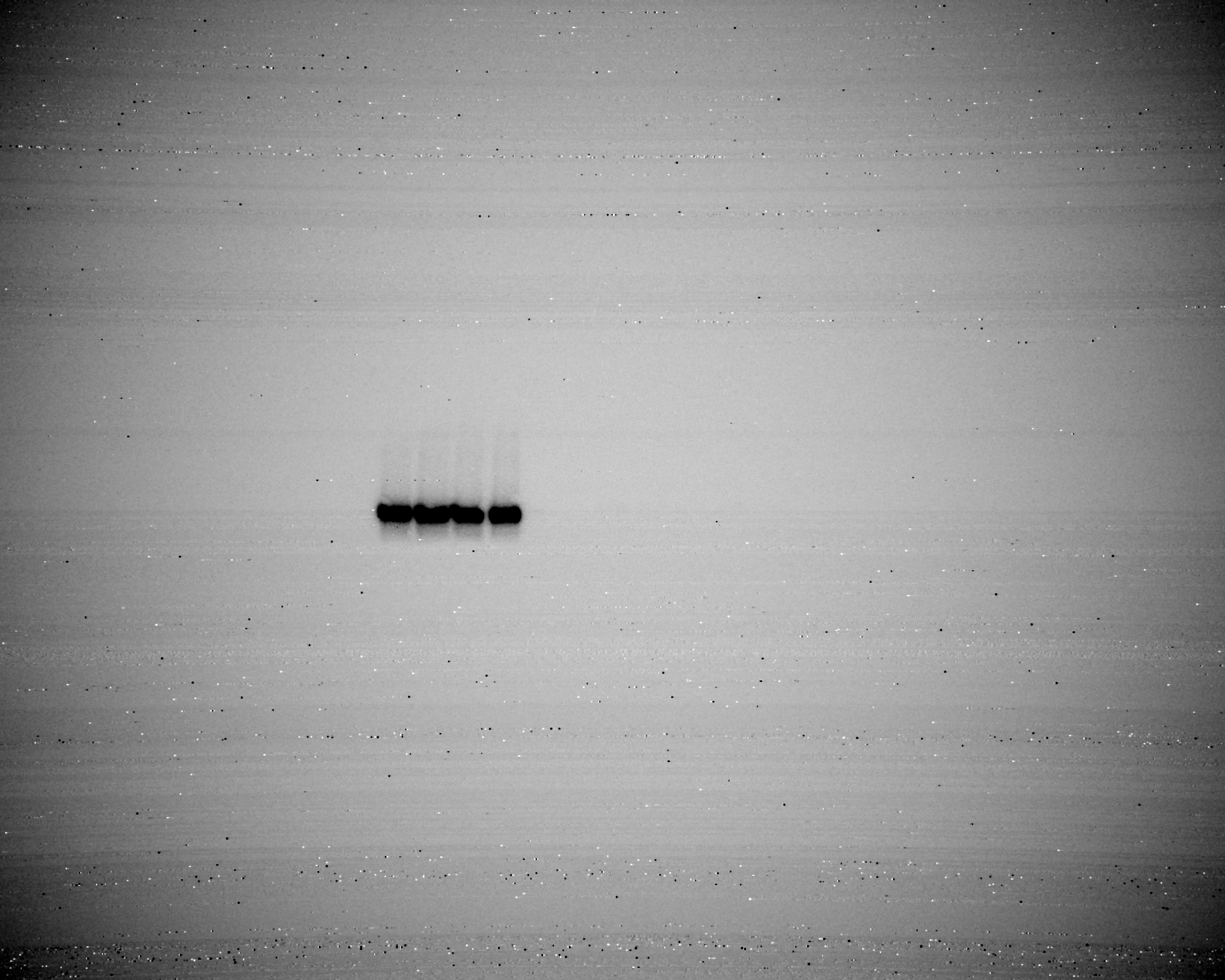

Supplement: Supplementary file 3 — Source data Fig. 2 [file 44318_2026_745_MOESM3_ESM.zip › Figure 2/2I/2I_HA_Input_Original.tif]

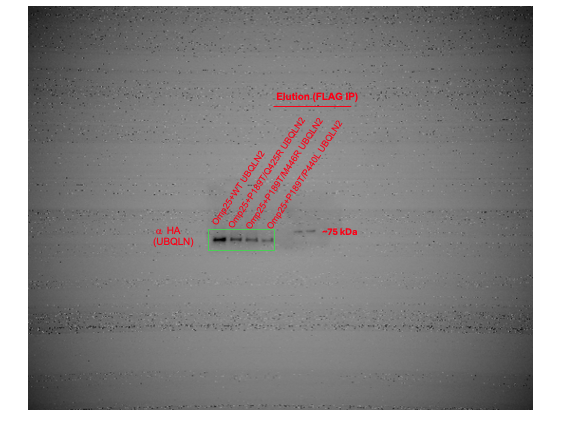

Supplement: Supplementary file 3 — Source data Fig. 2 [file 44318_2026_745_MOESM3_ESM.zip › Figure 2/2I/2I_HA_Elution_Annotated.png]

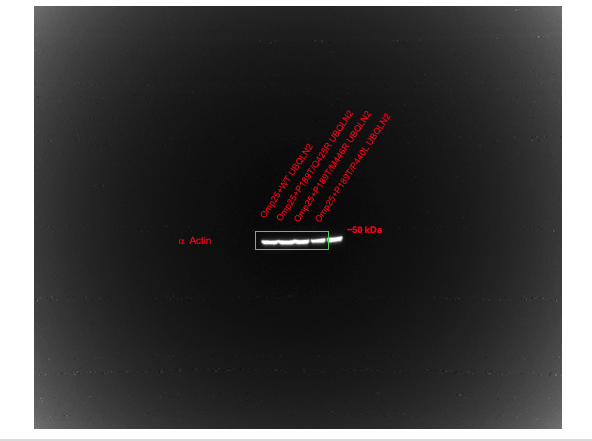

Supplement: Supplementary file 3 — Source data Fig. 2 [file 44318_2026_745_MOESM3_ESM.zip › Figure 2/2I/2I_Actin_Annotated.png]

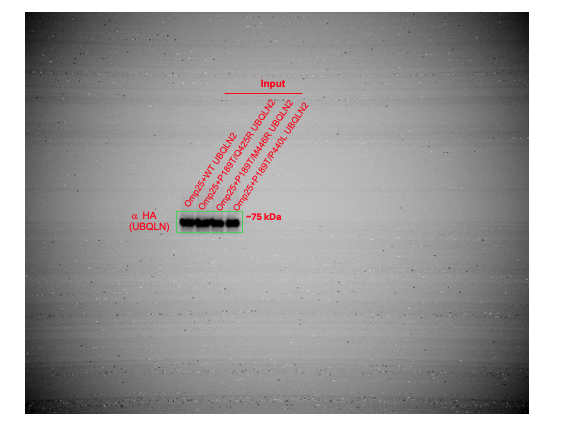

Supplement: Supplementary file 3 — Source data Fig. 2 [file 44318_2026_745_MOESM3_ESM.zip › Figure 2/2I/2I_HA_Input_Annotated.png]

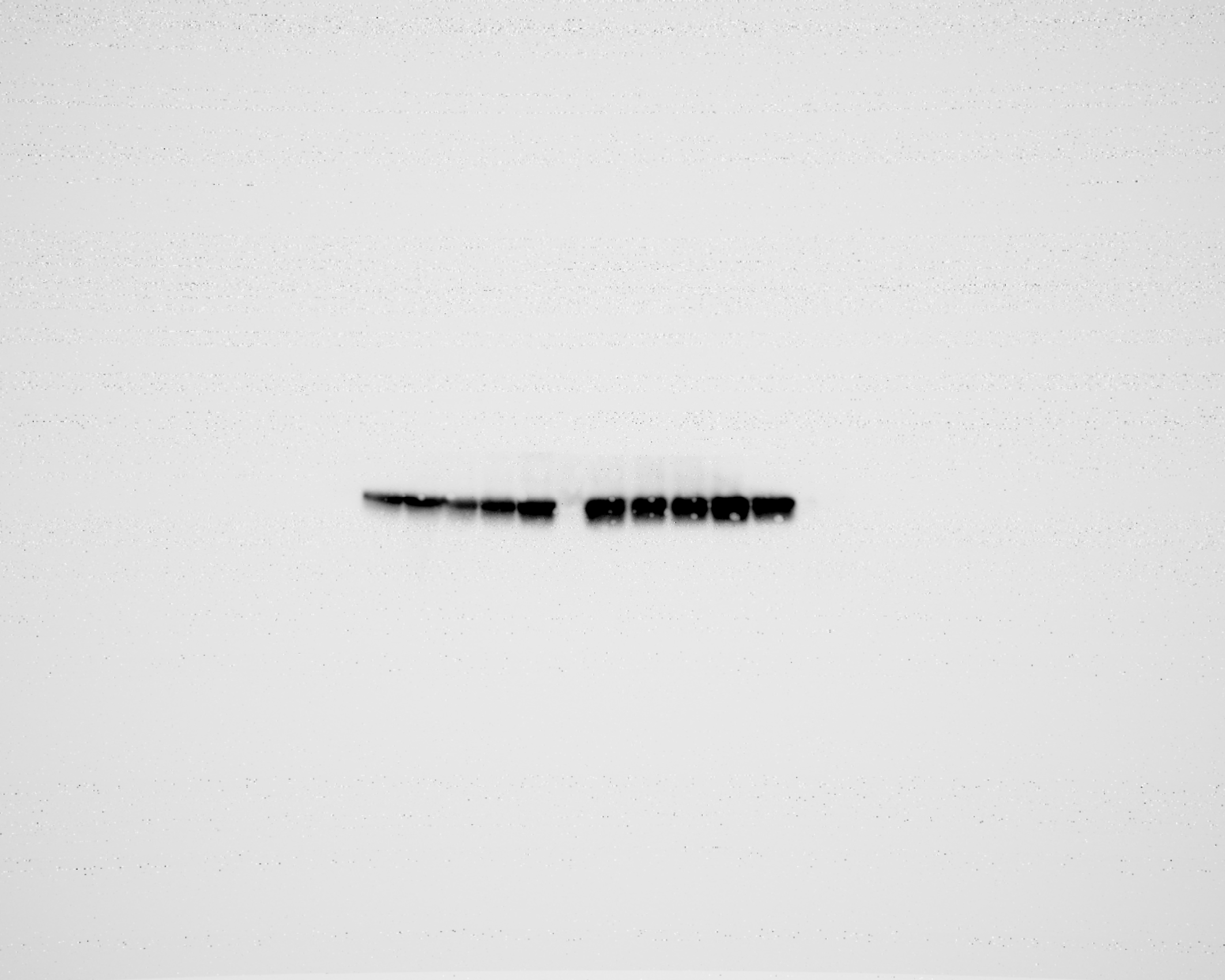

Supplement: Supplementary file 3 — Source data Fig. 2 [file 44318_2026_745_MOESM3_ESM.zip › Figure 2/2I/2I_FLAG_Input_Elution_Original.tif]

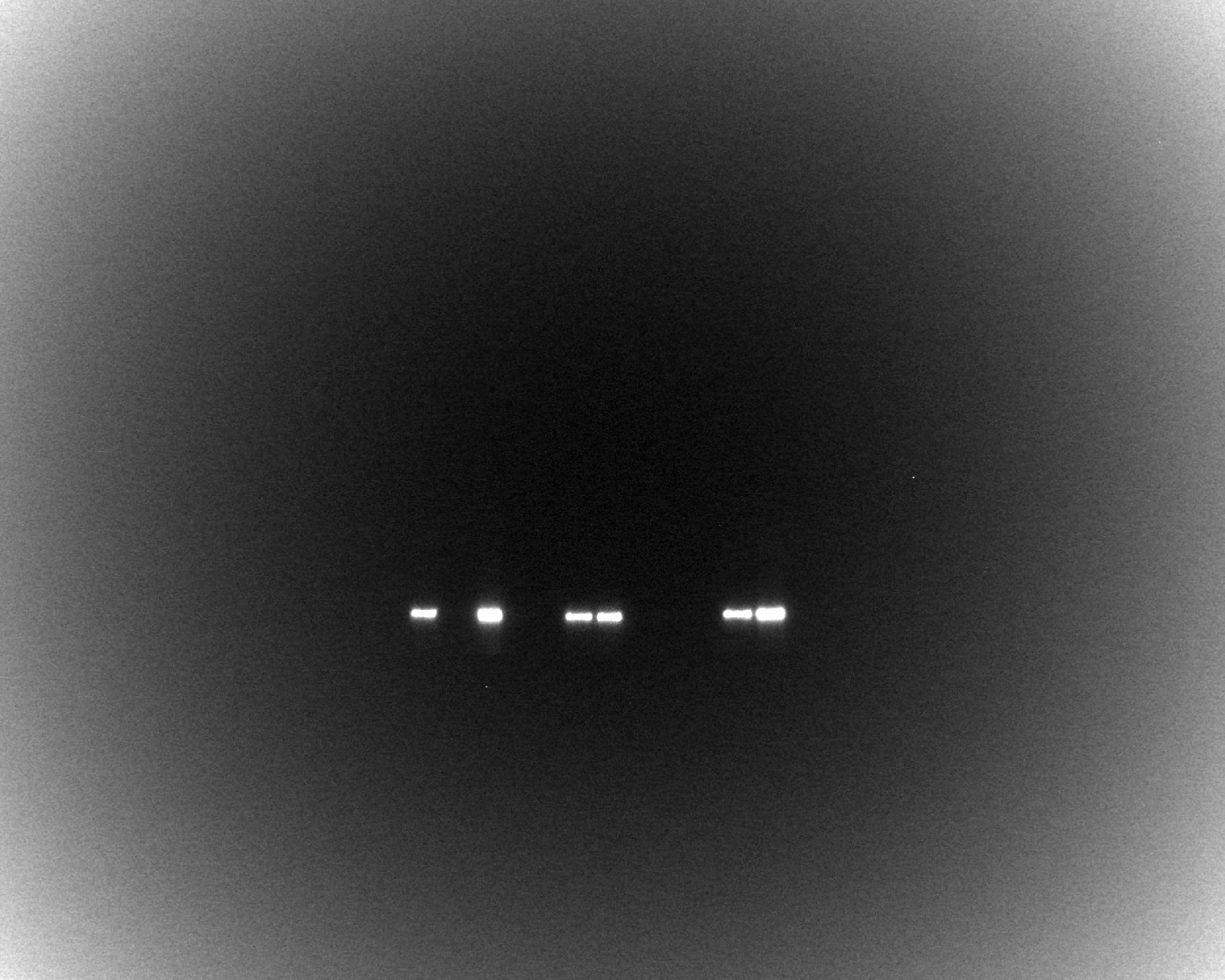

Supplement: Supplementary file 3 — Source data Fig. 2 [file 44318_2026_745_MOESM3_ESM.zip › Figure 2/2H/2H_HA_Input_Original.tif]

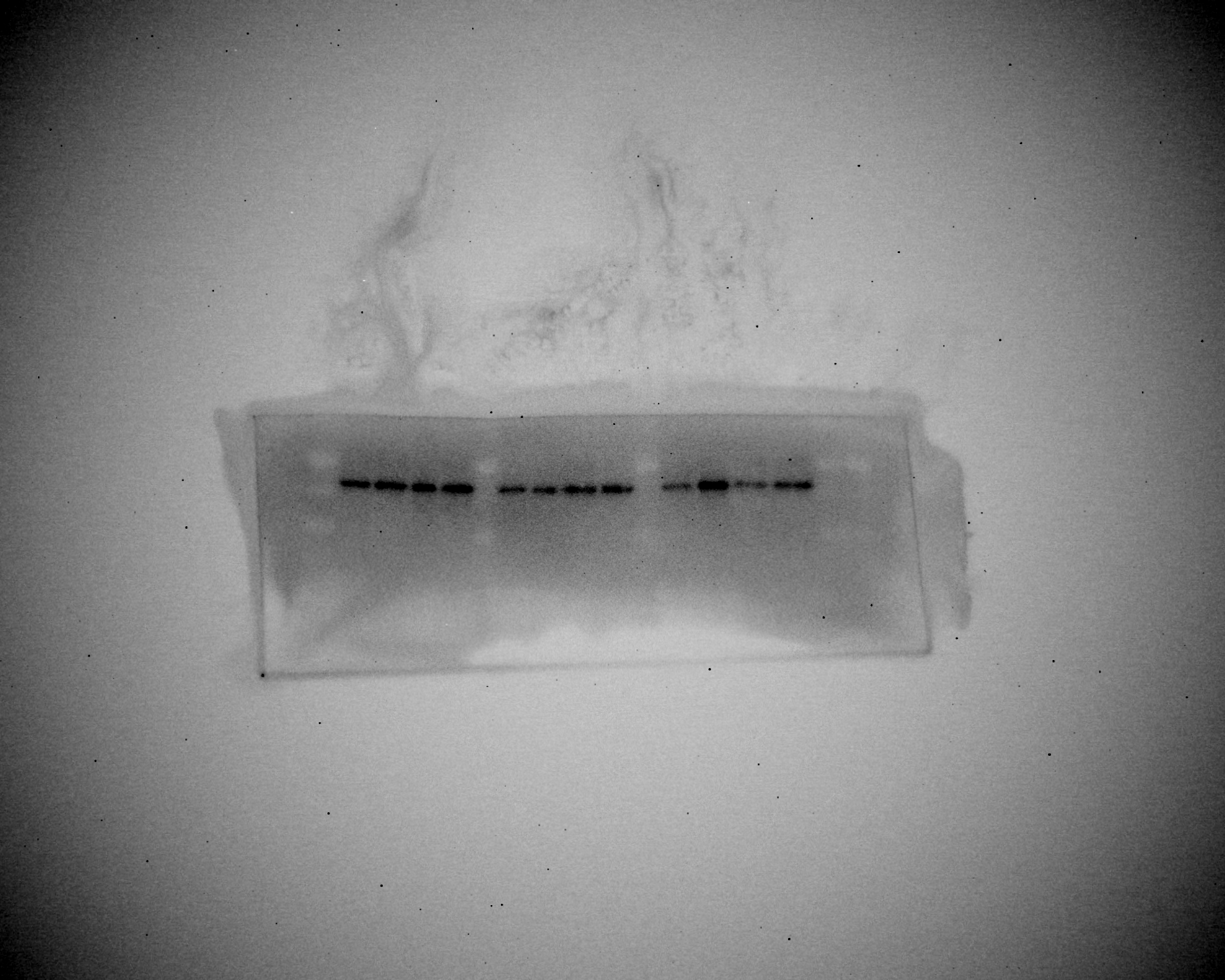

Supplement: Supplementary file 3 — Source data Fig. 2 [file 44318_2026_745_MOESM3_ESM.zip › Figure 2/2H/2H_FLAG_Input_Elution_Original.tif]

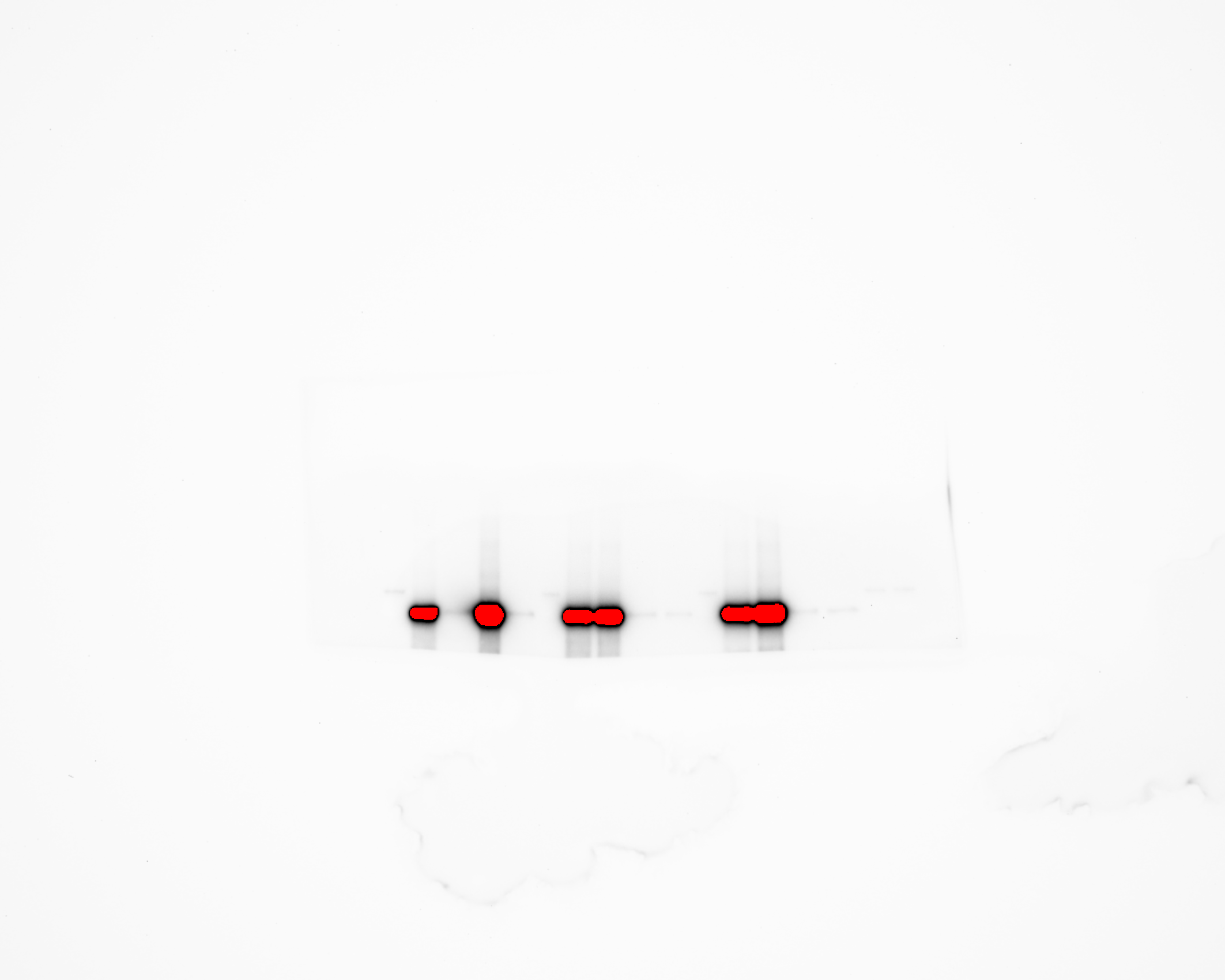

Supplement: Supplementary file 3 — Source data Fig. 2 [file 44318_2026_745_MOESM3_ESM.zip › Figure 2/2H/2H_HA_Elution_Original.tif]

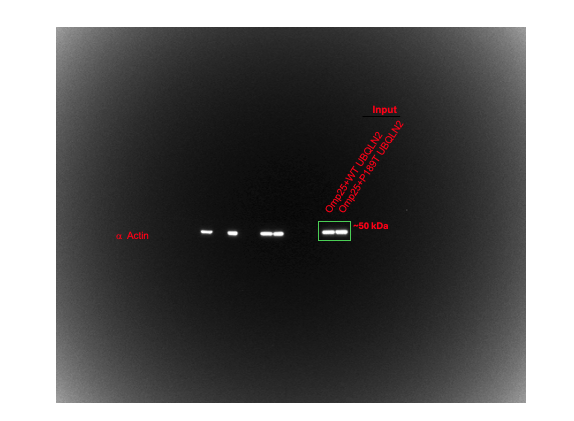

Supplement: Supplementary file 3 — Source data Fig. 2 [file 44318_2026_745_MOESM3_ESM.zip › Figure 2/2H/2H_Actin_Annotated.png]

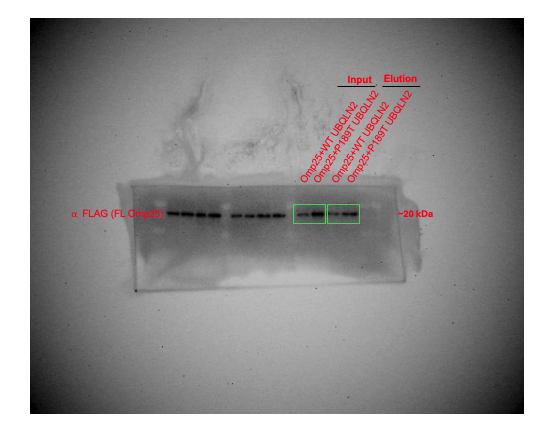

Supplement: Supplementary file 3 — Source data Fig. 2 [file 44318_2026_745_MOESM3_ESM.zip › Figure 2/2H/2H_FLAG_Input_Elution_Annotated.png]

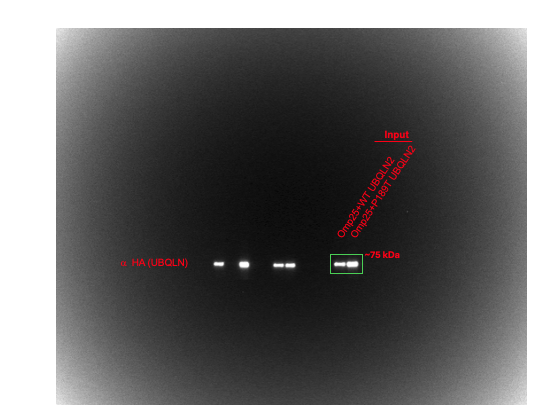

Supplement: Supplementary file 3 — Source data Fig. 2 [file 44318_2026_745_MOESM3_ESM.zip › Figure 2/2H/2H_HA_Input_Annotated.png]

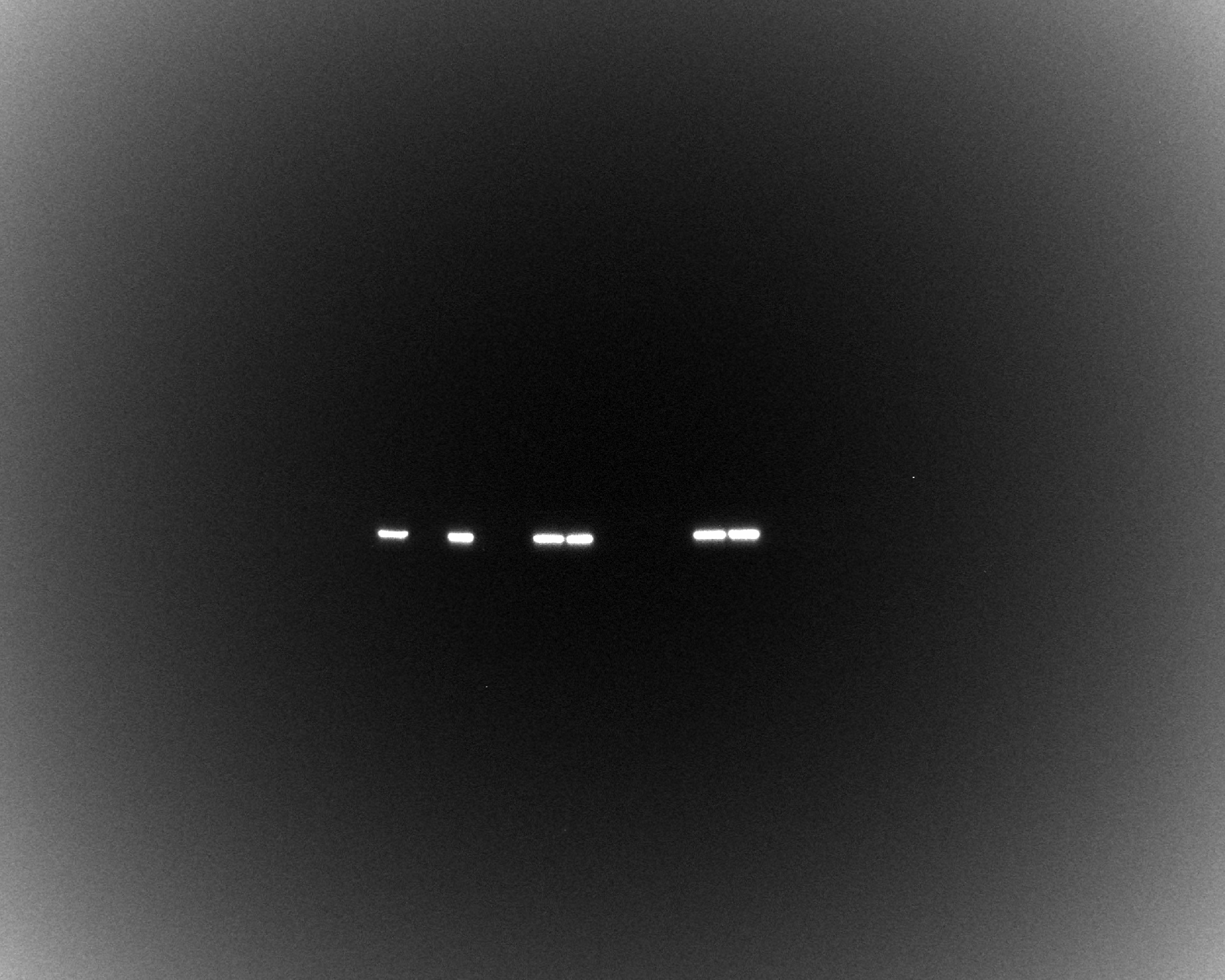

Supplement: Supplementary file 3 — Source data Fig. 2 [file 44318_2026_745_MOESM3_ESM.zip › Figure 2/2H/2H_Actin_Original.tif]

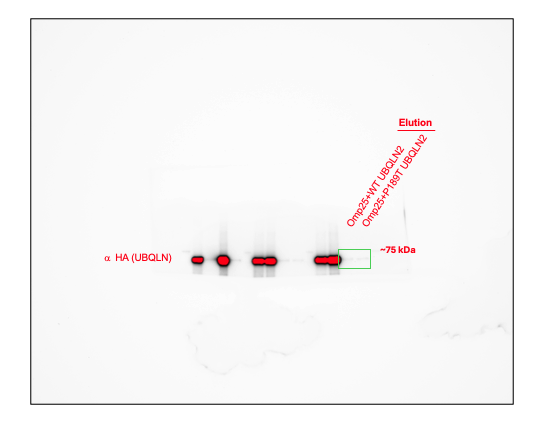

Supplement: Supplementary file 3 — Source data Fig. 2 [file 44318_2026_745_MOESM3_ESM.zip › Figure 2/2H/2H_HA_Elution_Annotated.png]

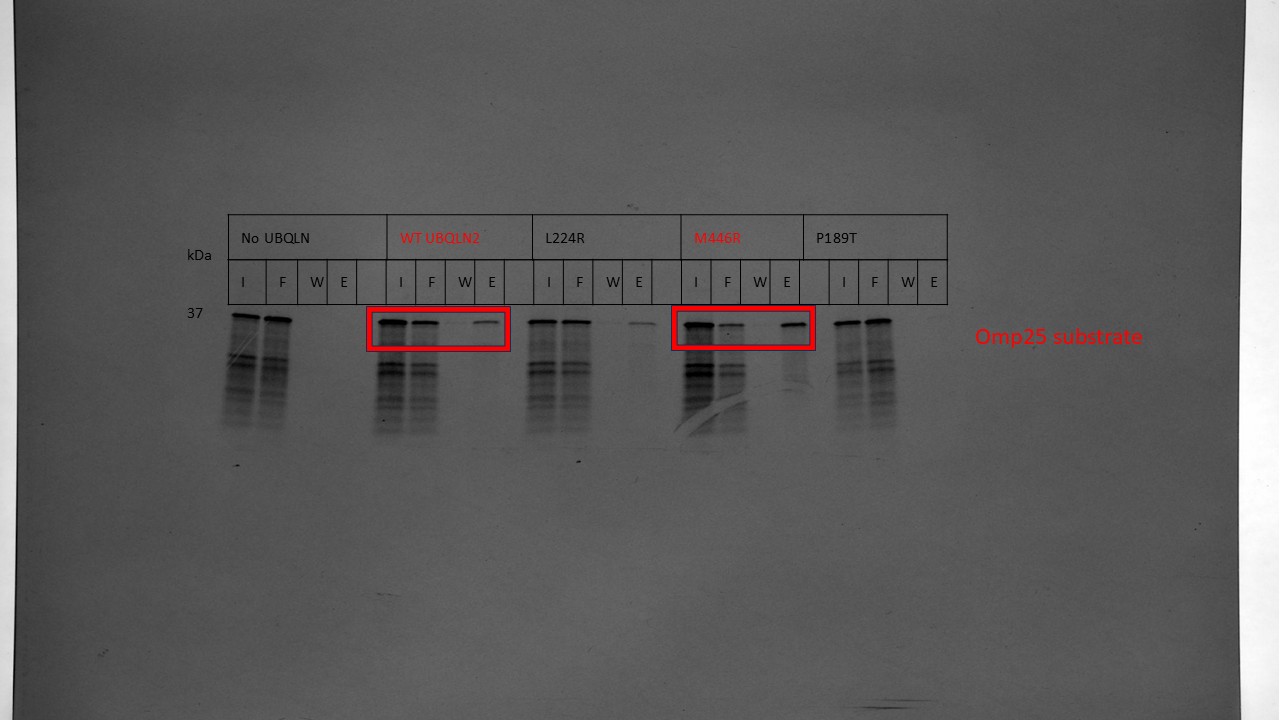

Supplement: Supplementary file 3 — Source data Fig. 2 [file 44318_2026_745_MOESM3_ESM.zip › Figure 2/2F/2F_WT_M446R_annotated.jpg]

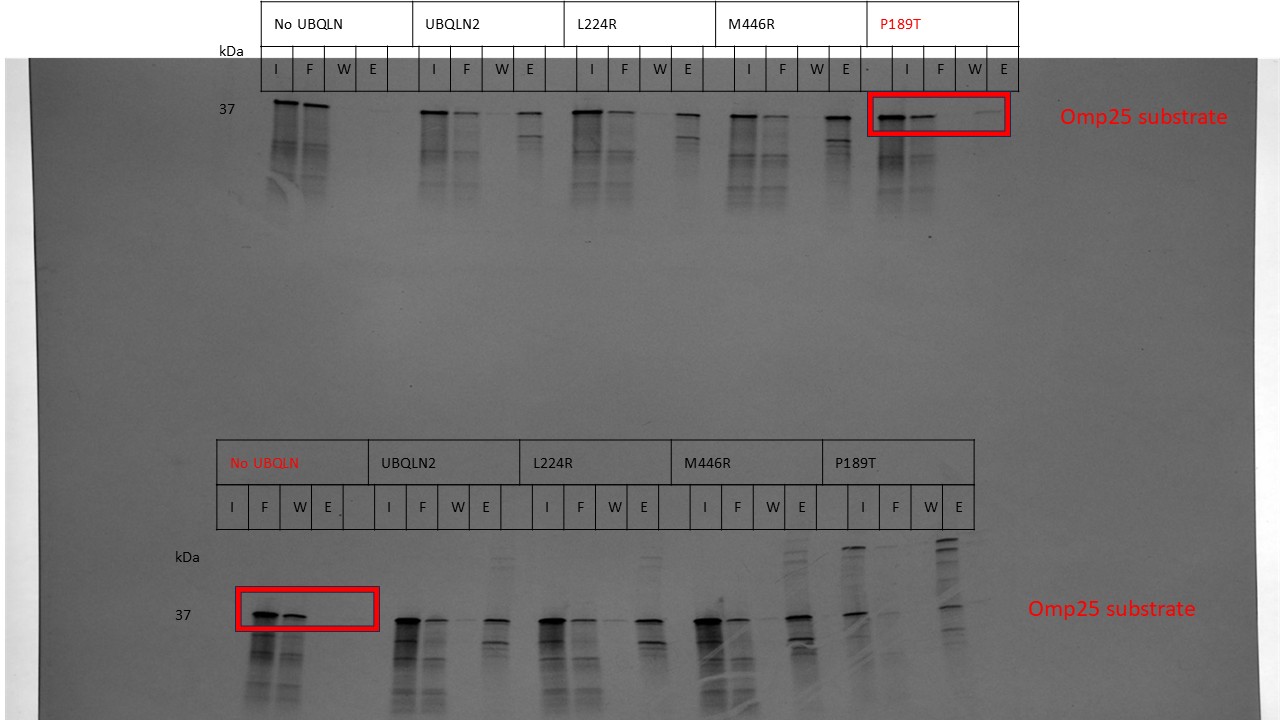

Supplement: Supplementary file 3 — Source data Fig. 2 [file 44318_2026_745_MOESM3_ESM.zip › Figure 2/2F/2F_NoUBQLN_P189T_annotated.jpg]

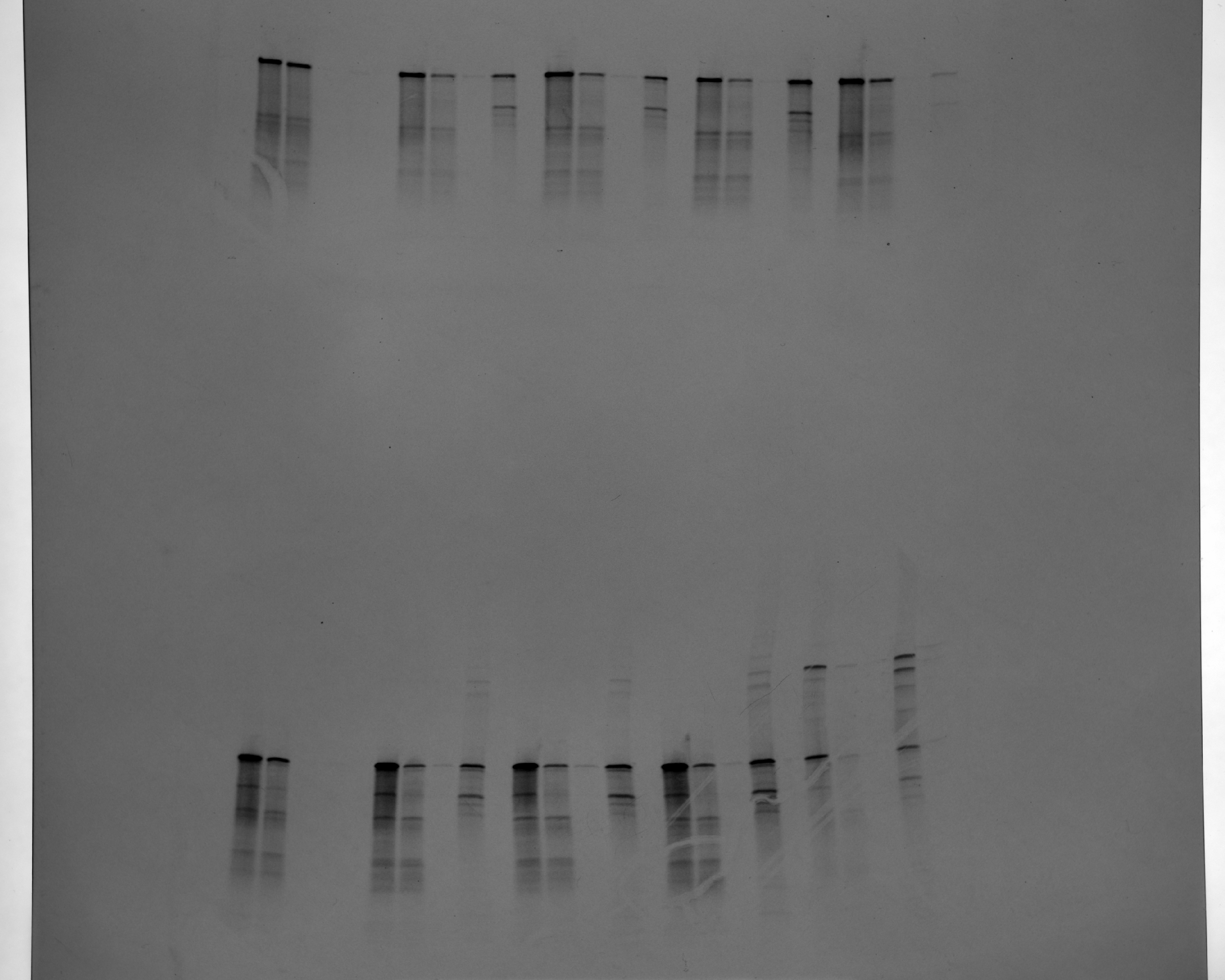

Supplement: Supplementary file 3 — Source data Fig. 2 [file 44318_2026_745_MOESM3_ESM.zip › Figure 2/2F/2F_NoUBQLN_P189T_orginal.jpg]

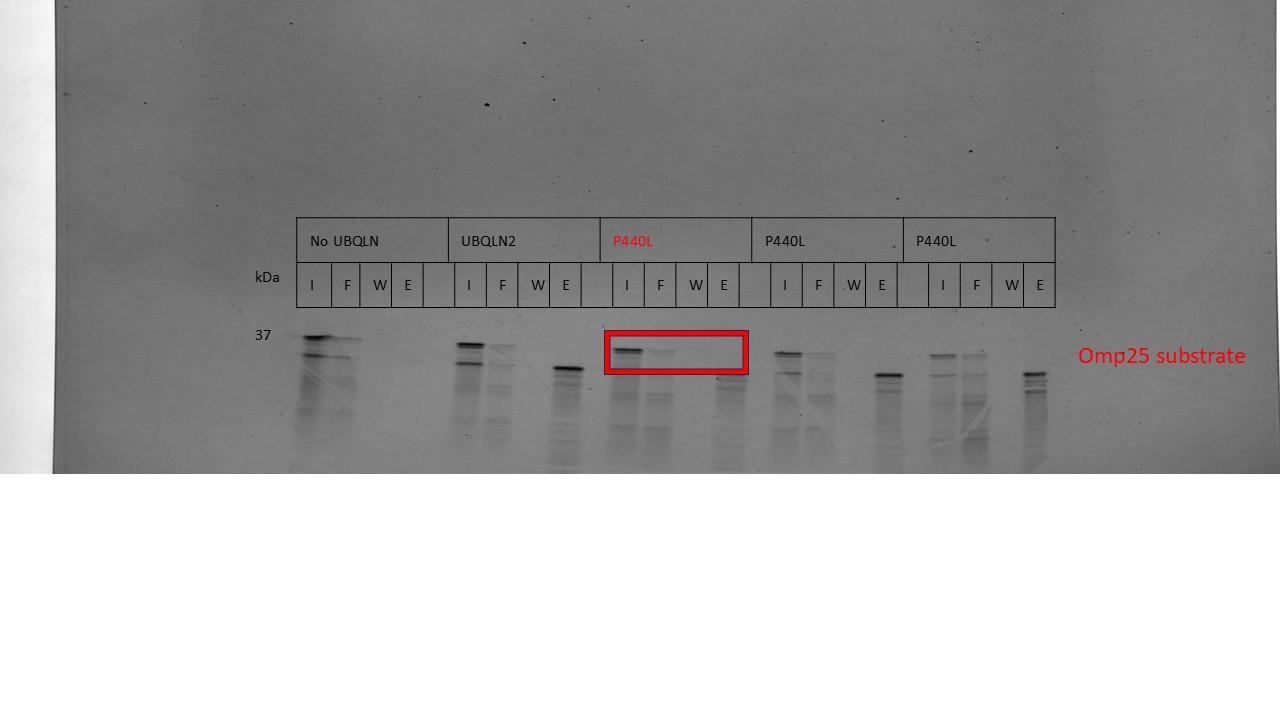

Supplement: Supplementary file 3 — Source data Fig. 2 [file 44318_2026_745_MOESM3_ESM.zip › Figure 2/2F/2F_P440L_annotated.jpg]

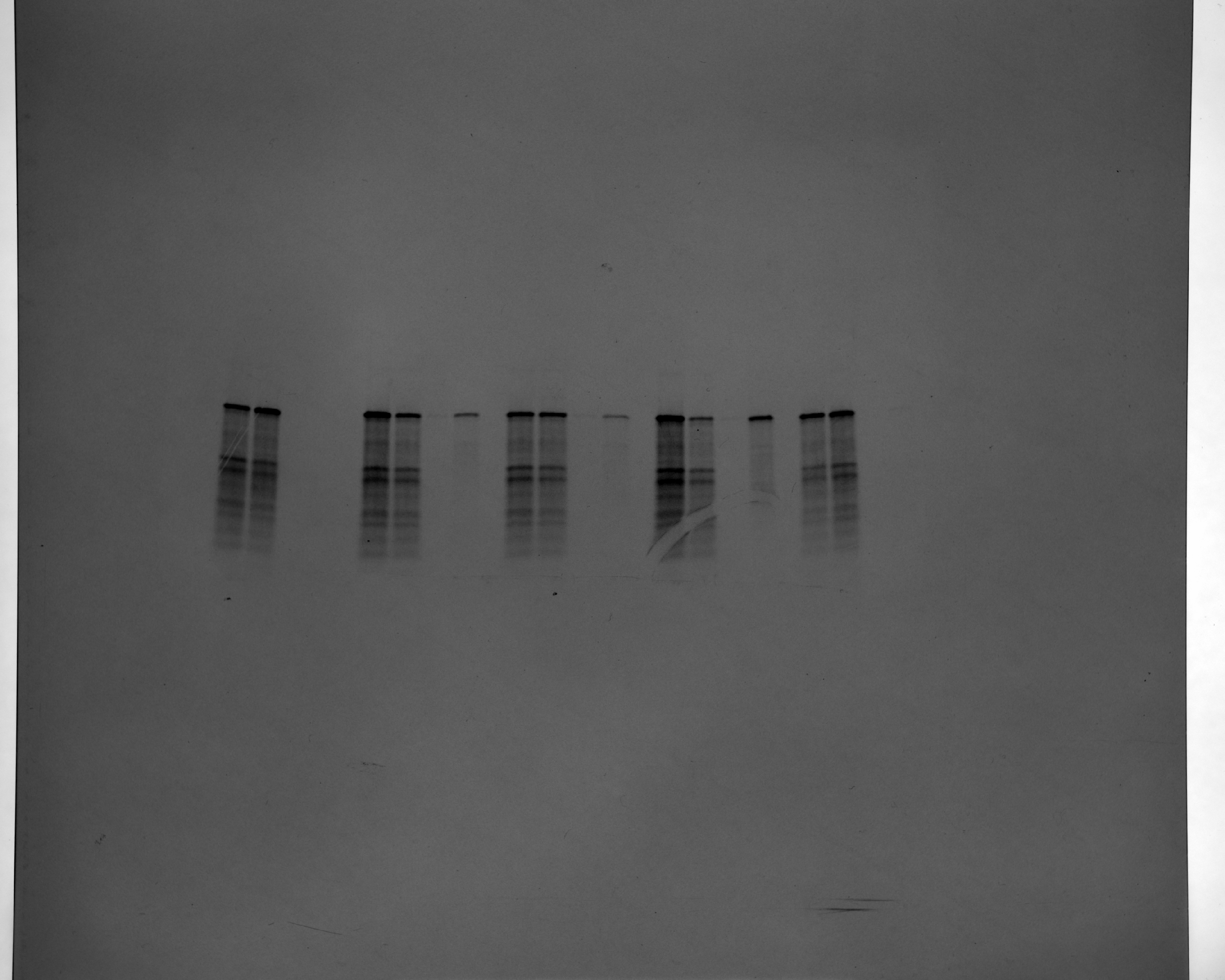

Supplement: Supplementary file 3 — Source data Fig. 2 [file 44318_2026_745_MOESM3_ESM.zip › Figure 2/2F/2F_WT_M446R_Original.jpg]

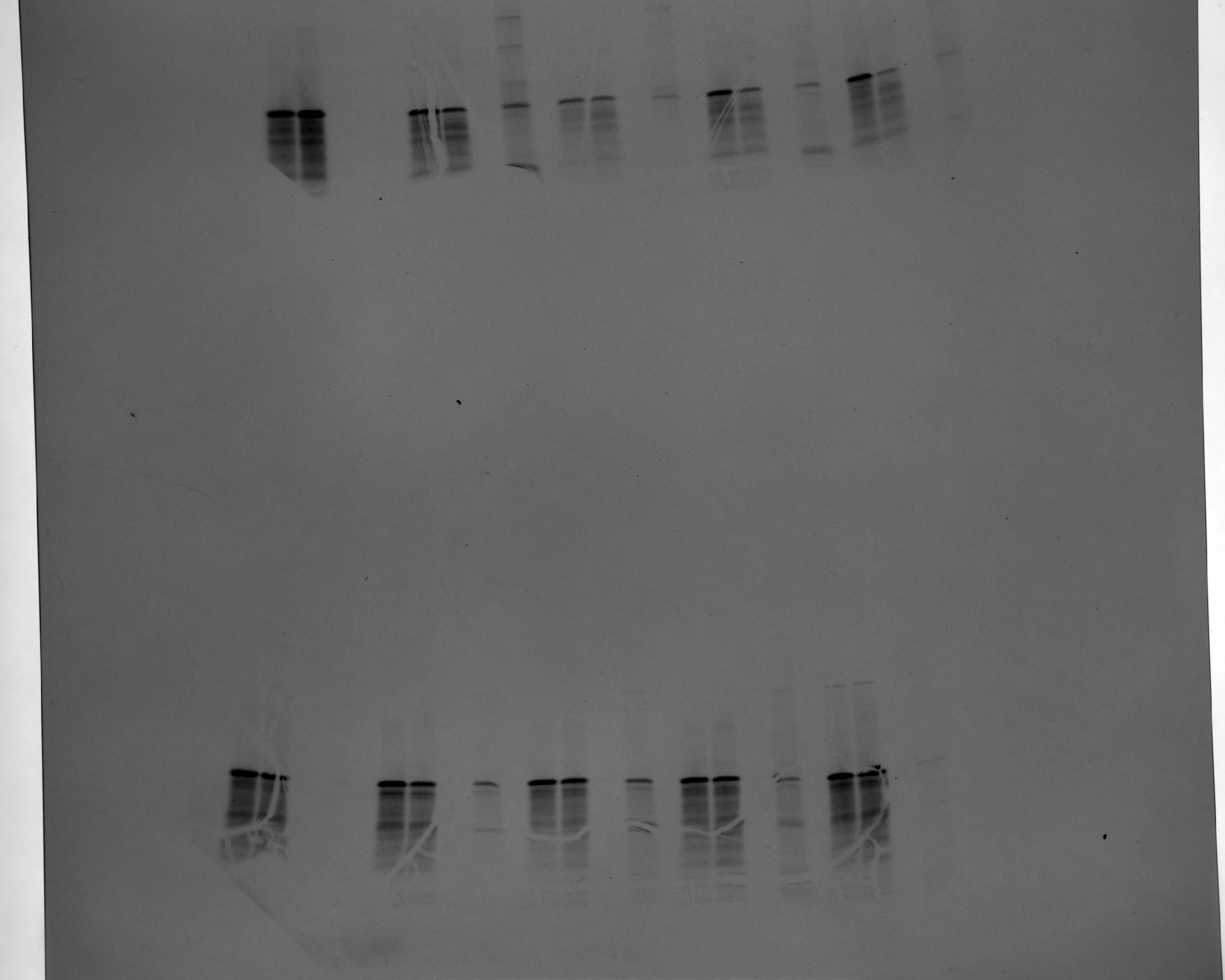

Supplement: Supplementary file 3 — Source data Fig. 2 [file 44318_2026_745_MOESM3_ESM.zip › Figure 2/2F/2F_P440L_orginal.jpg]

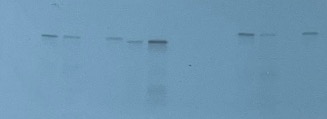

Supplement: Supplementary file 4 — Source data Fig. 3 [file 44318_2026_745_MOESM4_ESM.zip › Figure 3/3B/3B_Original.jpg]

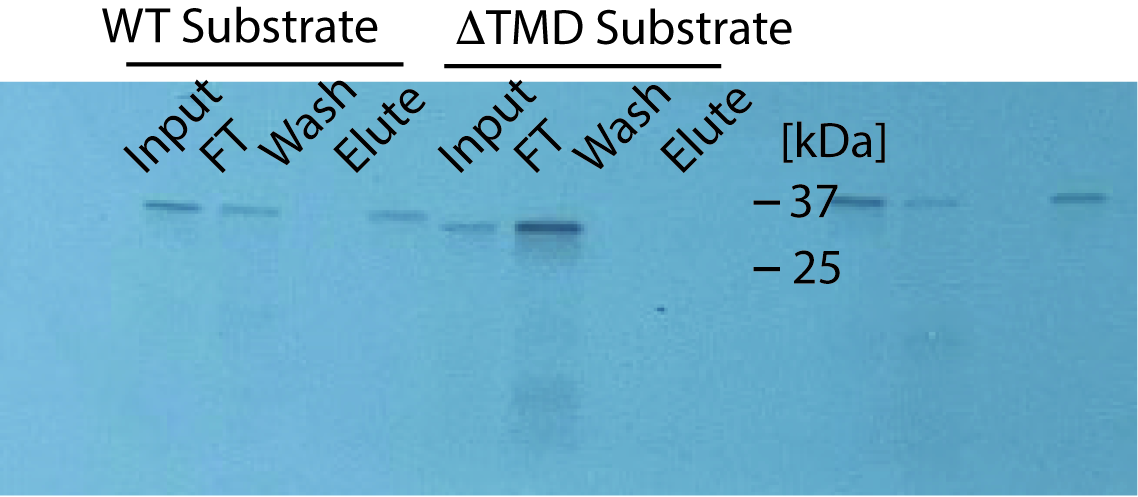

Supplement: Supplementary file 4 — Source data Fig. 3 [file 44318_2026_745_MOESM4_ESM.zip › Figure 3/3B/3B_annotated.tif]

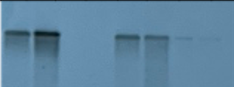

Supplement: Supplementary file 4 — Source data Fig. 3 [file 44318_2026_745_MOESM4_ESM.zip › Figure 3/3C/3C_original.png]

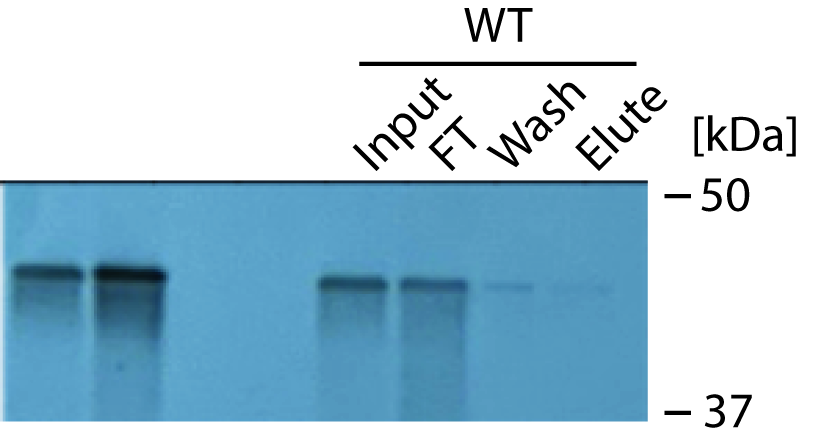

Supplement: Supplementary file 4 — Source data Fig. 3 [file 44318_2026_745_MOESM4_ESM.zip › Figure 3/3C/3C_annotated.tif]

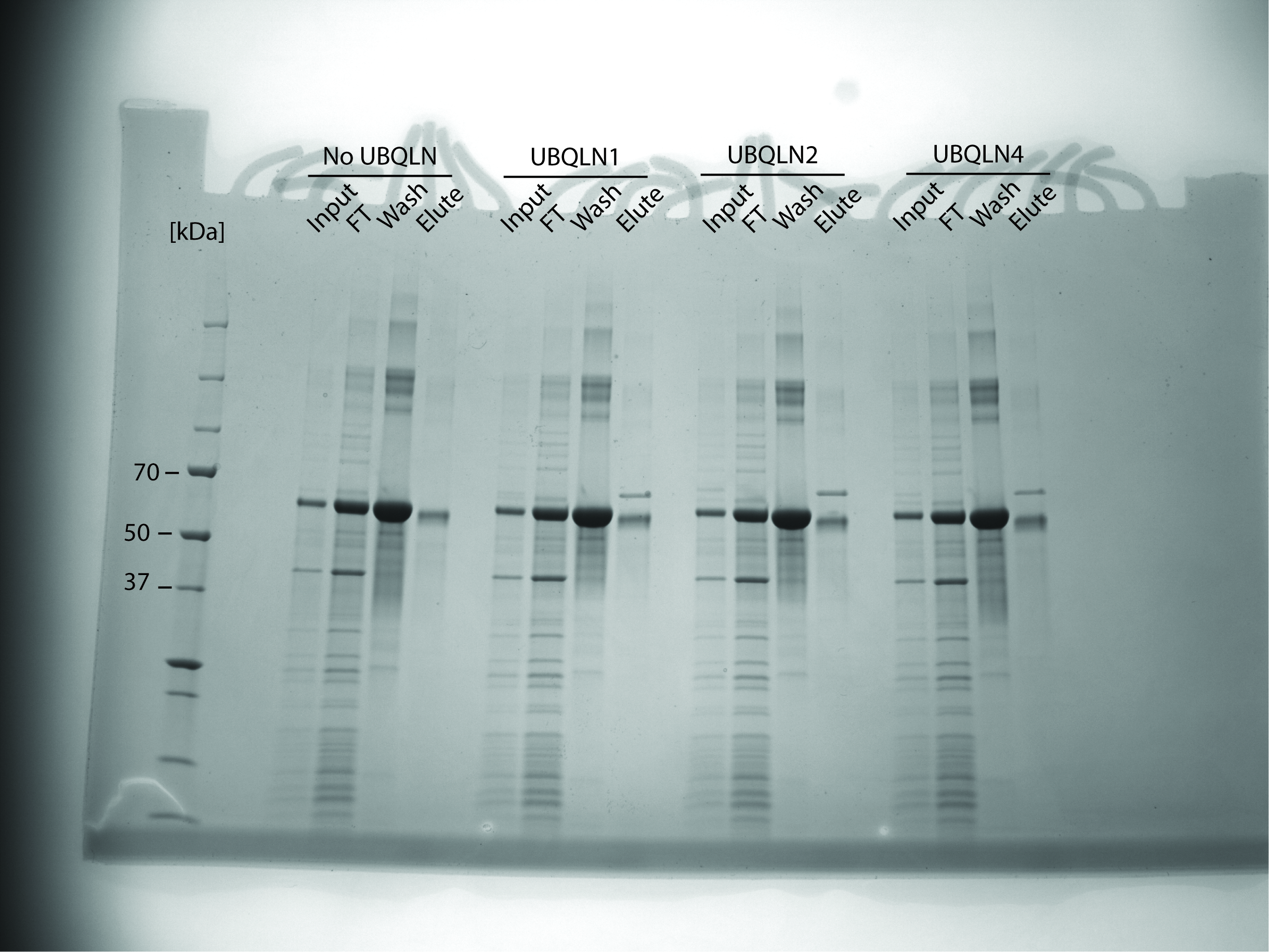

Supplement: Supplementary file 4 — Source data Fig. 3 [file 44318_2026_745_MOESM4_ESM.zip › Figure 3/3D/3D_gel_annotated.tif]

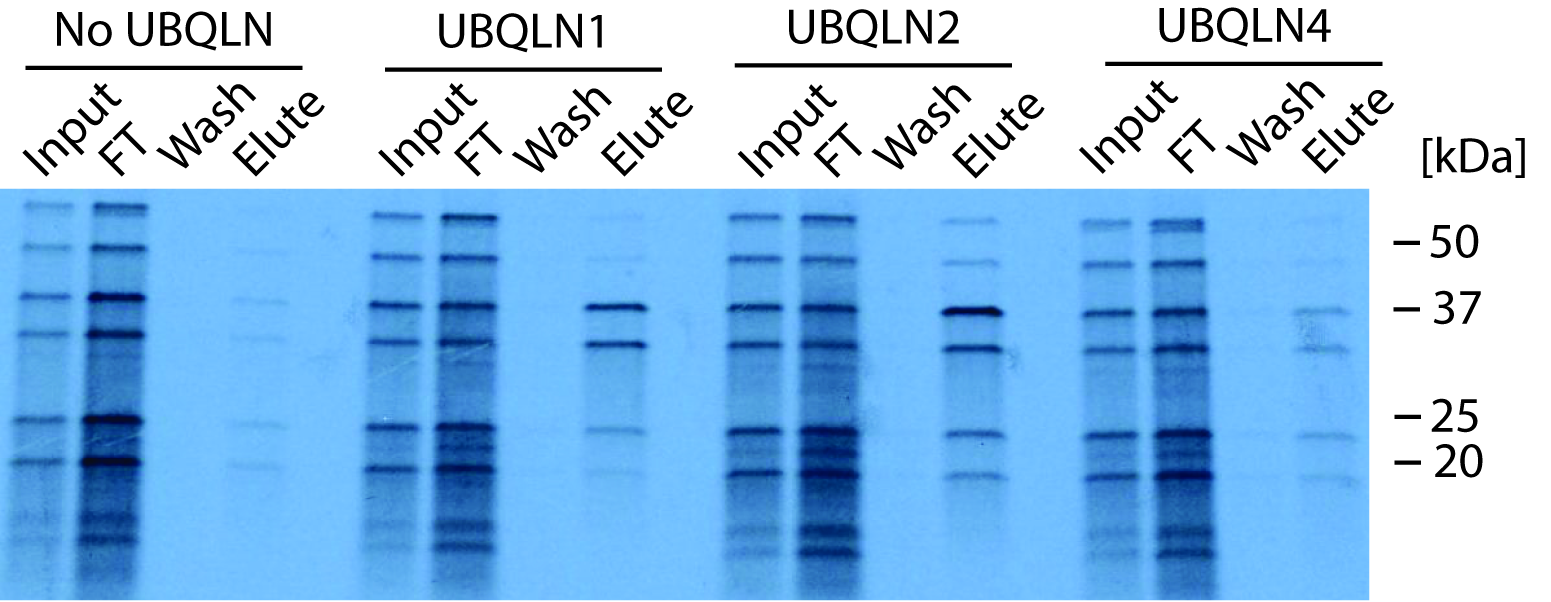

Supplement: Supplementary file 4 — Source data Fig. 3 [file 44318_2026_745_MOESM4_ESM.zip › Figure 3/3D/3D_film_annotated.tif]

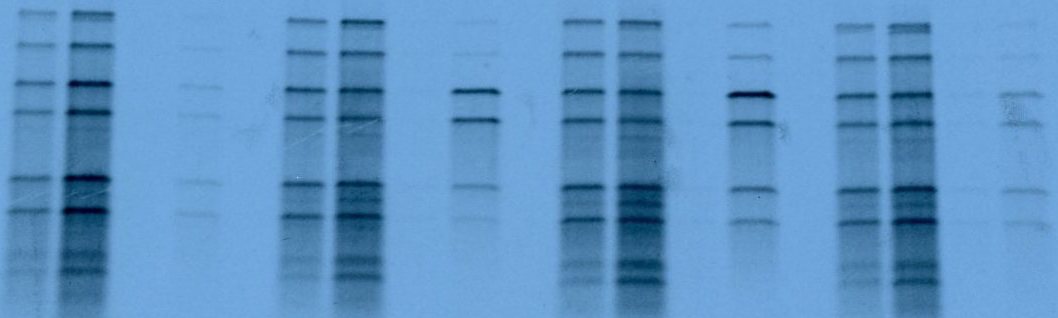

Supplement: Supplementary file 4 — Source data Fig. 3 [file 44318_2026_745_MOESM4_ESM.zip › Figure 3/3D/3D_film_original.jpg]

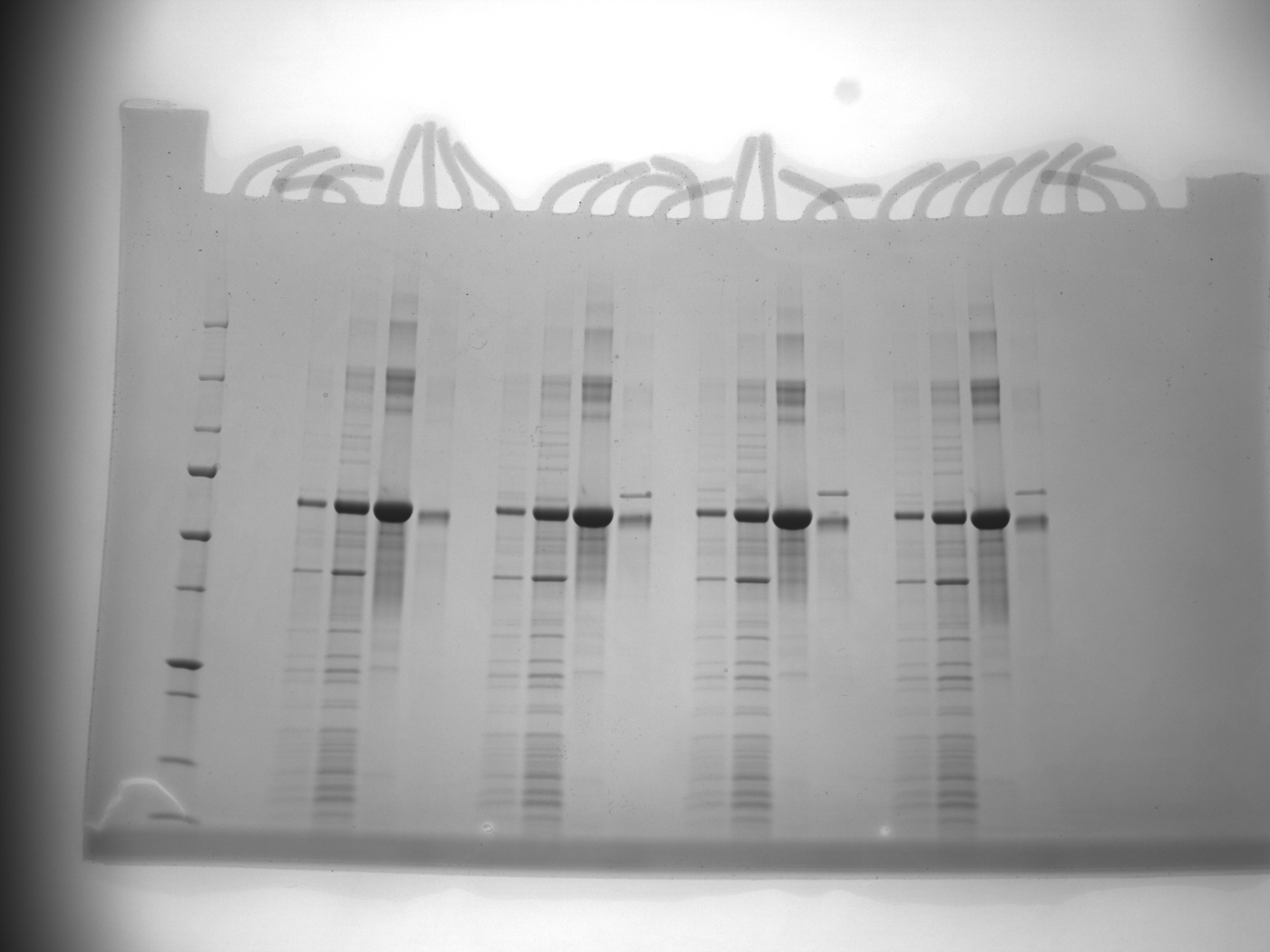

Supplement: Supplementary file 4 — Source data Fig. 3 [file 44318_2026_745_MOESM4_ESM.zip › Figure 3/3D/3D_gel_original.jpg]

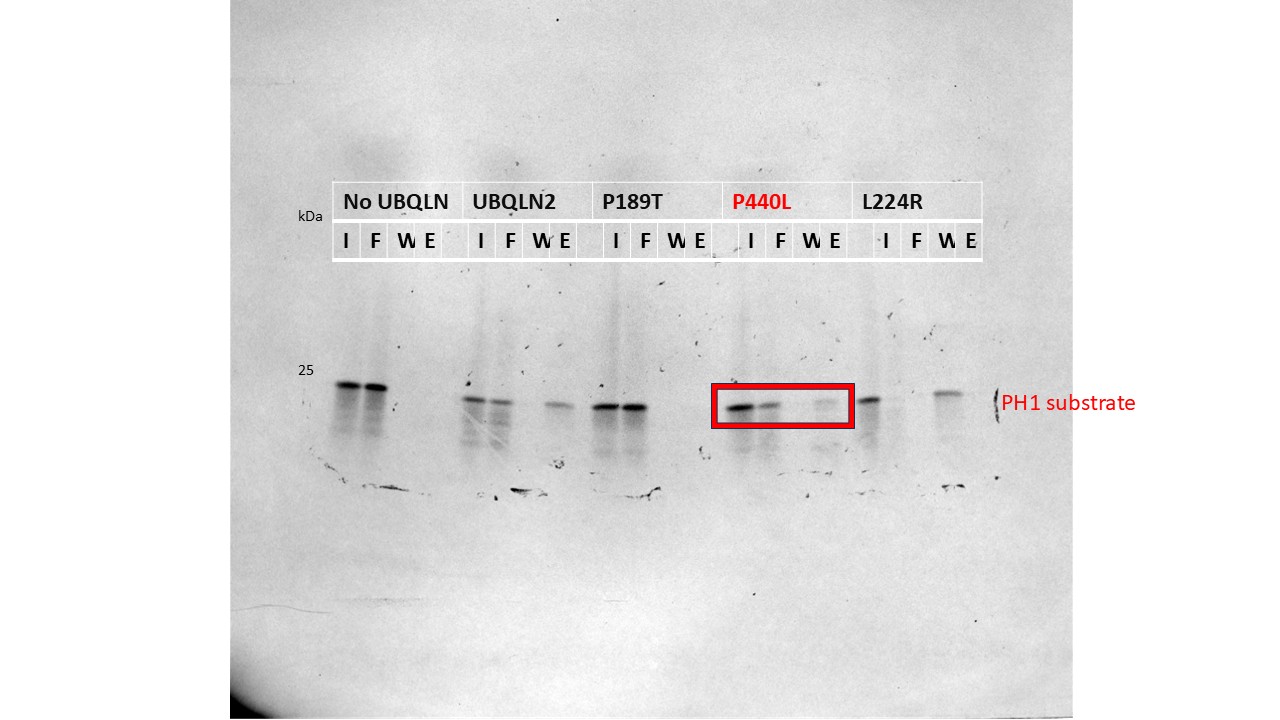

Supplement: Supplementary file 6 — Source data Fig. 5 [file 44318_2026_745_MOESM6_ESM.zip › Figure 5/5C/5C_PH1_P440L_Annotated.jpg]

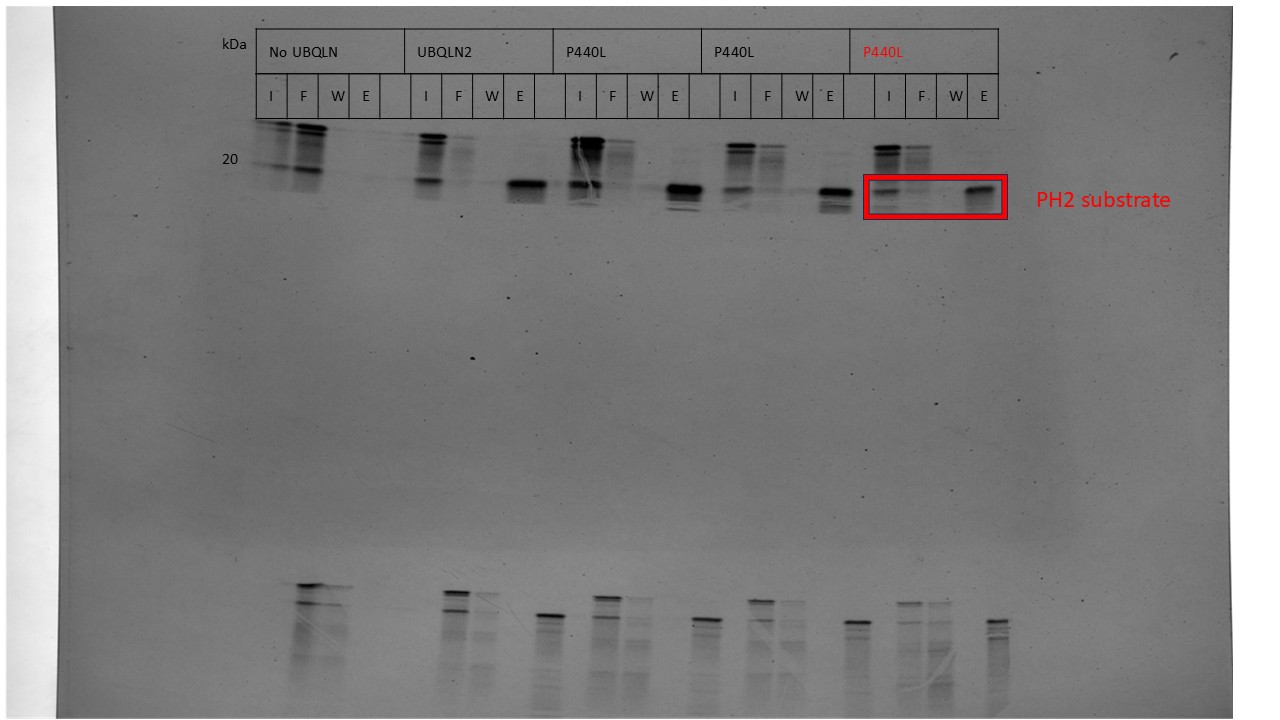

Supplement: Supplementary file 6 — Source data Fig. 5 [file 44318_2026_745_MOESM6_ESM.zip › Figure 5/5C/5C_PH2_P440L_annotated.jpg]

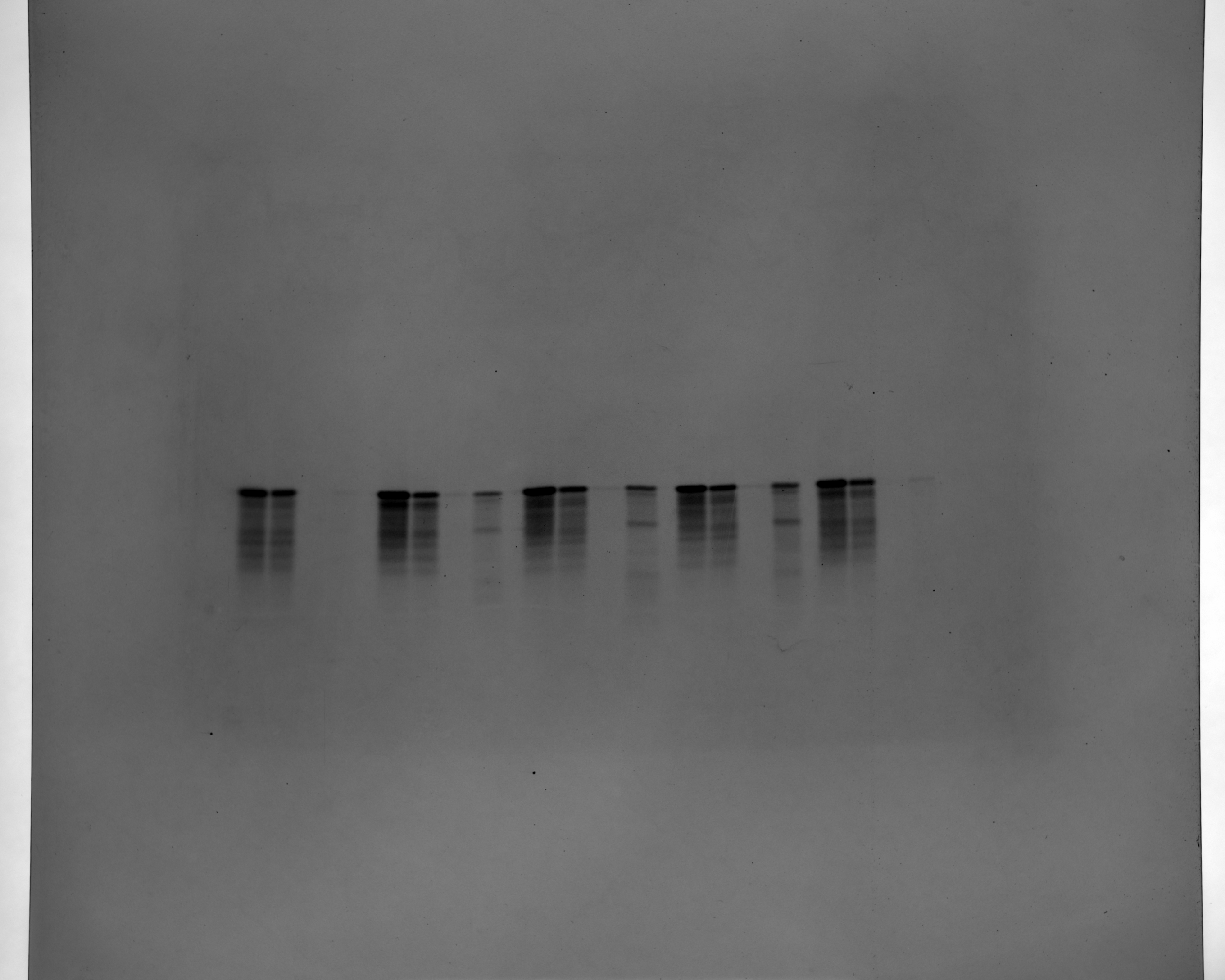

Supplement: Supplementary file 6 — Source data Fig. 5 [file 44318_2026_745_MOESM6_ESM.zip › Figure 5/5C/5C_PH1_NoUBQLN_WT_M446R_P189T_orginal.jpg]

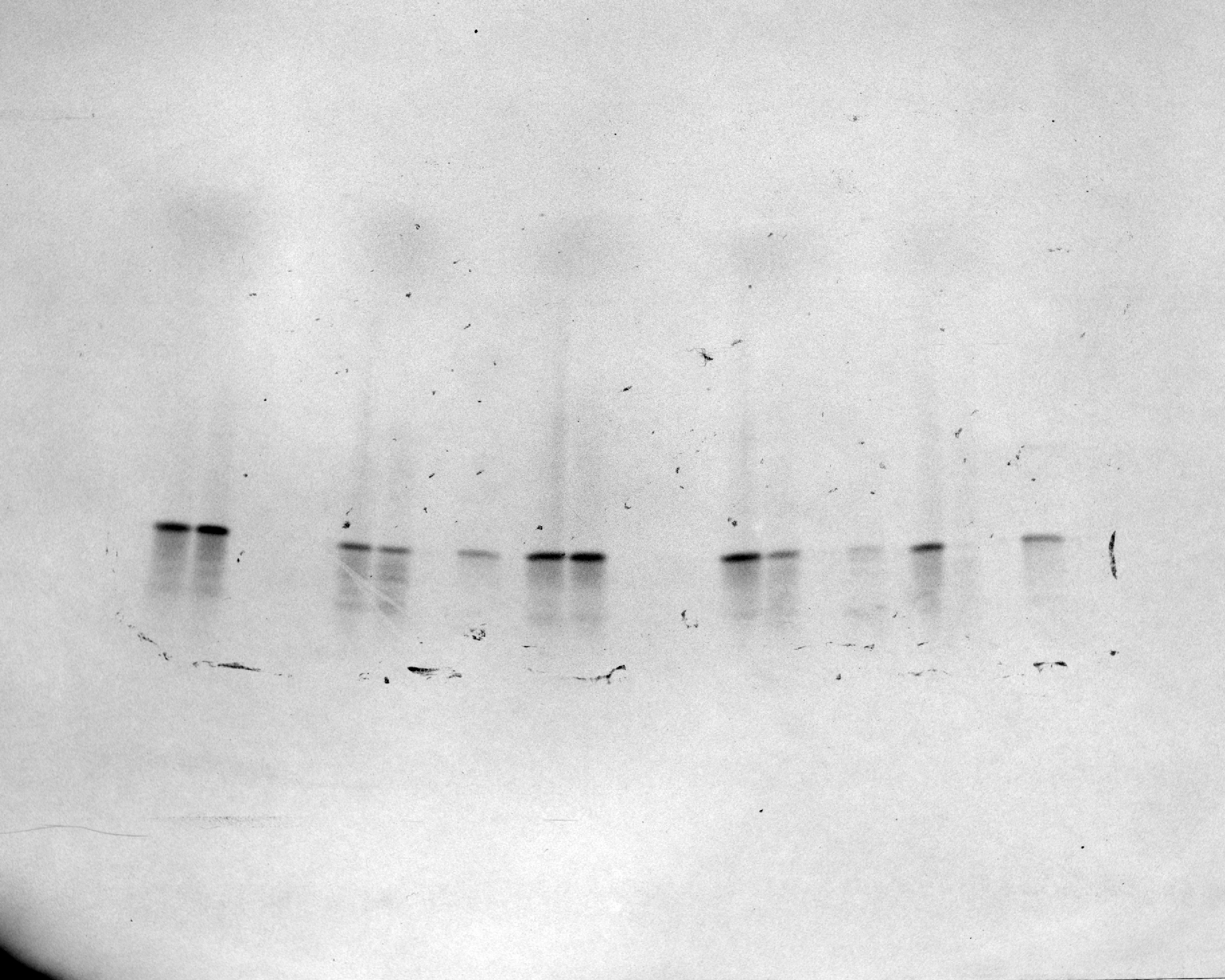

Supplement: Supplementary file 6 — Source data Fig. 5 [file 44318_2026_745_MOESM6_ESM.zip › Figure 5/5C/5C_PH1_P440L_Orginal.jpg]

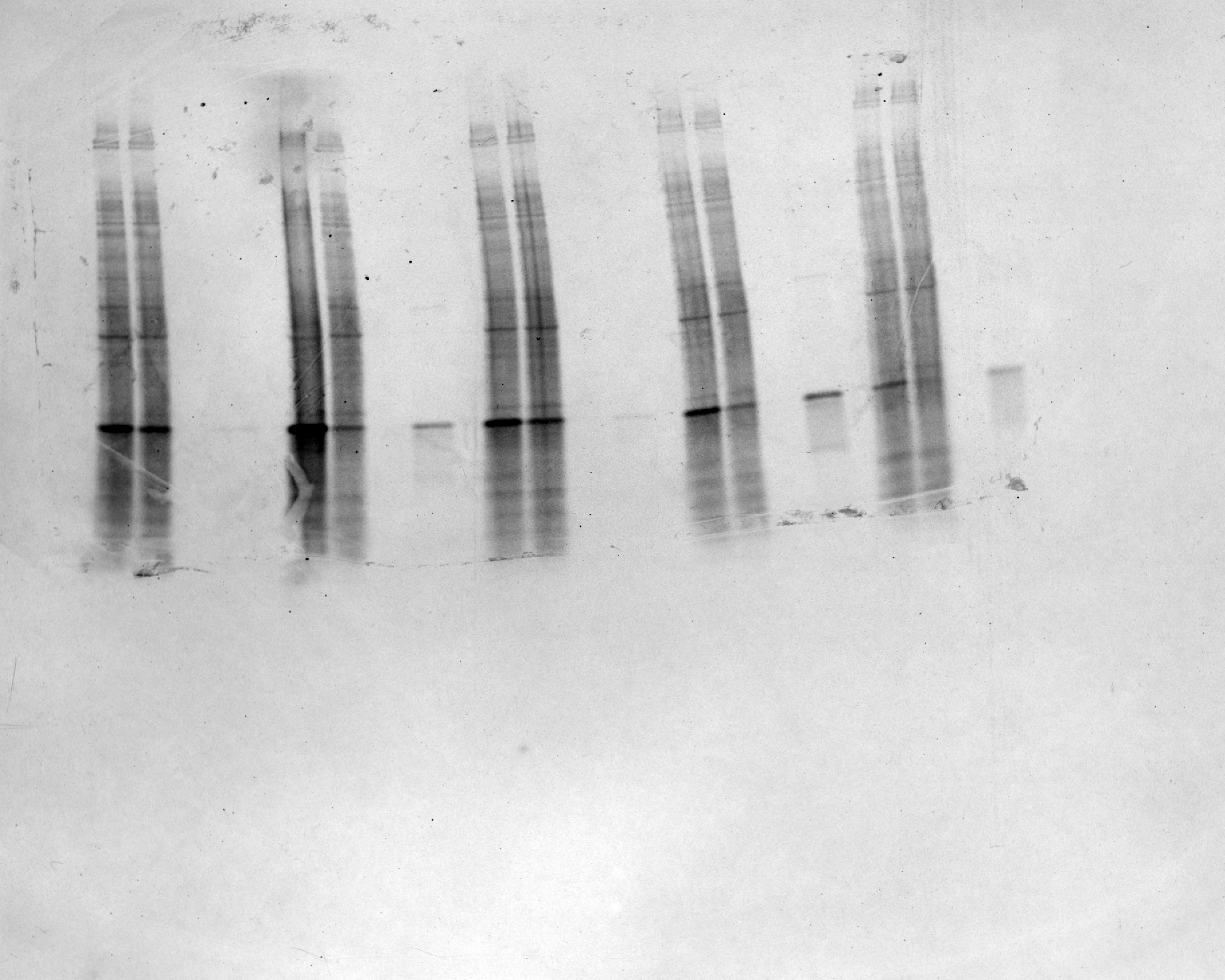

Supplement: Supplementary file 6 — Source data Fig. 5 [file 44318_2026_745_MOESM6_ESM.zip › Figure 5/5C/5C_PH2_NoUBQLN_Original.jpg]

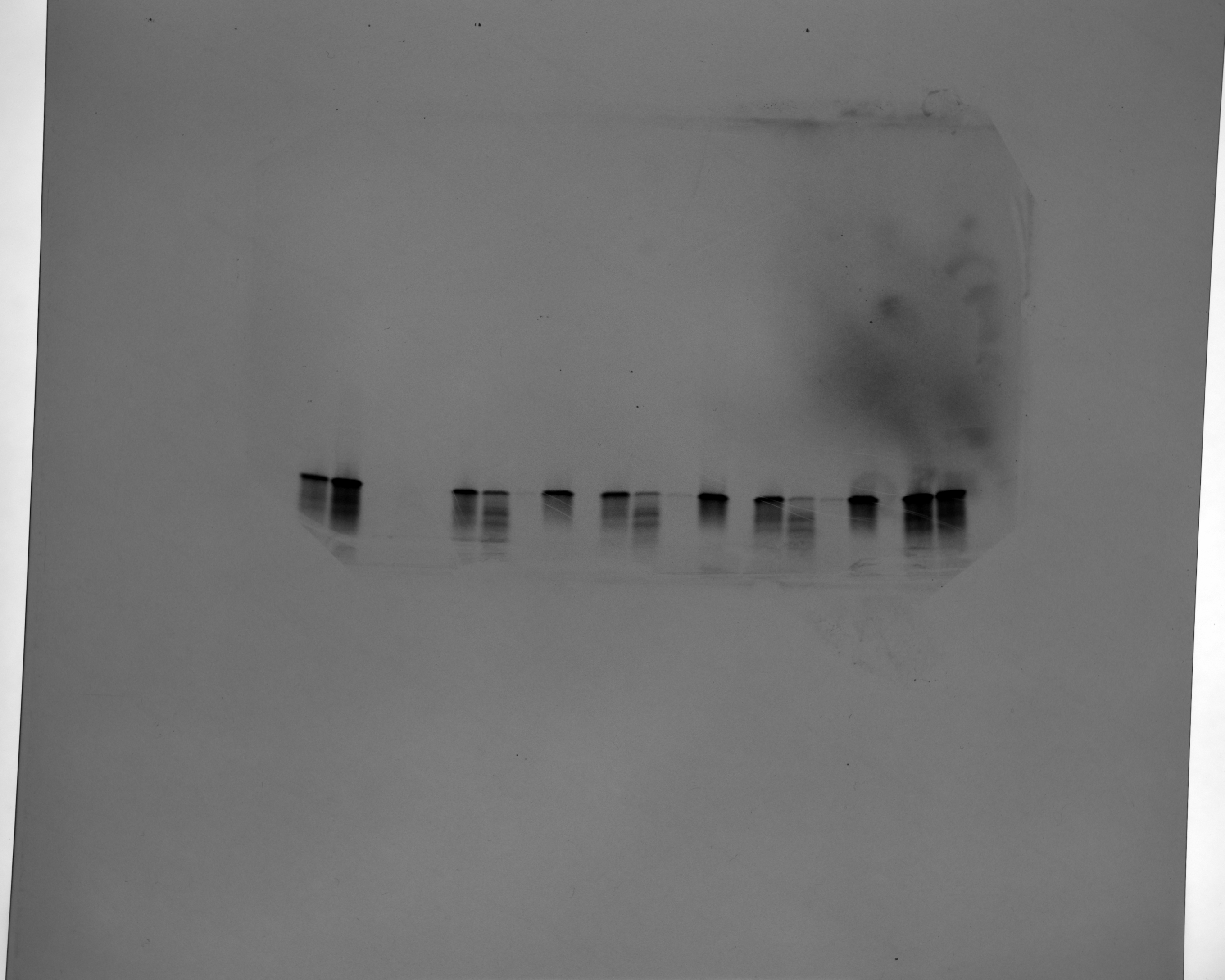

Supplement: Supplementary file 6 — Source data Fig. 5 [file 44318_2026_745_MOESM6_ESM.zip › Figure 5/5C/5C_PH2_WT_M446R_P189T_Orginal.jpg]

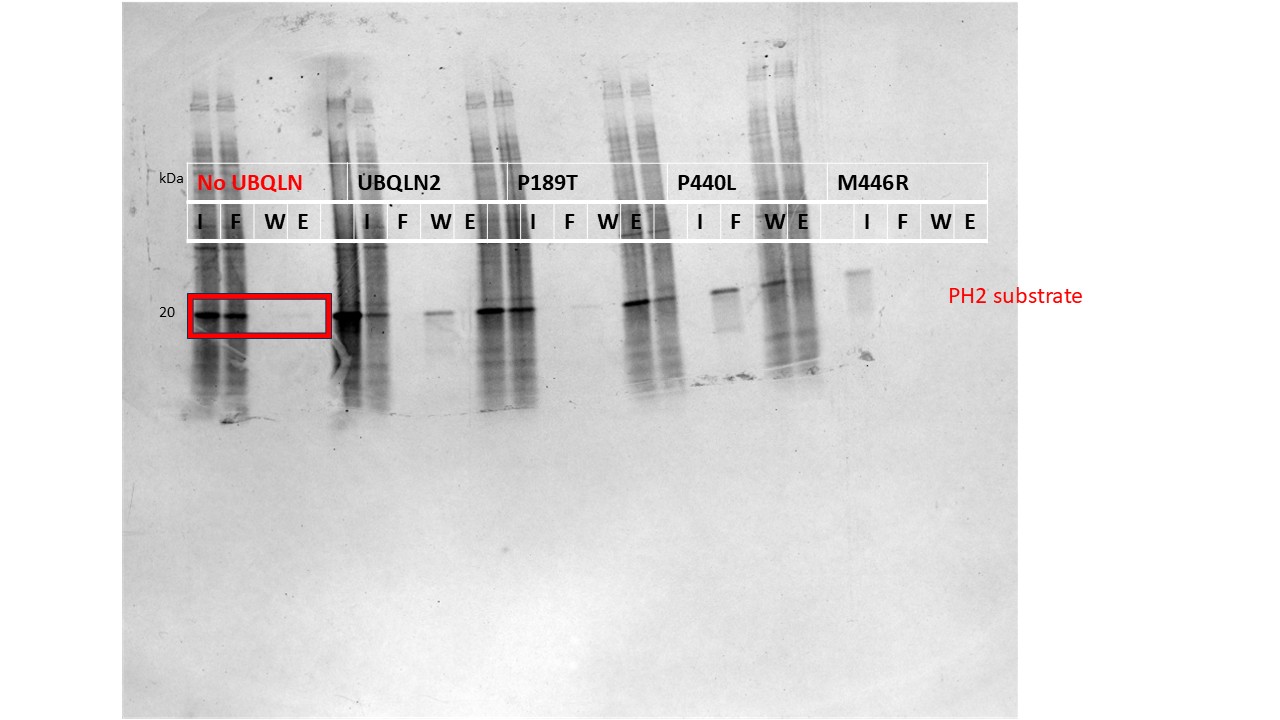

Supplement: Supplementary file 6 — Source data Fig. 5 [file 44318_2026_745_MOESM6_ESM.zip › Figure 5/5C/5C_PH2_NoUBQLN_annotated.jpg]

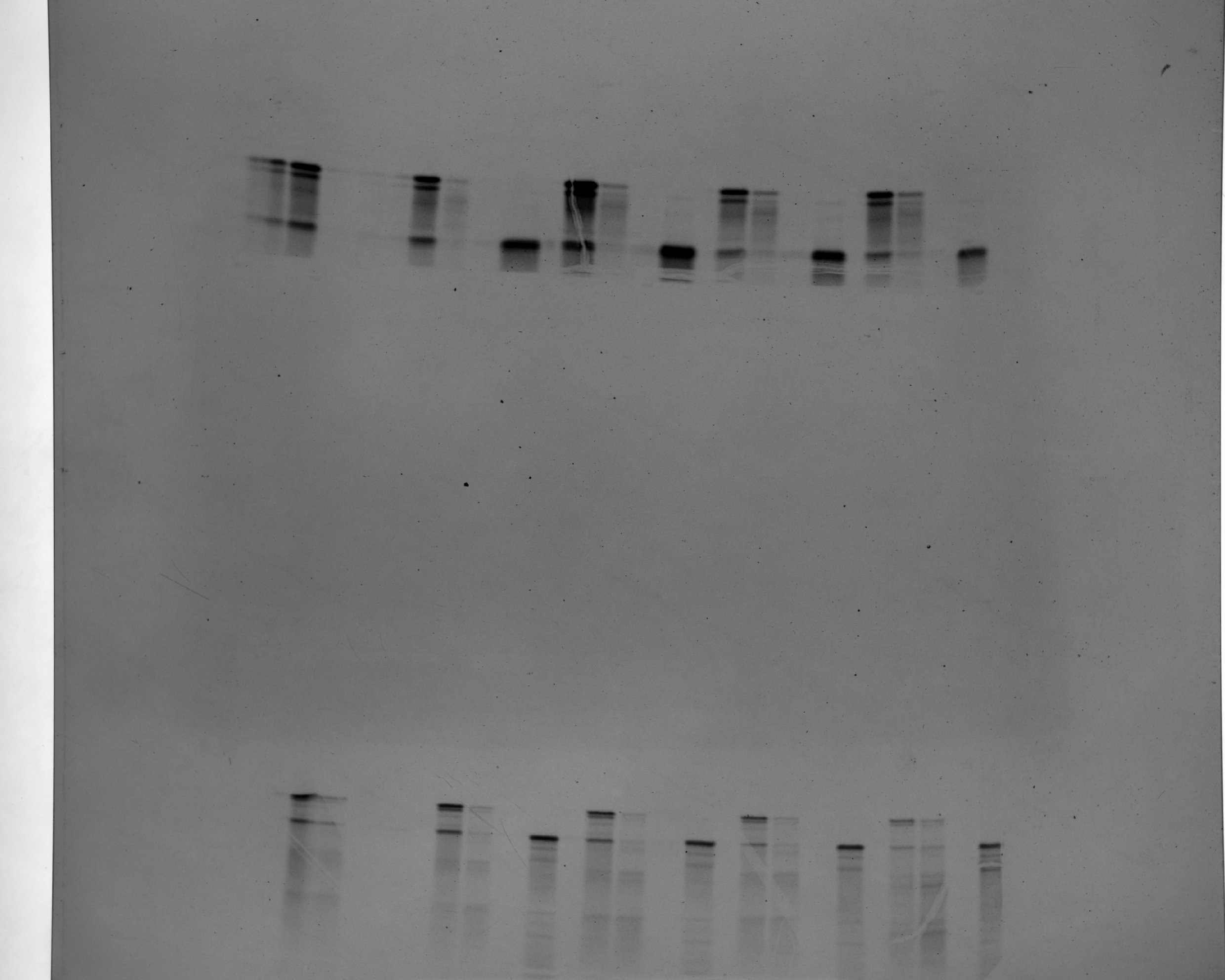

Supplement: Supplementary file 6 — Source data Fig. 5 [file 44318_2026_745_MOESM6_ESM.zip › Figure 5/5C/5C_PH2_P440L_orginal.jpg]

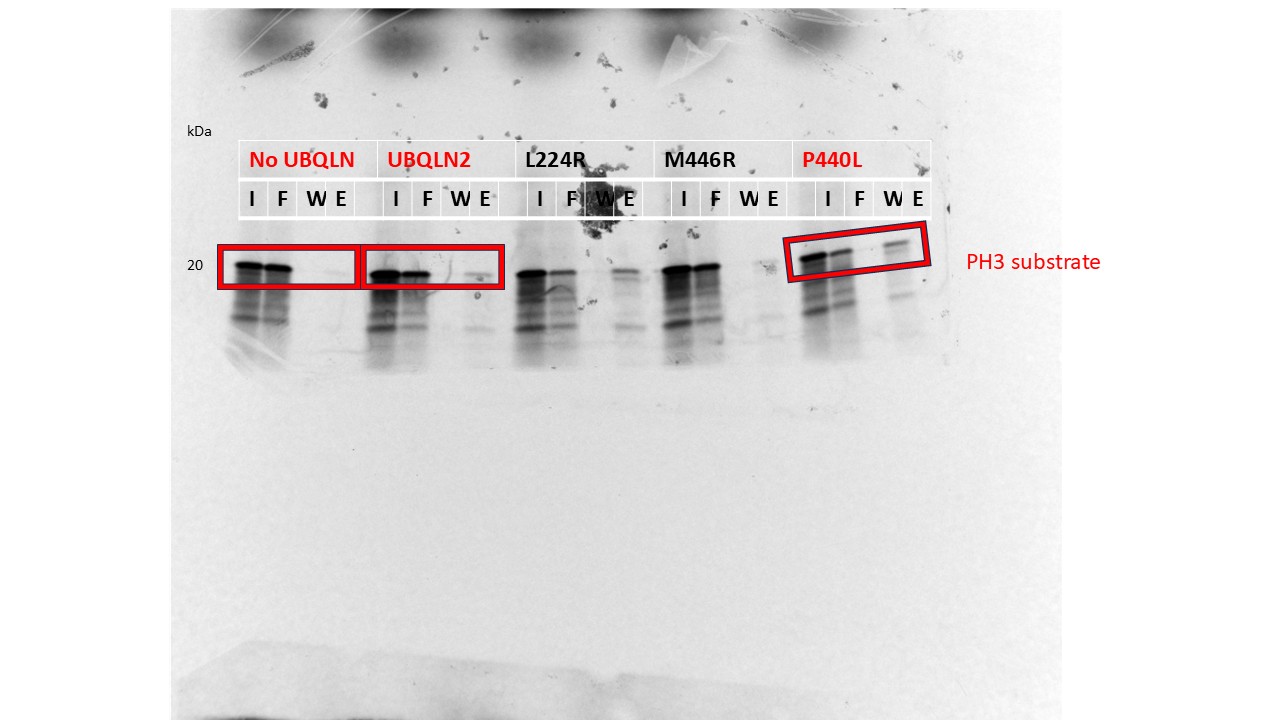

Supplement: Supplementary file 6 — Source data Fig. 5 [file 44318_2026_745_MOESM6_ESM.zip › Figure 5/5C/5C_PH3_NOUBQLN_WT_P440L_annotated.jpg]

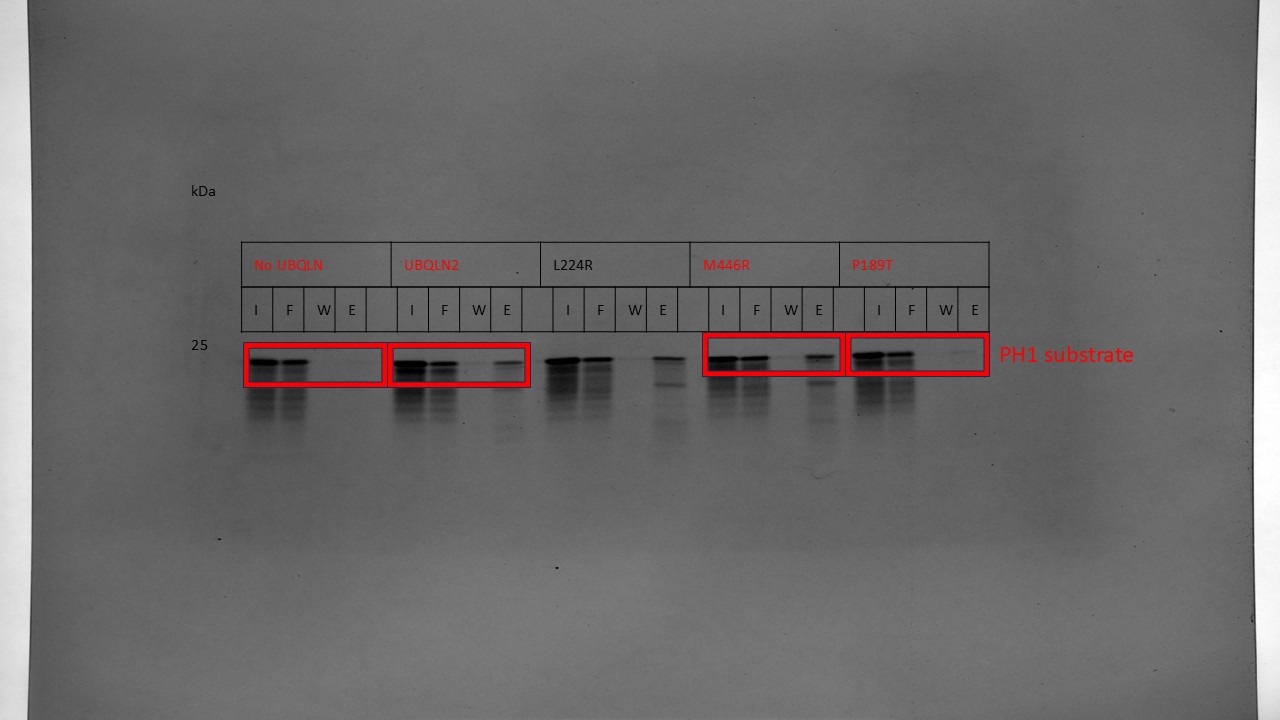

Supplement: Supplementary file 6 — Source data Fig. 5 [file 44318_2026_745_MOESM6_ESM.zip › Figure 5/5C/5C_PH1_NoUBQLN_WT_M446R_P189T_annotated.jpg]

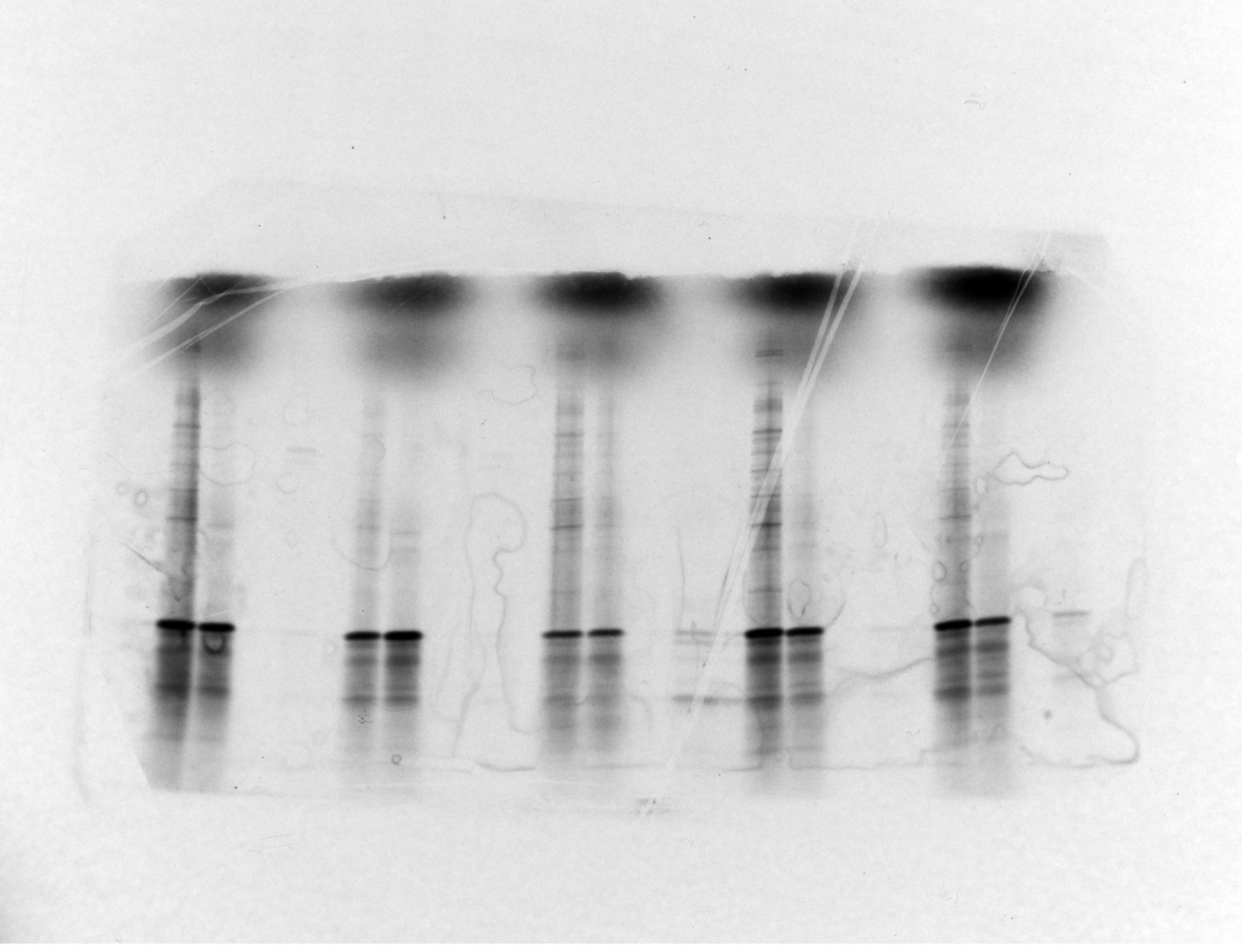

Supplement: Supplementary file 6 — Source data Fig. 5 [file 44318_2026_745_MOESM6_ESM.zip › Figure 5/5C/5C_PH3_NOUBQLN_WT_P440L_Orginal.jpg]

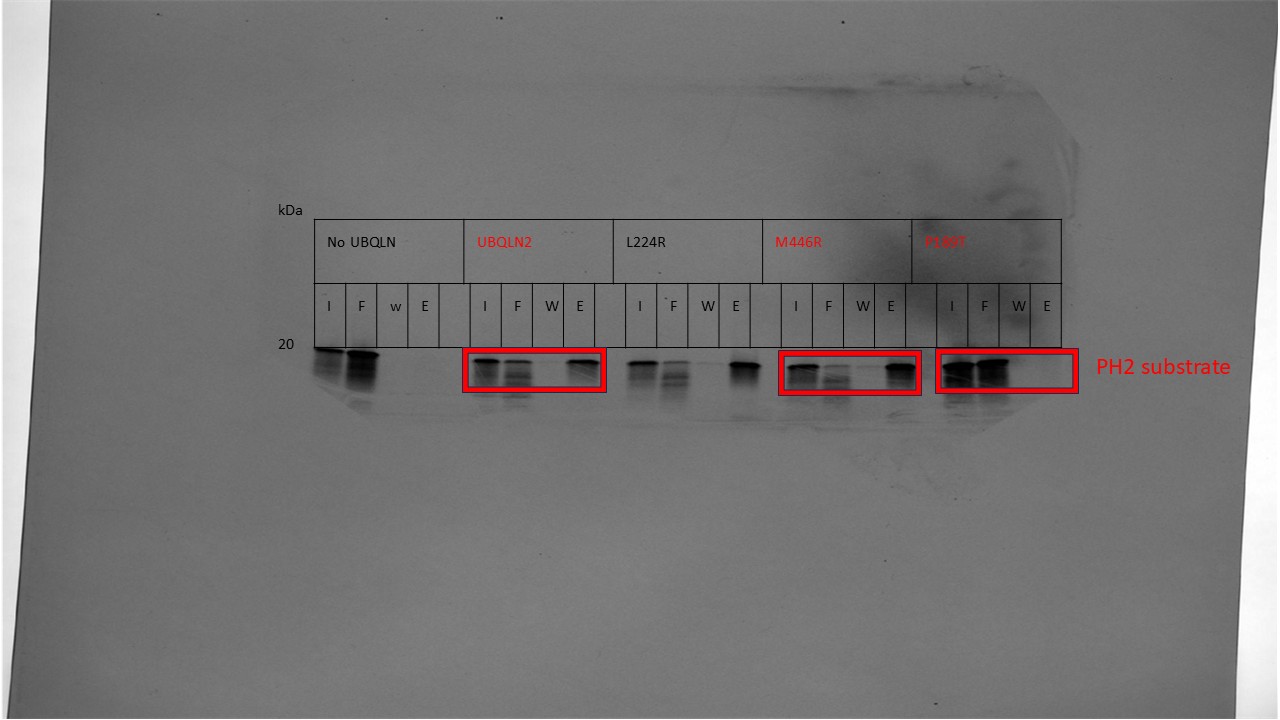

Supplement: Supplementary file 6 — Source data Fig. 5 [file 44318_2026_745_MOESM6_ESM.zip › Figure 5/5C/5C_PH2_WT_M446R_P189T_Annotated.jpg]

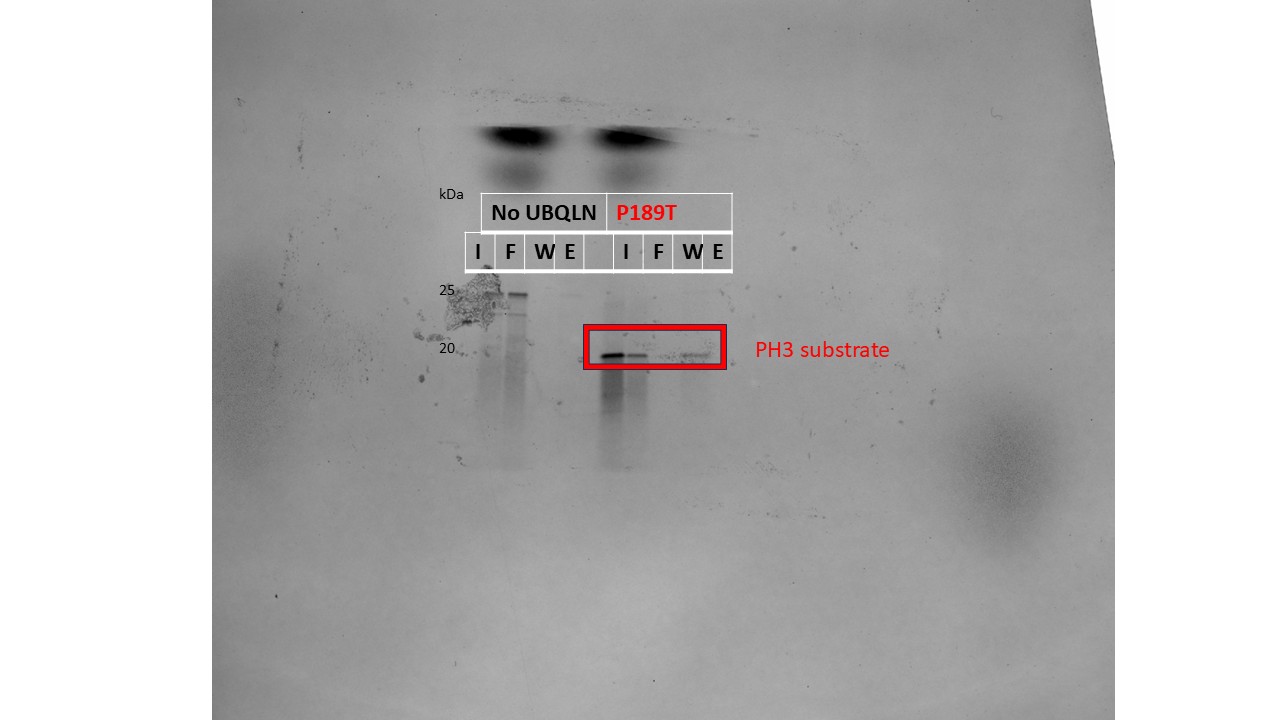

Supplement: Supplementary file 6 — Source data Fig. 5 [file 44318_2026_745_MOESM6_ESM.zip › Figure 5/5C/5C_PH3_P189T_annotated.jpg]

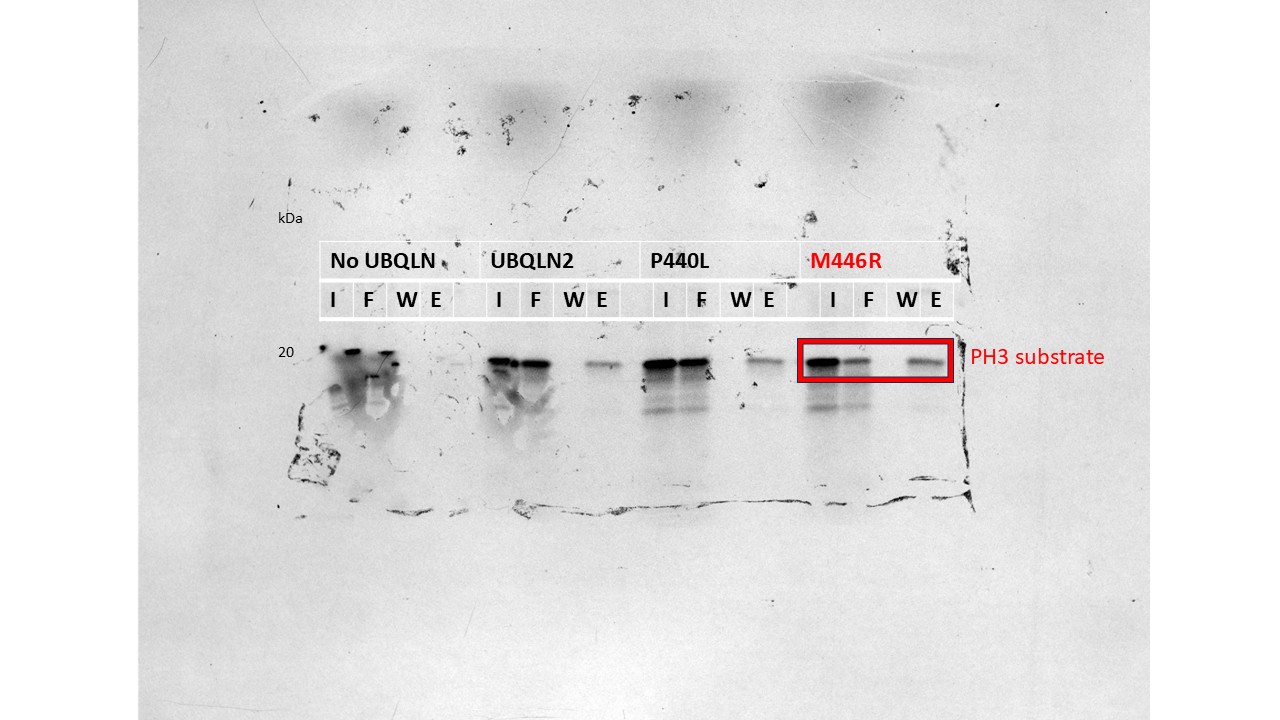

Supplement: Supplementary file 6 — Source data Fig. 5 [file 44318_2026_745_MOESM6_ESM.zip › Figure 5/5C/5C_PH3_M446R_annotated.jpg]

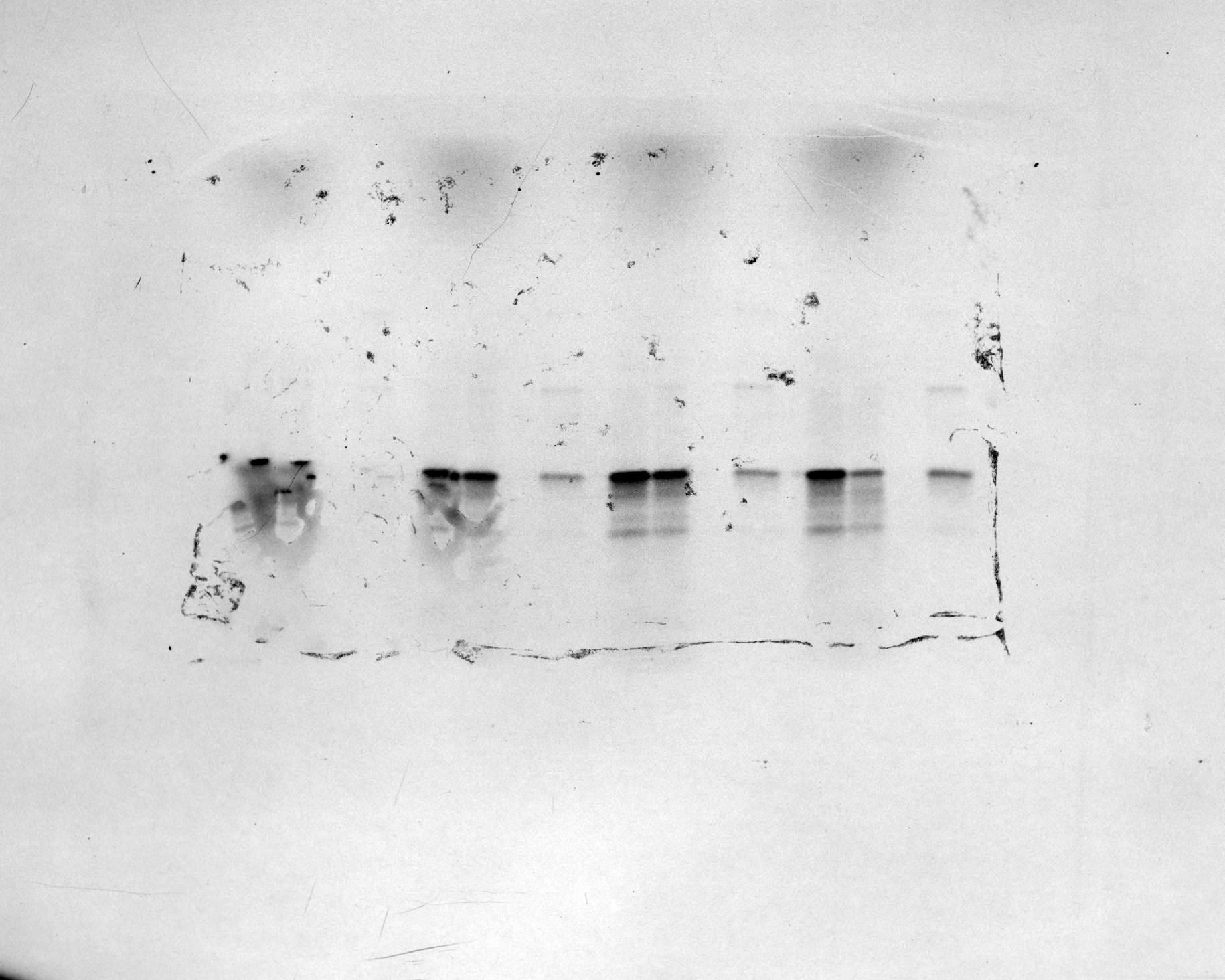

Supplement: Supplementary file 6 — Source data Fig. 5 [file 44318_2026_745_MOESM6_ESM.zip › Figure 5/5C/5C_PH3_M446R_Orginal.jpg]

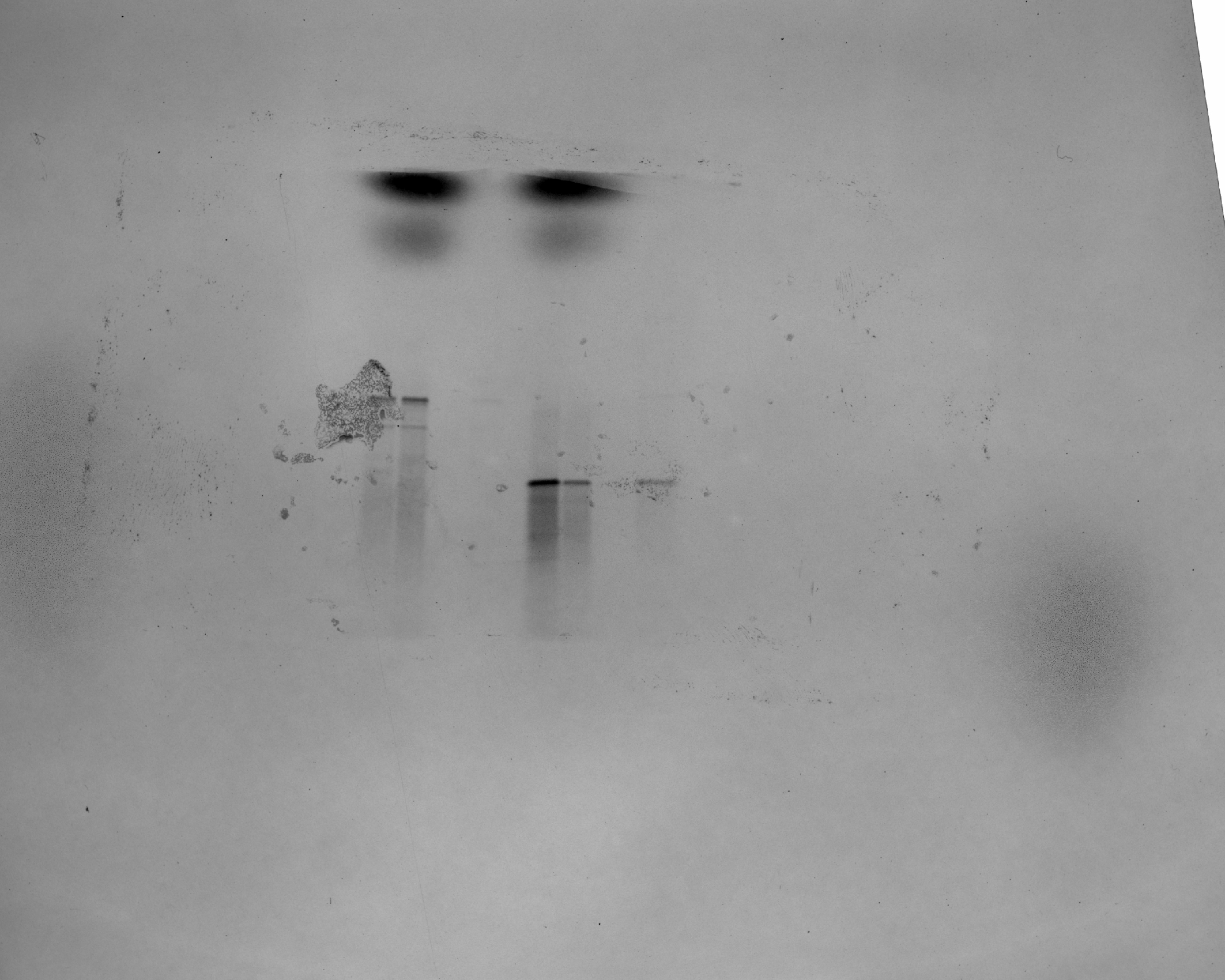

Supplement: Supplementary file 6 — Source data Fig. 5 [file 44318_2026_745_MOESM6_ESM.zip › Figure 5/5C/5C_PH3_P189T_Orginal.jpg]

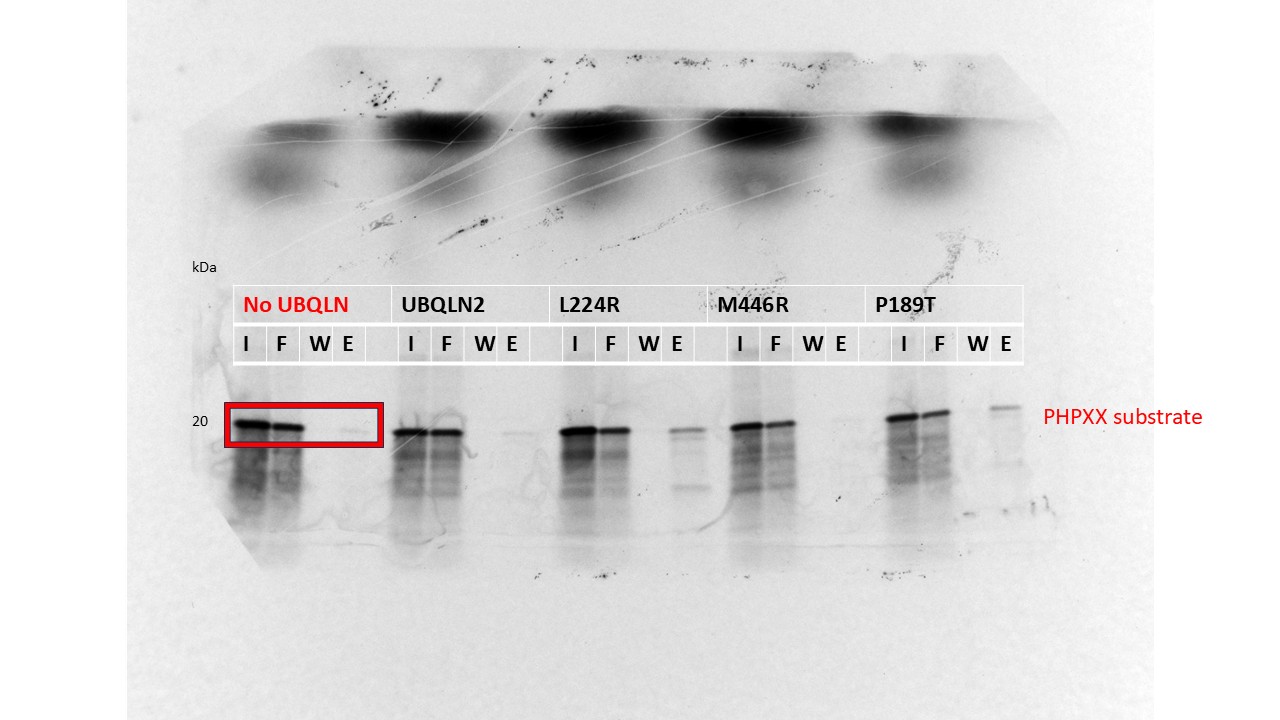

Supplement: Supplementary file 7 — Source data Fig. 6 [file 44318_2026_745_MOESM7_ESM.zip › Figure 6/6B/6B_NoUBQLN_annotated.jpg]

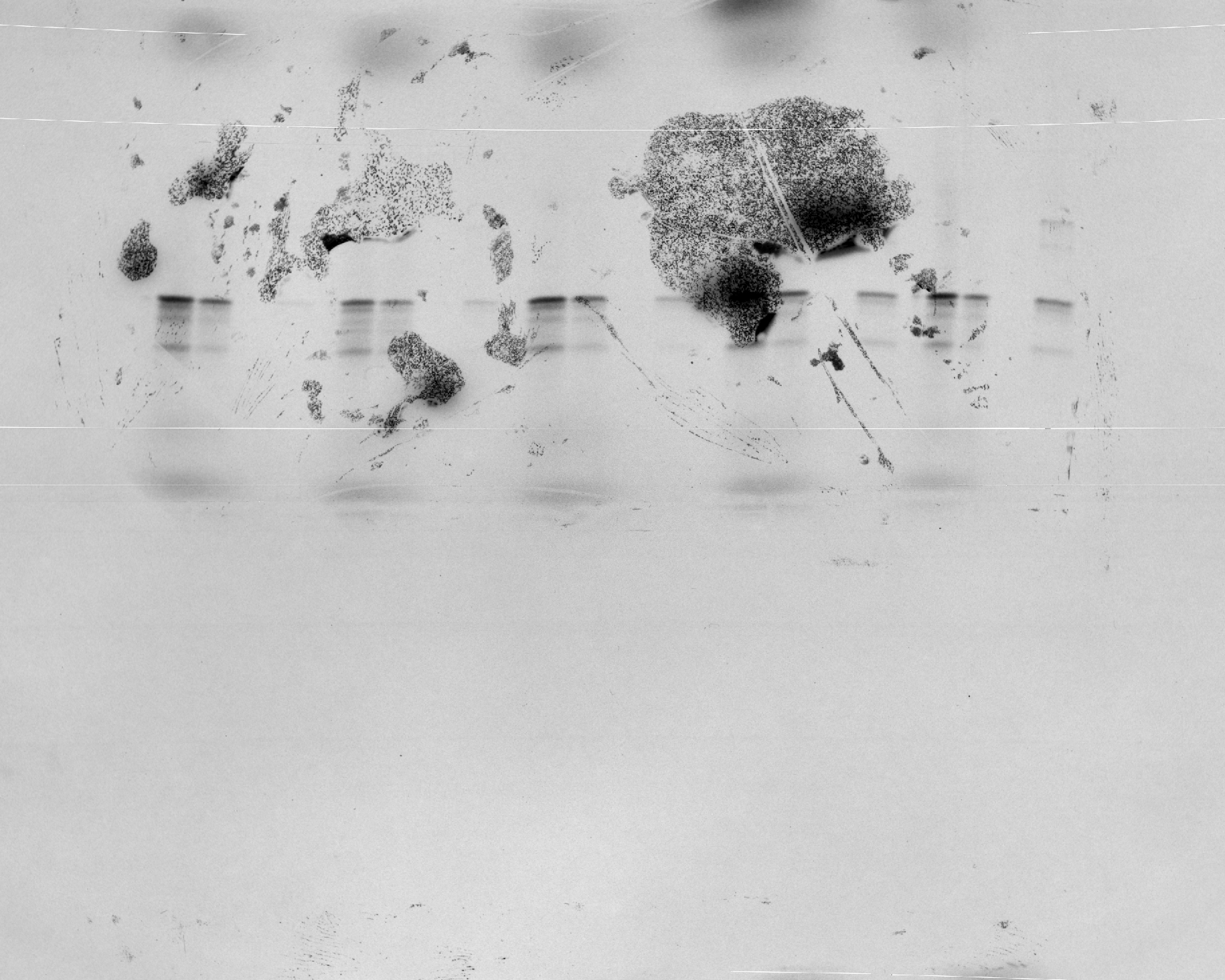

Supplement: Supplementary file 7 — Source data Fig. 6 [file 44318_2026_745_MOESM7_ESM.zip › Figure 6/6B/6B_WT_Orginal.jpg]

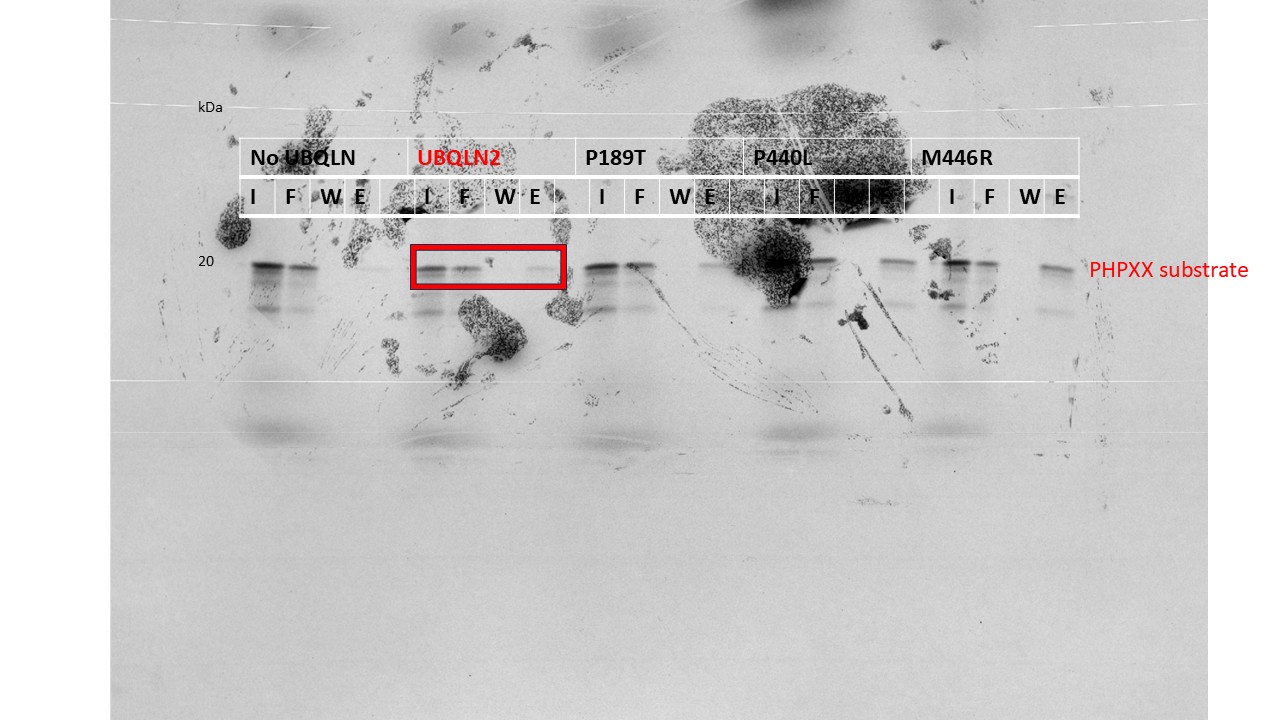

Supplement: Supplementary file 7 — Source data Fig. 6 [file 44318_2026_745_MOESM7_ESM.zip › Figure 6/6B/6B_WT_Annotated.jpg]

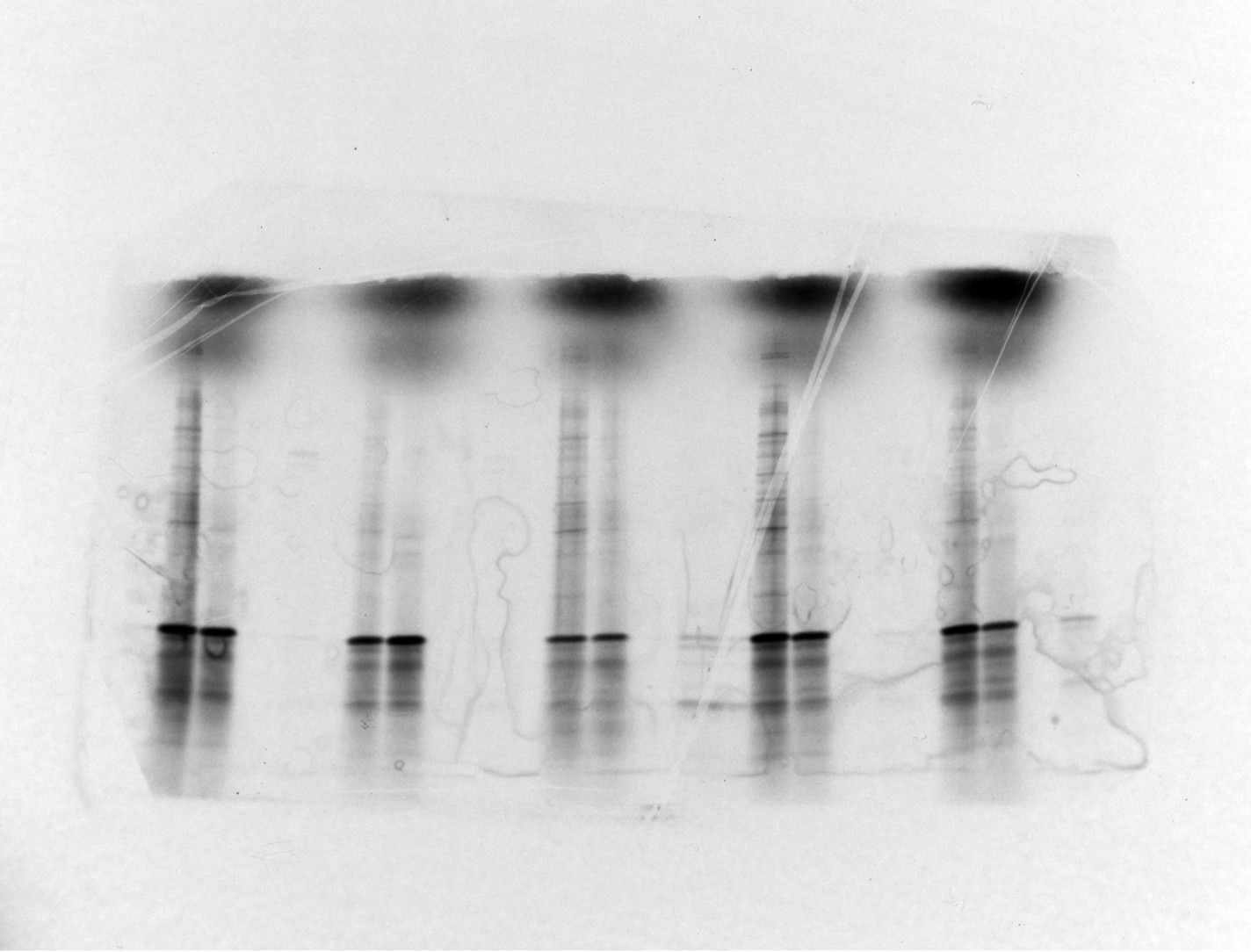

Supplement: Supplementary file 7 — Source data Fig. 6 [file 44318_2026_745_MOESM7_ESM.zip › Figure 6/6B/6B_P189T_P440L_Orginal.jpg]

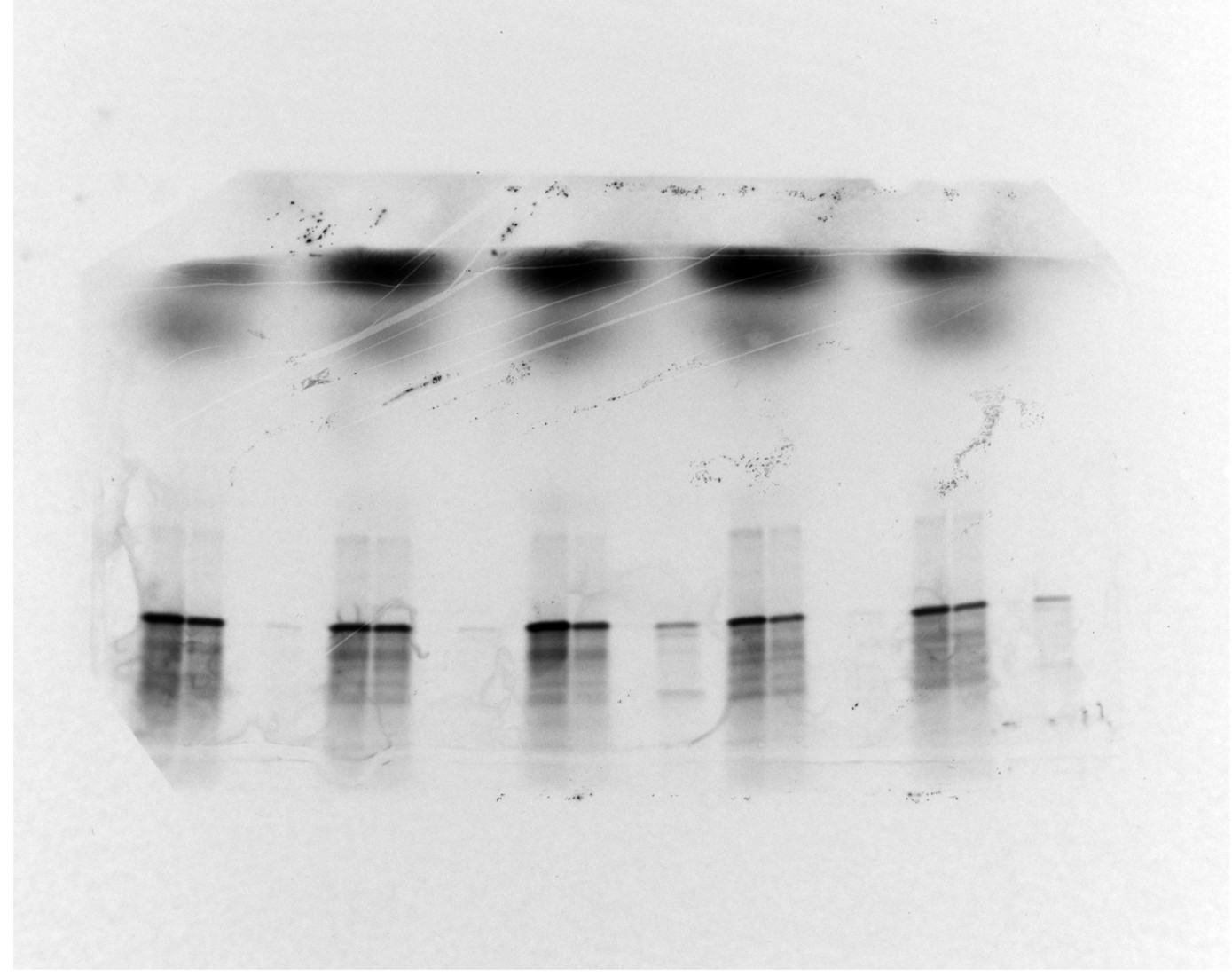

Supplement: Supplementary file 7 — Source data Fig. 6 [file 44318_2026_745_MOESM7_ESM.zip › Figure 6/6B/6B_NOUBQLN_Orinal.jpg]

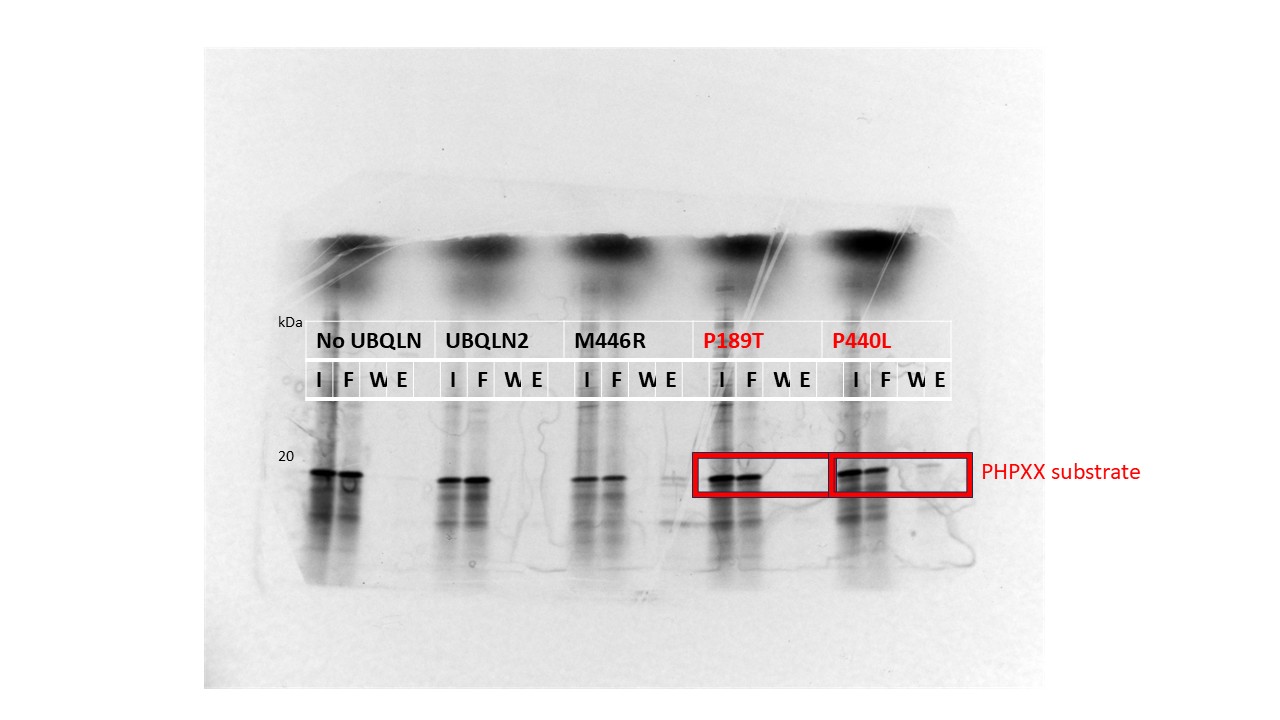

Supplement: Supplementary file 7 — Source data Fig. 6 [file 44318_2026_745_MOESM7_ESM.zip › Figure 6/6B/6B_P189T_P440L_annotated.jpg]

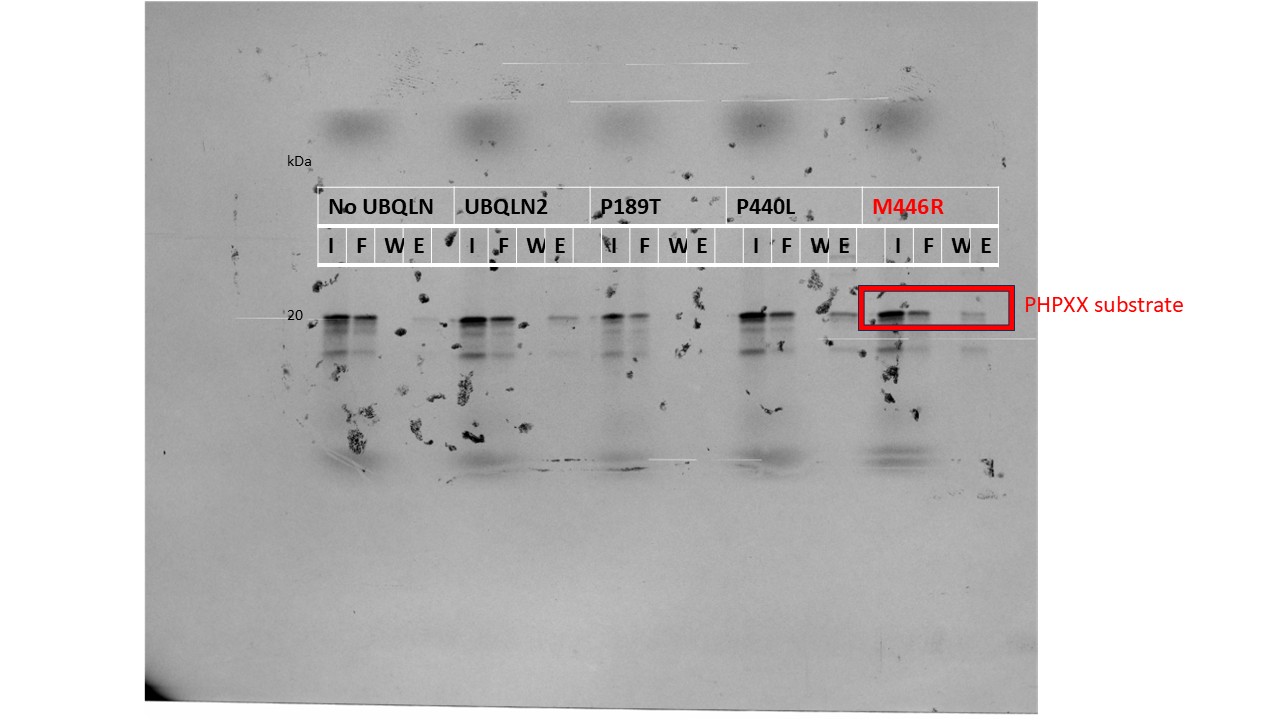

Supplement: Supplementary file 7 — Source data Fig. 6 [file 44318_2026_745_MOESM7_ESM.zip › Figure 6/6B/6B_M446R_annotated.jpg]

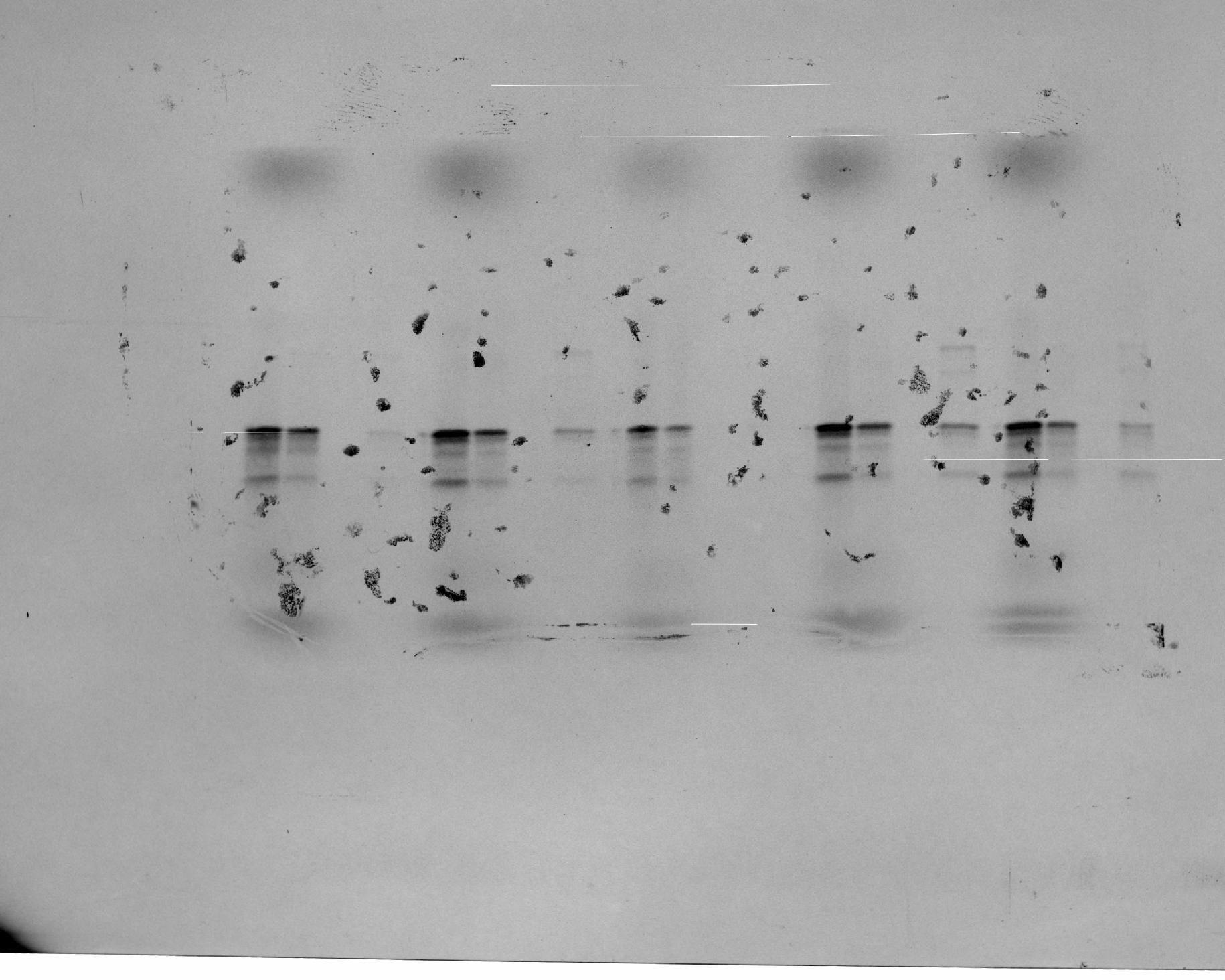

Supplement: Supplementary file 7 — Source data Fig. 6 [file 44318_2026_745_MOESM7_ESM.zip › Figure 6/6B/6B_M446R_Orginal.jpg]

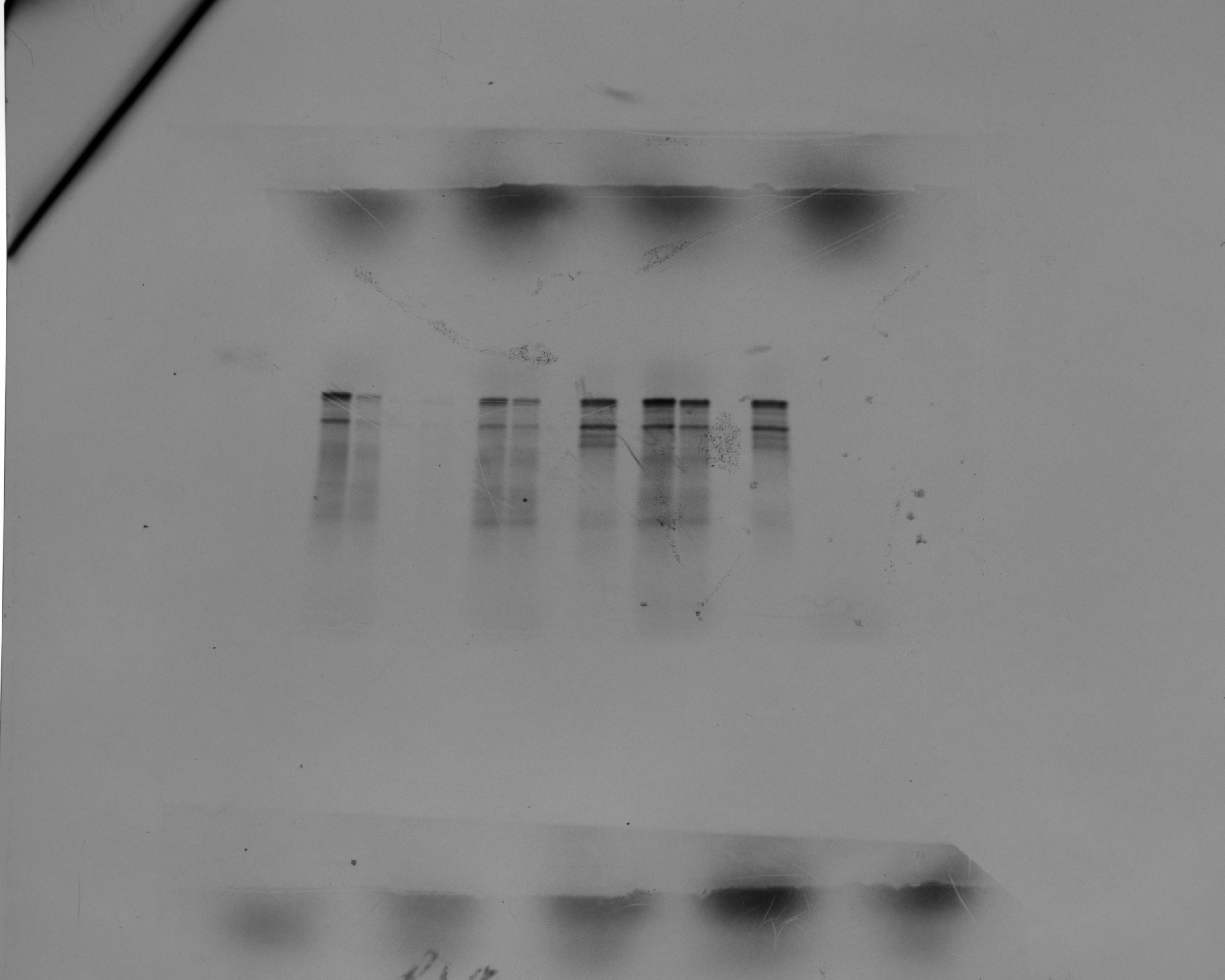

Supplement: Supplementary file 9 — Appendix Figure S4 Source Data [file 44318_2026_745_MOESM9_ESM.zip › Appendix Figure S4/S4C/S4C_Orginal.jpg]

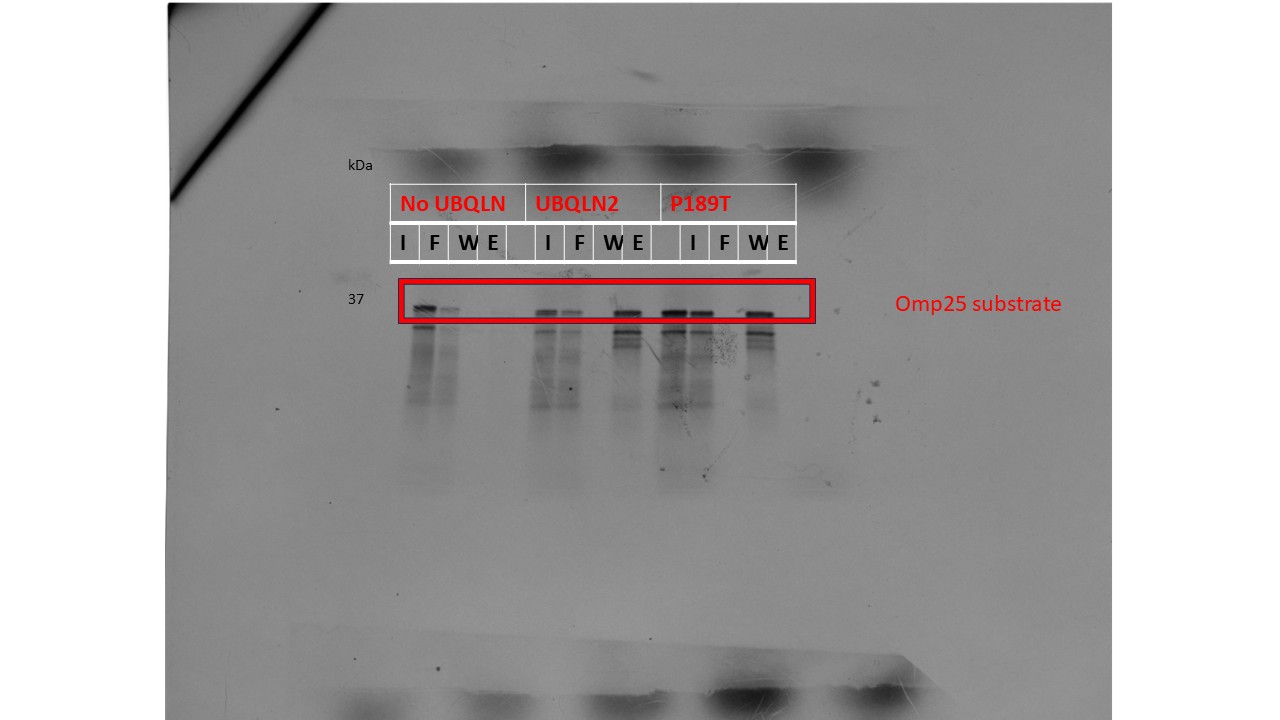

Supplement: Supplementary file 9 — Appendix Figure S4 Source Data [file 44318_2026_745_MOESM9_ESM.zip › Appendix Figure S4/S4C/S4C_Annotated.jpg]

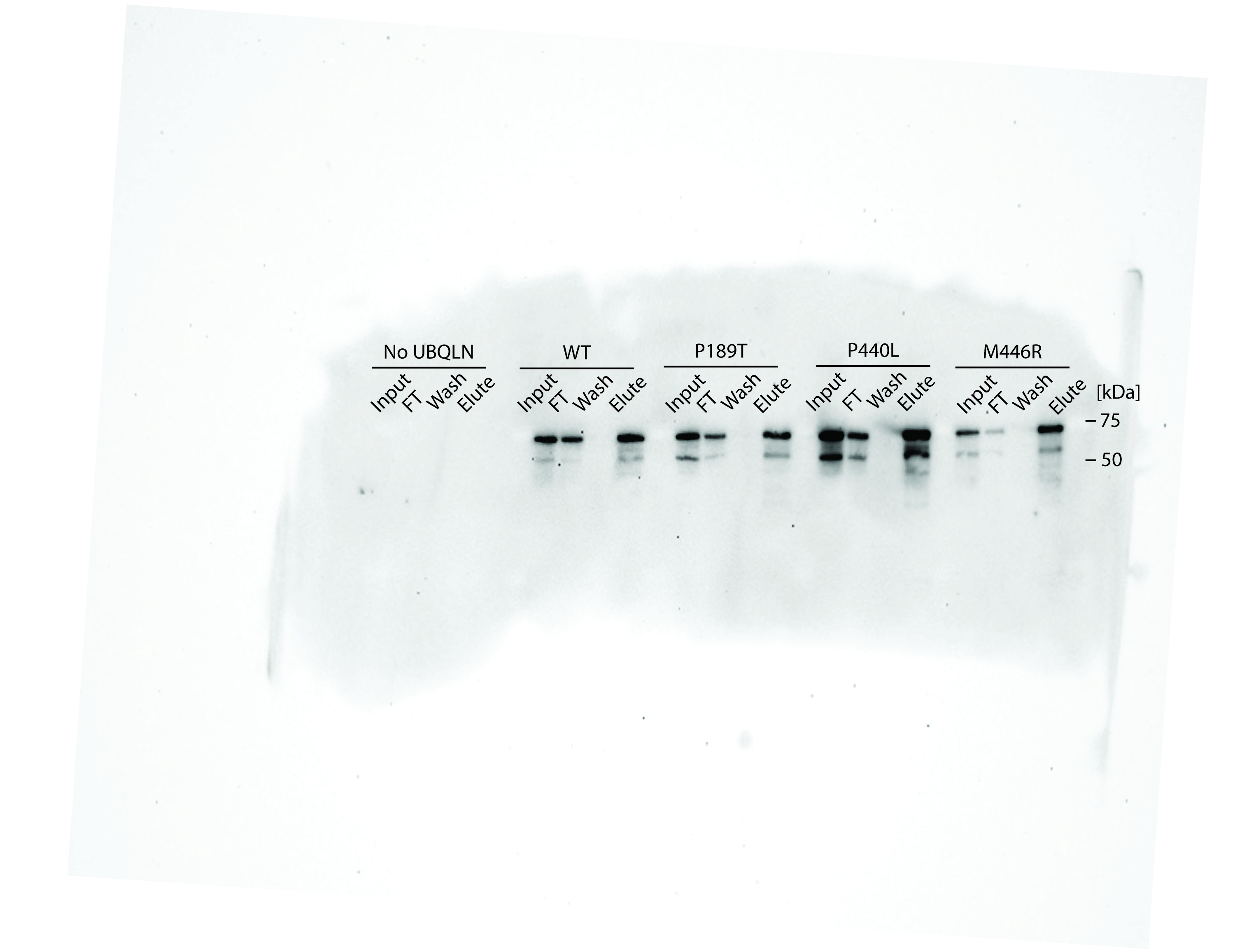

Supplement: Supplementary file 9 — Appendix Figure S4 Source Data [file 44318_2026_745_MOESM9_ESM.zip › Appendix Figure S4/S4B/S4B_annotated.tif]

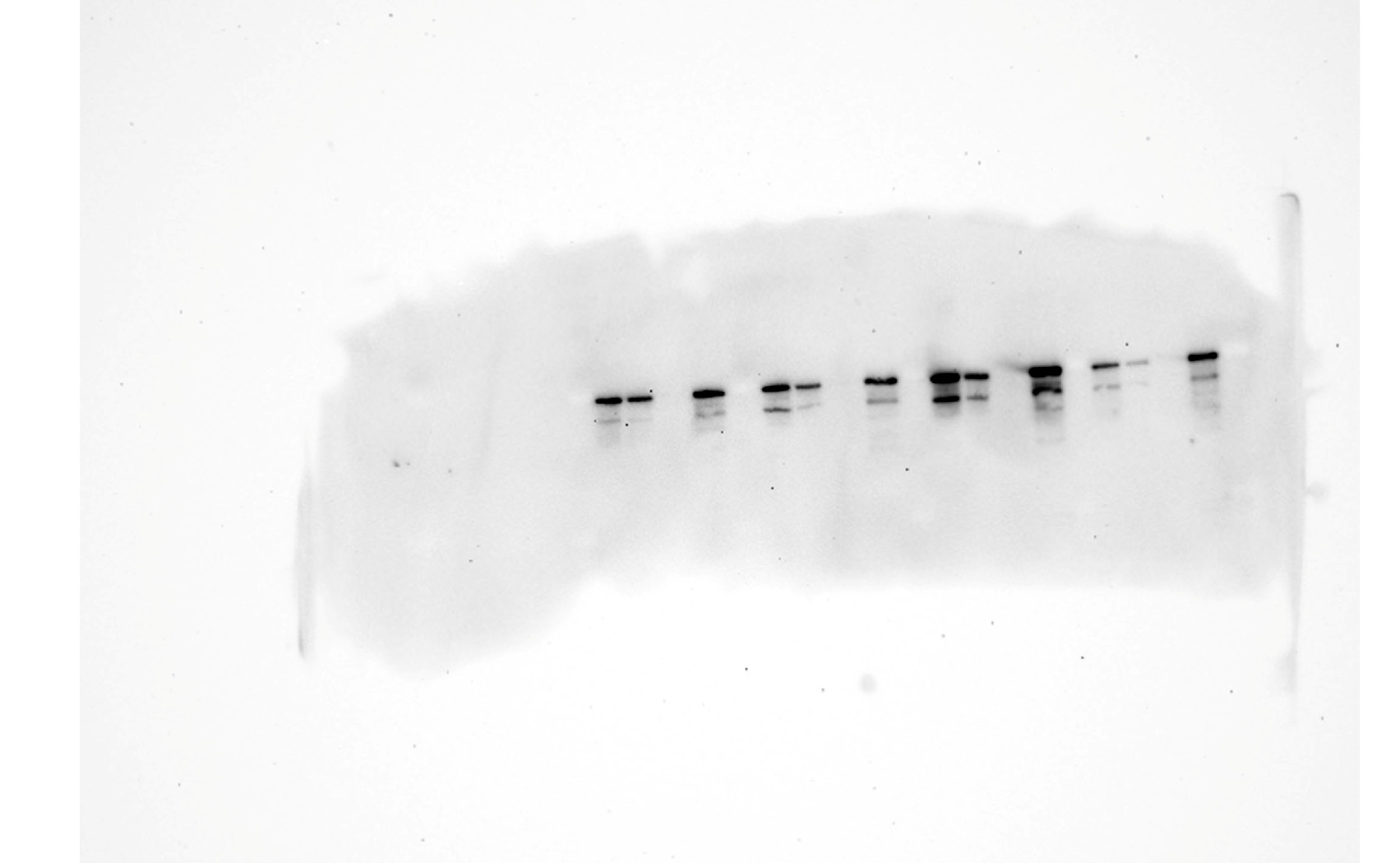

Supplement: Supplementary file 9 — Appendix Figure S4 Source Data [file 44318_2026_745_MOESM9_ESM.zip › Appendix Figure S4/S4B/S4B_original.jpg]

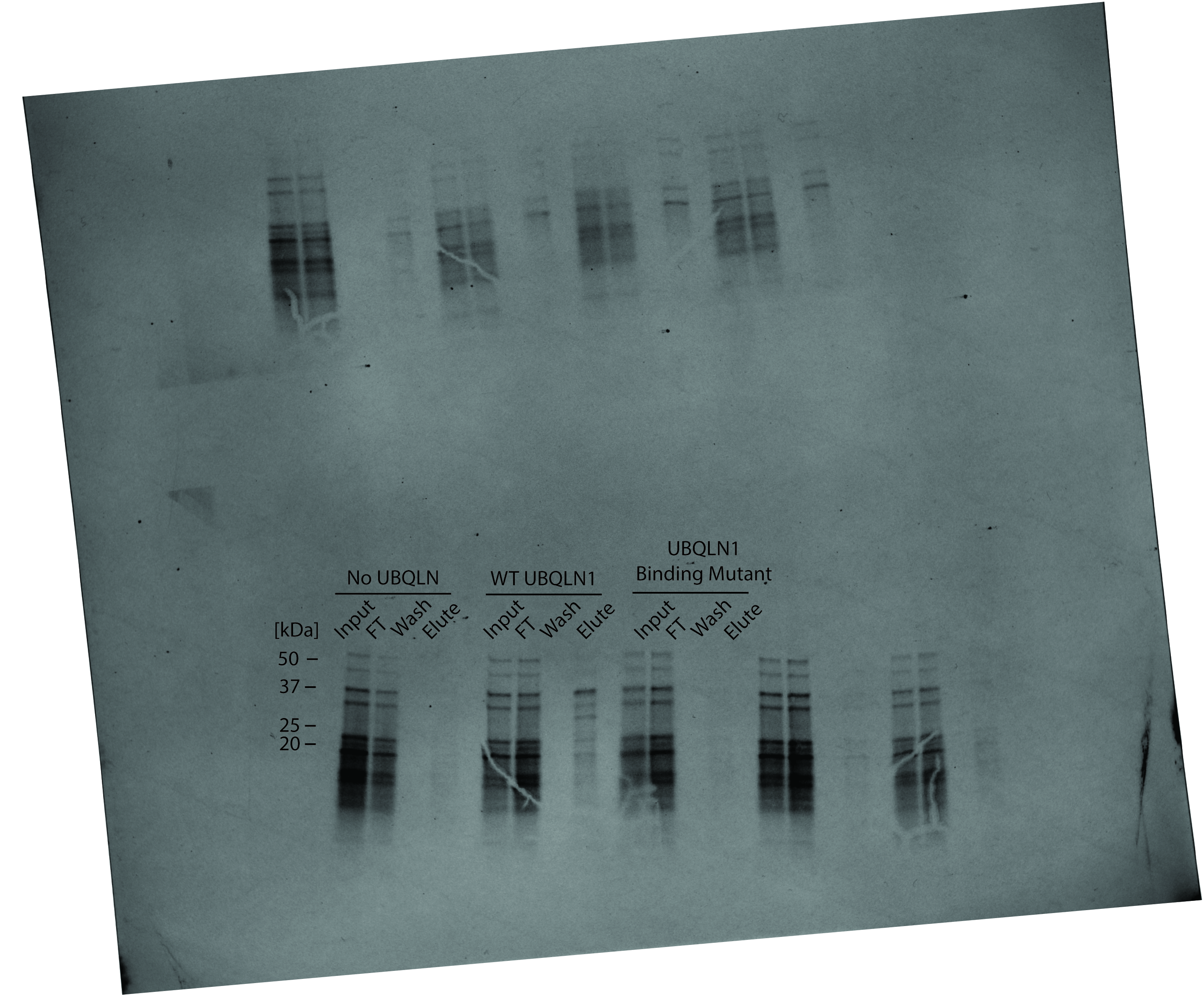

Supplement: Supplementary file 10 — Appendix Figure S5 Source Data [file 44318_2026_745_MOESM10_ESM.zip › Appendix Figure S5/S5A/S5A_annotated.tif]

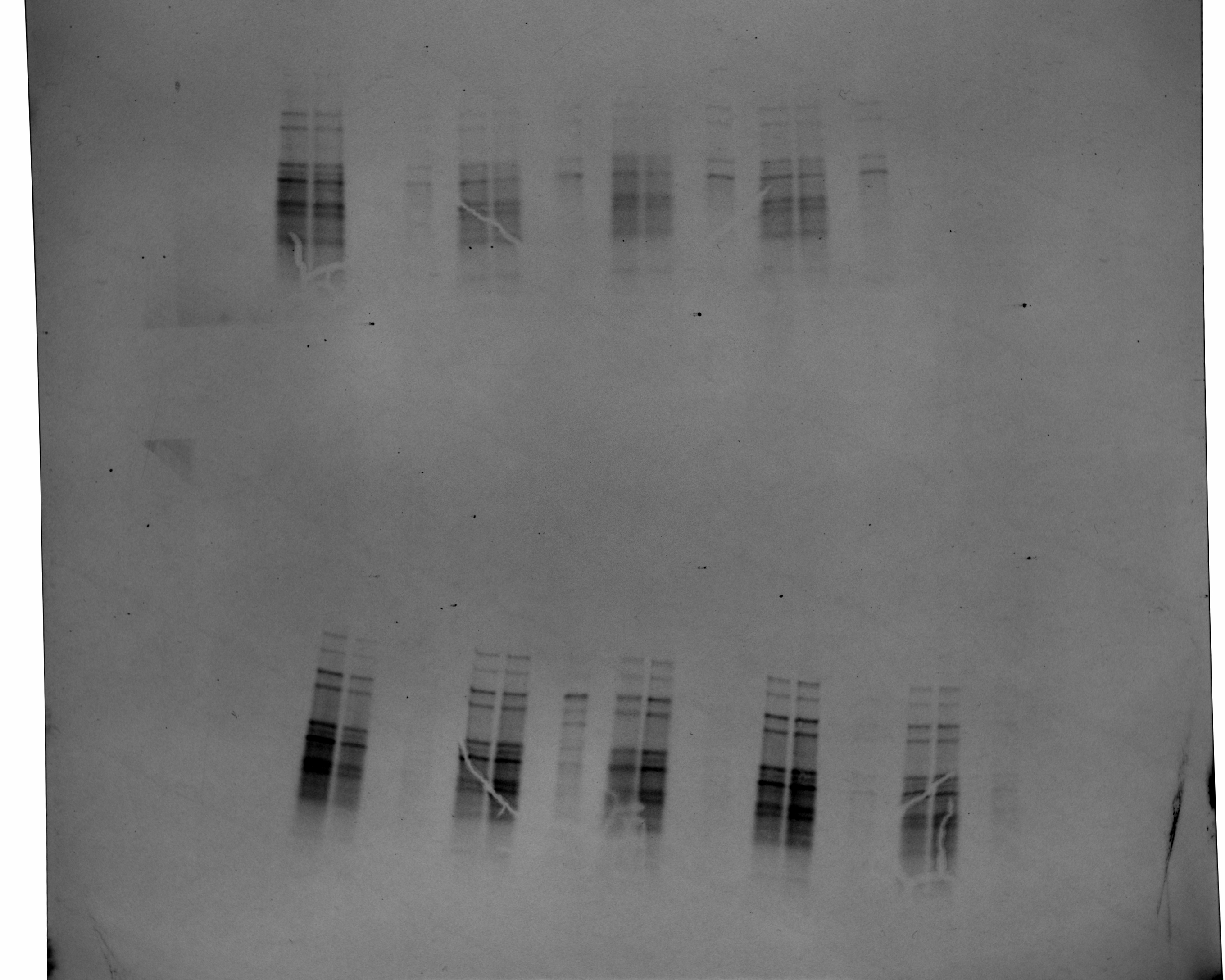

Supplement: Supplementary file 10 — Appendix Figure S5 Source Data [file 44318_2026_745_MOESM10_ESM.zip › Appendix Figure S5/S5A/S5A_original.jpg]

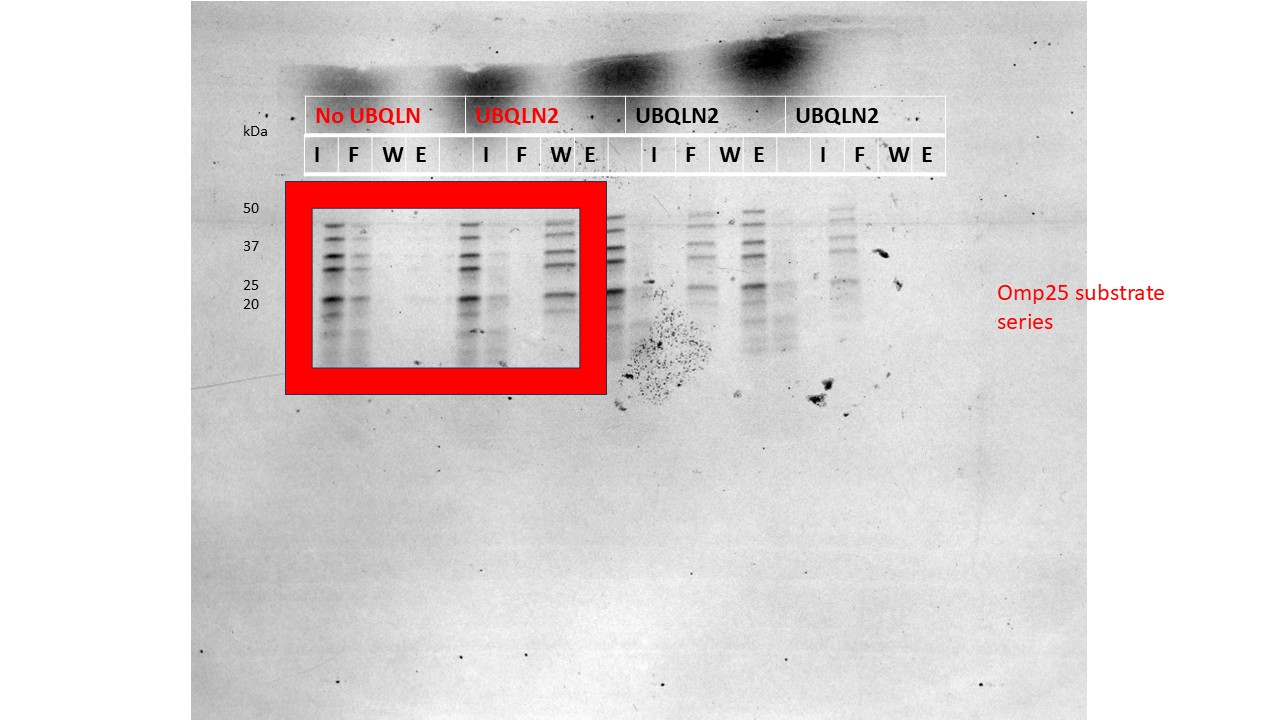

Supplement: Supplementary file 10 — Appendix Figure S5 Source Data [file 44318_2026_745_MOESM10_ESM.zip › Appendix Figure S5/S5B/S5B_Annotated.jpg]

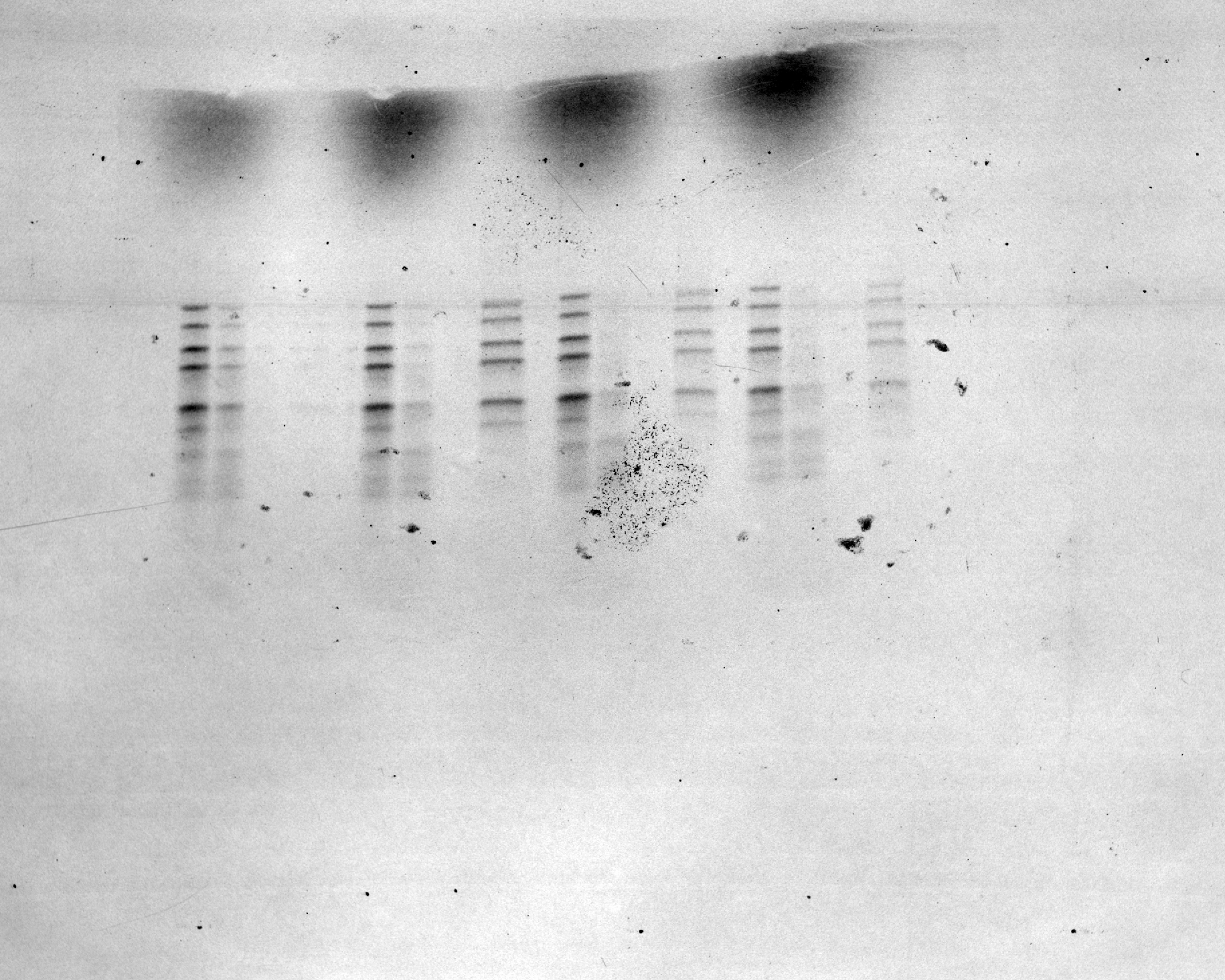

Supplement: Supplementary file 10 — Appendix Figure S5 Source Data [file 44318_2026_745_MOESM10_ESM.zip › Appendix Figure S5/S5B/S5B_Orginal.jpg]

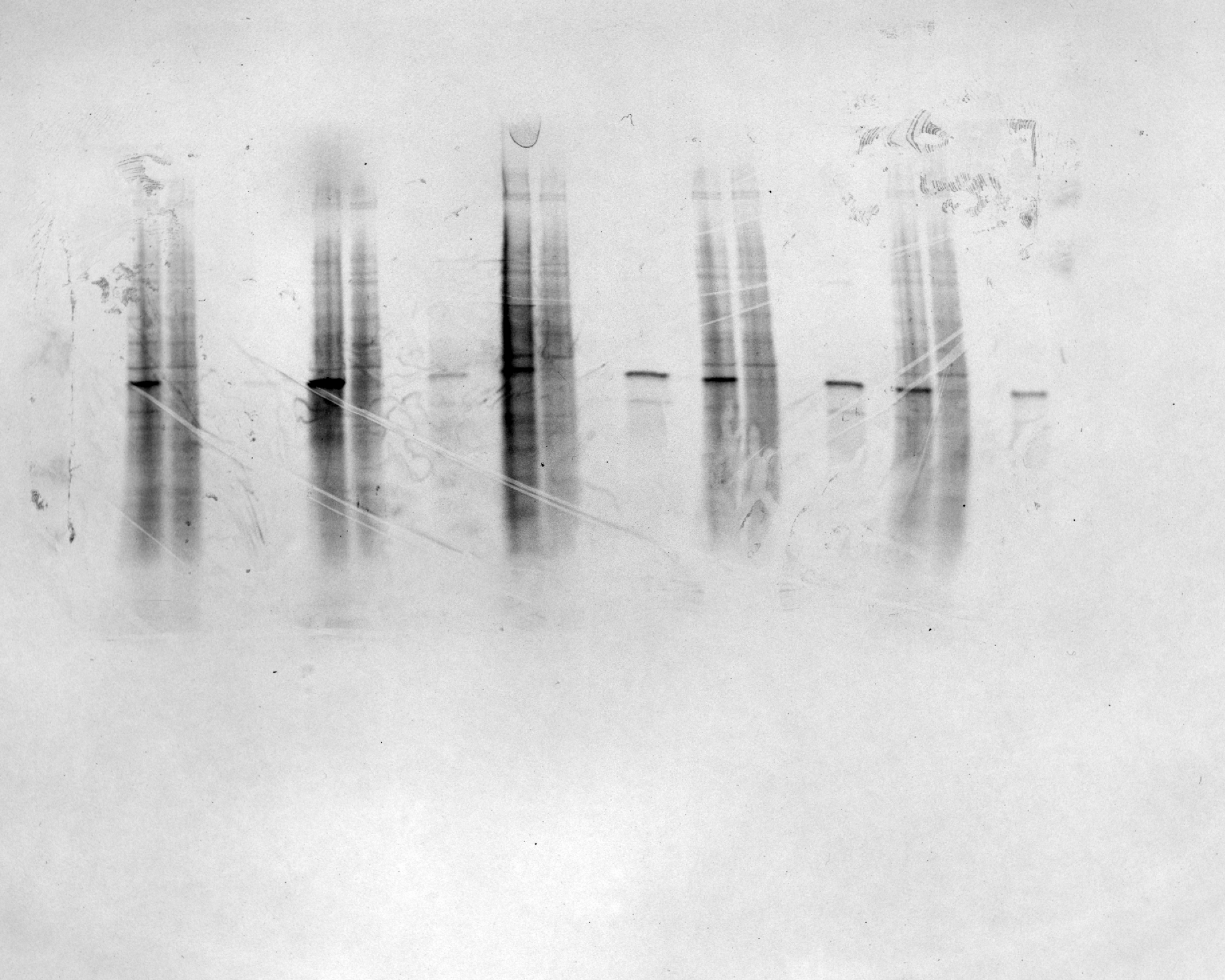

Supplement: Supplementary file 10 — Appendix Figure S5 Source Data [file 44318_2026_745_MOESM10_ESM.zip › Appendix Figure S5/S5D/S5D_Orginal.jpg]

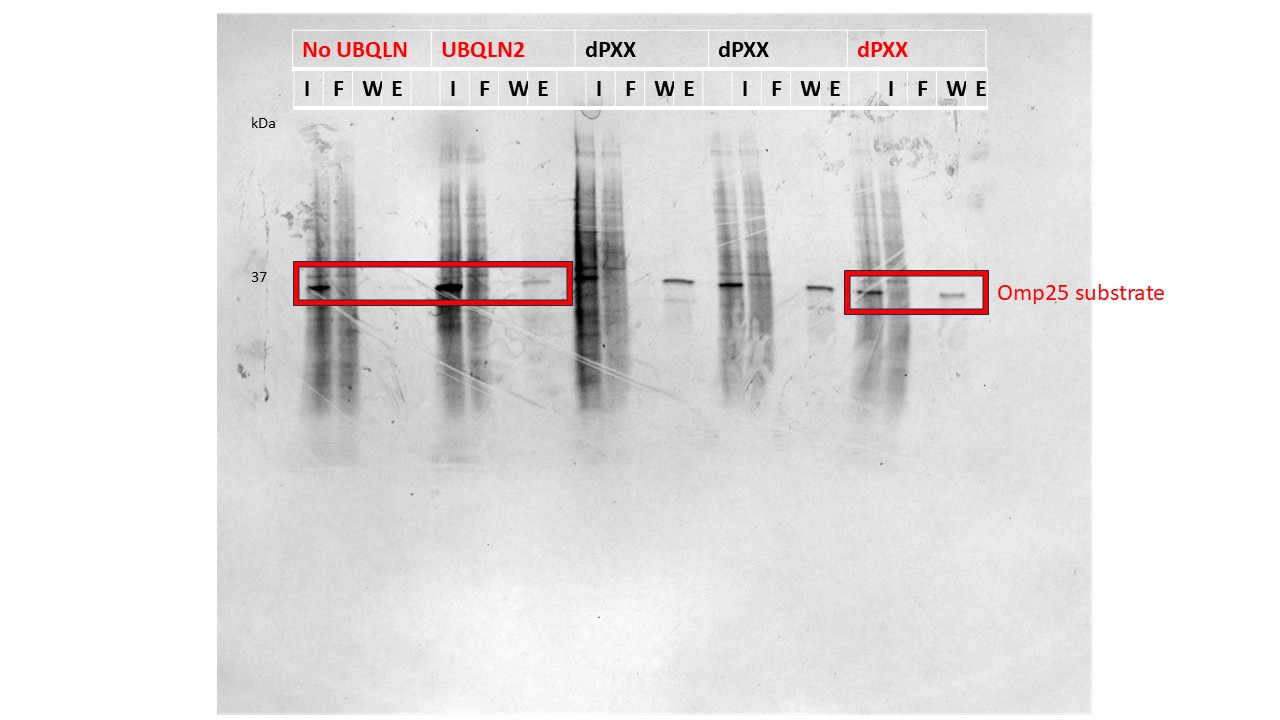

Supplement: Supplementary file 10 — Appendix Figure S5 Source Data [file 44318_2026_745_MOESM10_ESM.zip › Appendix Figure S5/S5D/S5D_Annotated.jpg]

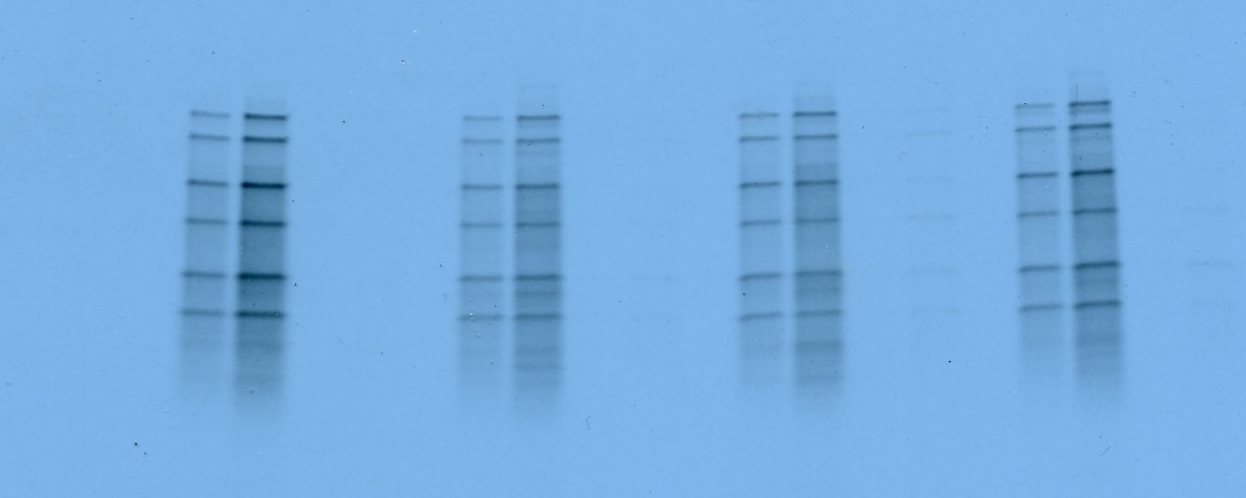

Supplement: Supplementary file 11 — Appendix Figure S6 Source Data [file 44318_2026_745_MOESM11_ESM.zip › Appendix Figure S6/S6A/S6A_film_original.jpeg]

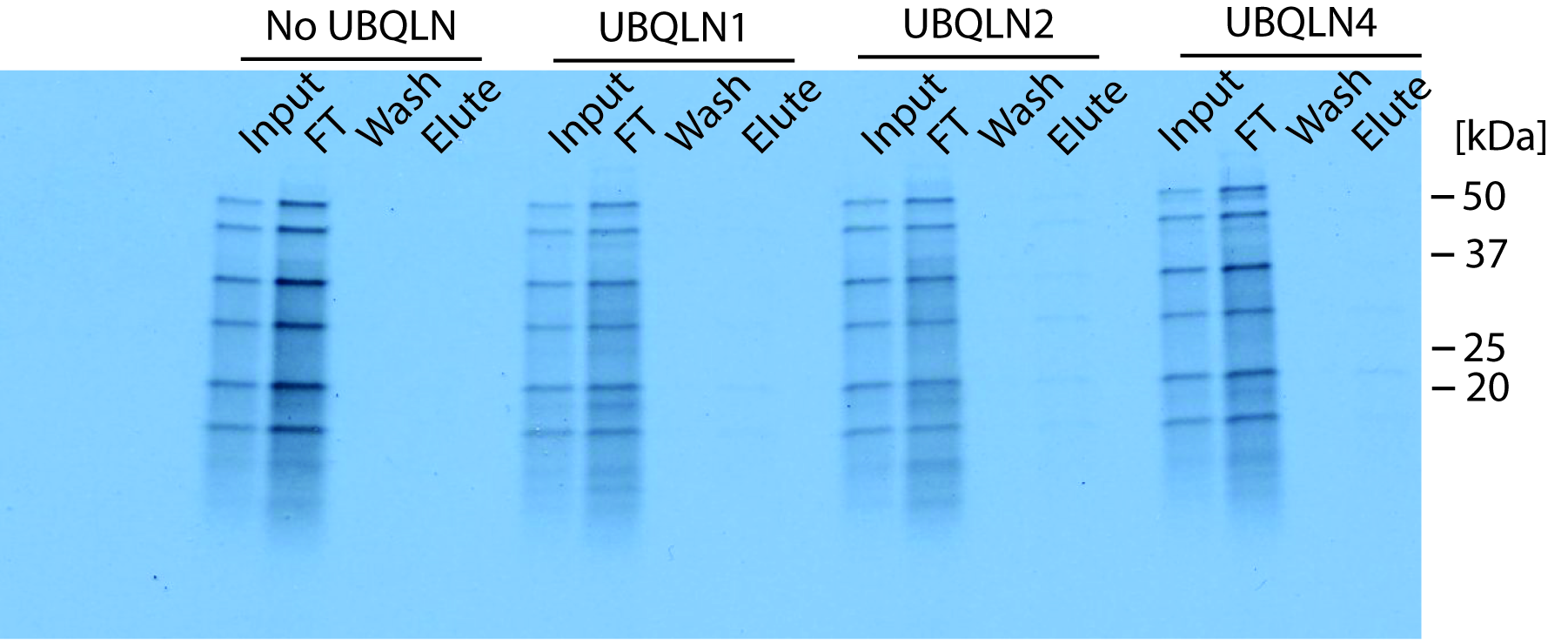

Supplement: Supplementary file 11 — Appendix Figure S6 Source Data [file 44318_2026_745_MOESM11_ESM.zip › Appendix Figure S6/S6A/S6A_film_annotated.tif]

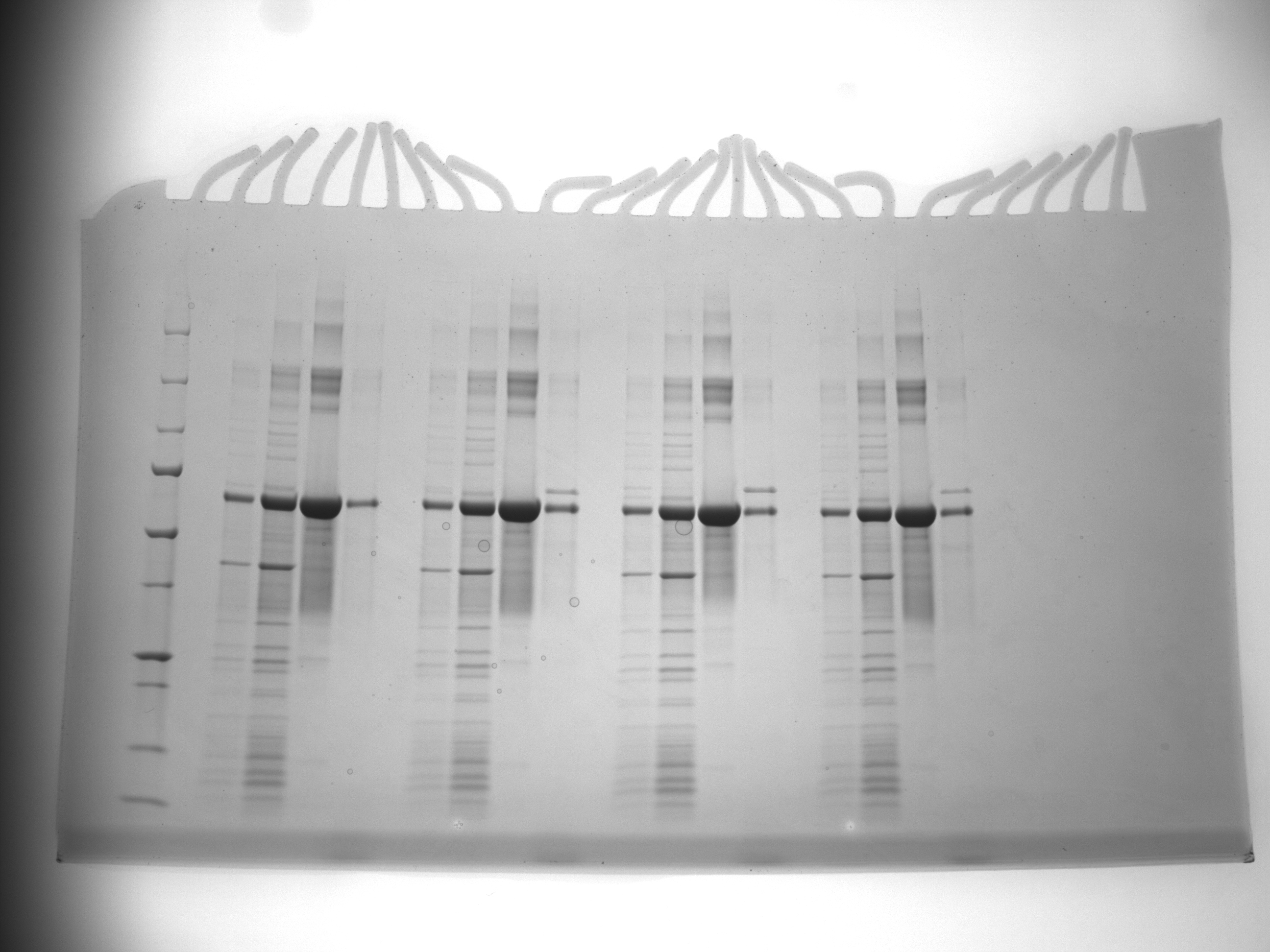

Supplement: Supplementary file 11 — Appendix Figure S6 Source Data [file 44318_2026_745_MOESM11_ESM.zip › Appendix Figure S6/S6A/S6A_gel_original.jpg]

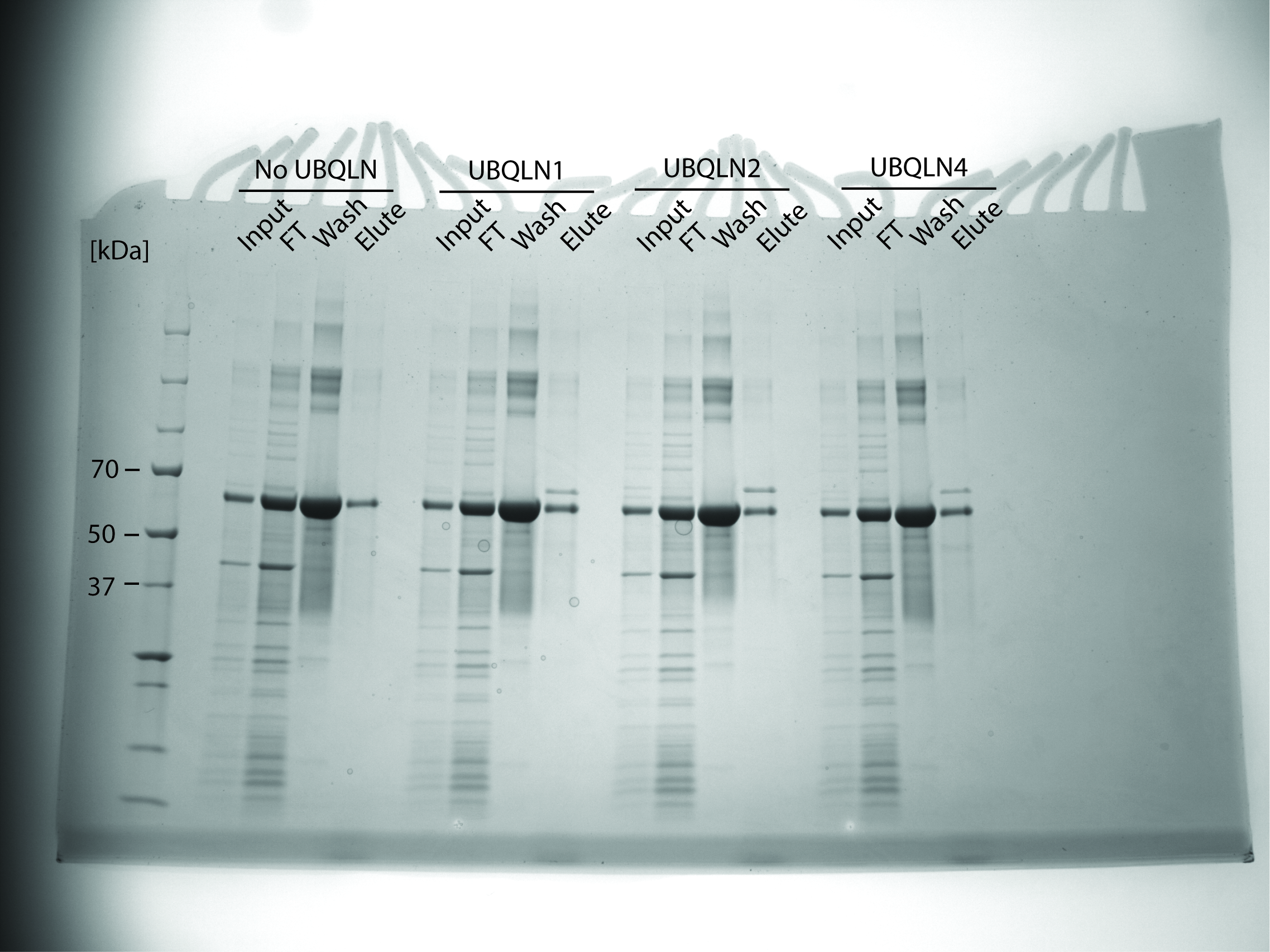

Supplement: Supplementary file 11 — Appendix Figure S6 Source Data [file 44318_2026_745_MOESM11_ESM.zip › Appendix Figure S6/S6A/S6A_gel_annotated.tif]

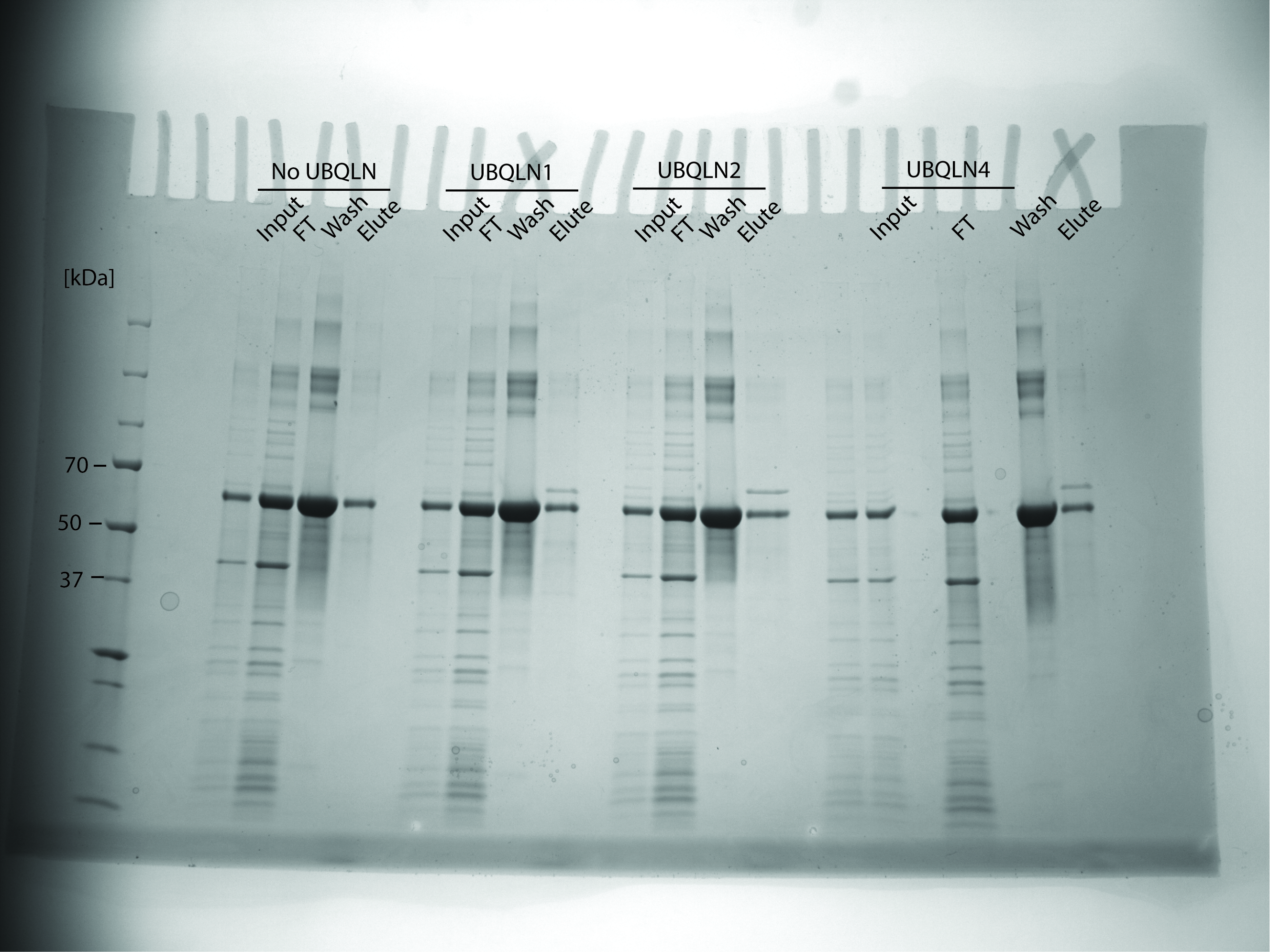

Supplement: Supplementary file 12 — Appendix Figure S7 Source Data [file 44318_2026_745_MOESM12_ESM.zip › Appendix Figure S7/S7A/S7A_gel_annotated.tif]

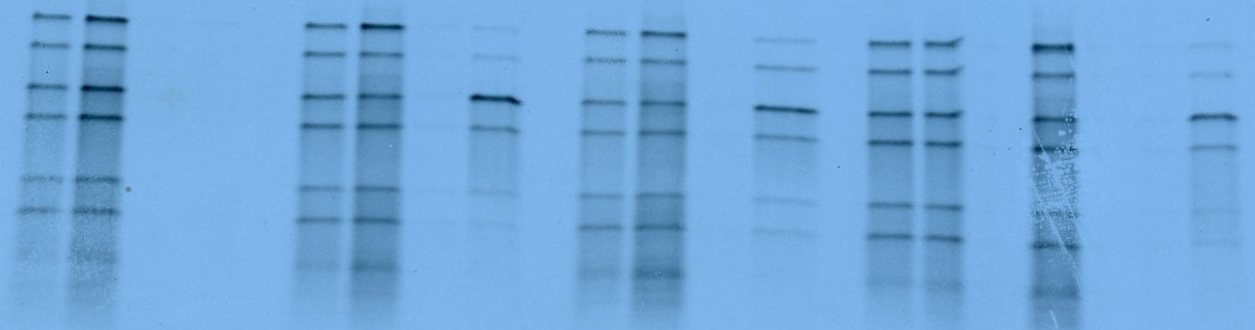

Supplement: Supplementary file 12 — Appendix Figure S7 Source Data [file 44318_2026_745_MOESM12_ESM.zip › Appendix Figure S7/S7A/S7A_film_original.jpg]

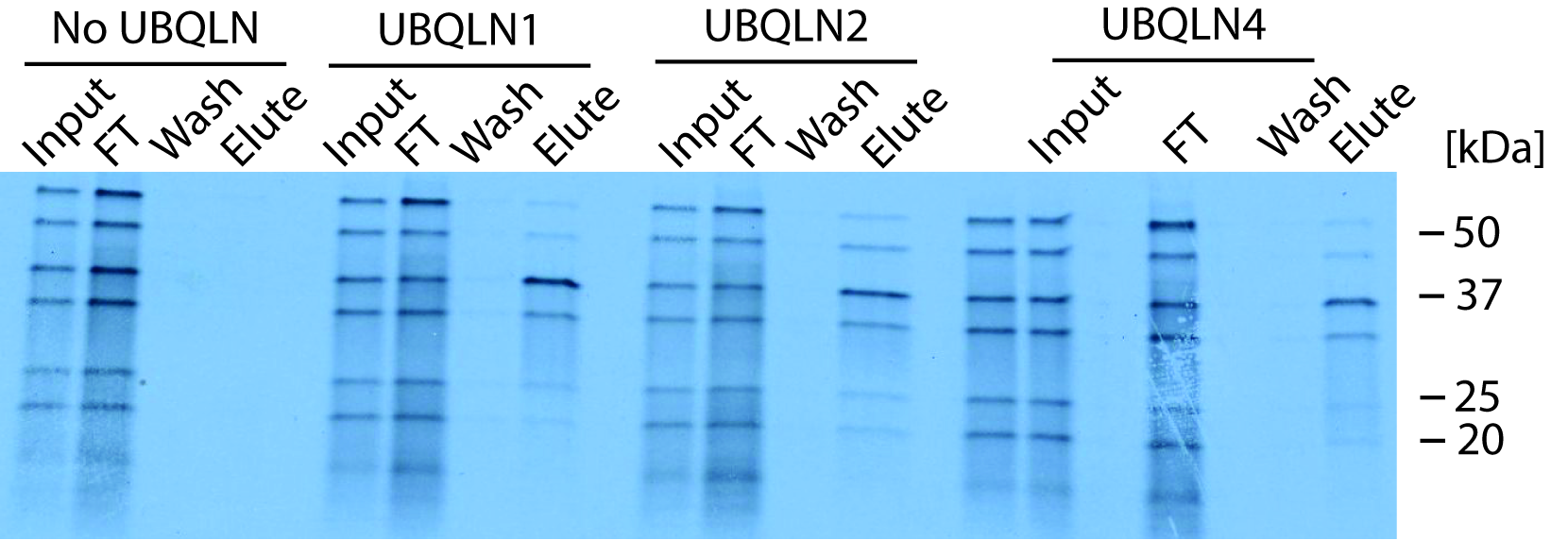

Supplement: Supplementary file 12 — Appendix Figure S7 Source Data [file 44318_2026_745_MOESM12_ESM.zip › Appendix Figure S7/S7A/S7A_film_annotated.tif]

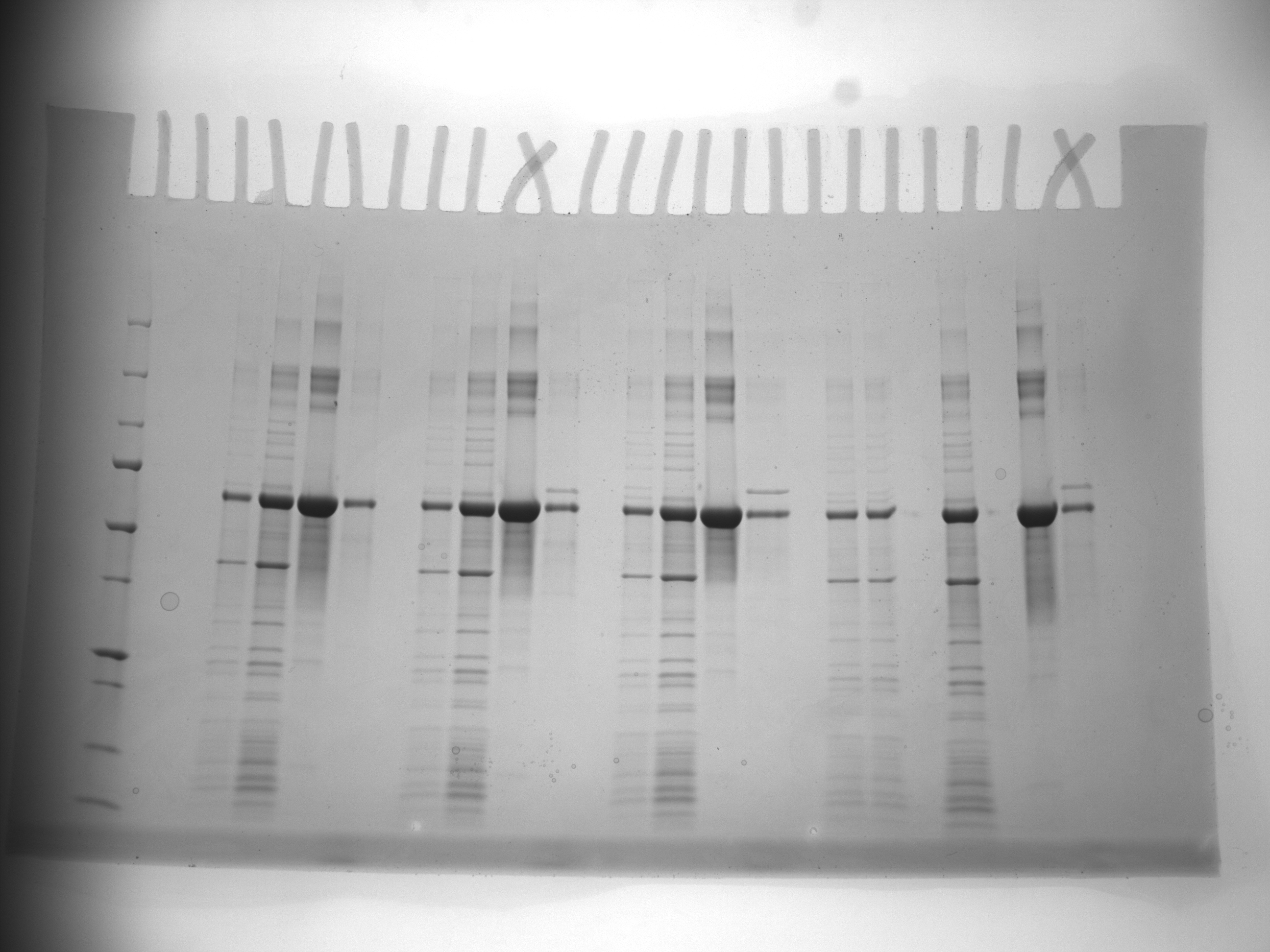

Supplement: Supplementary file 12 — Appendix Figure S7 Source Data [file 44318_2026_745_MOESM12_ESM.zip › Appendix Figure S7/S7A/S7A_gel_original.jpg]

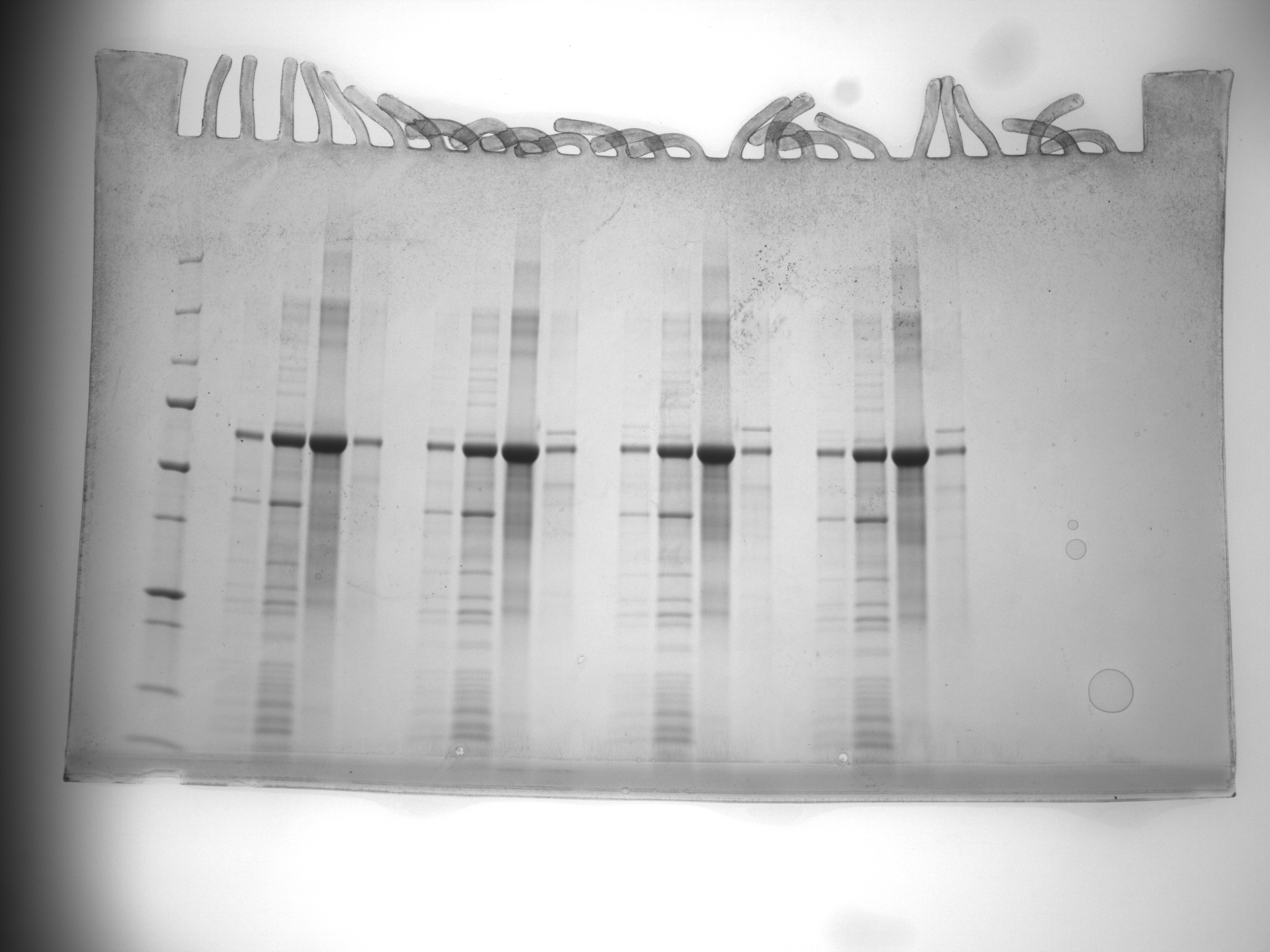

Supplement: Supplementary file 13 — Appendix Figure S8 Source Data [file 44318_2026_745_MOESM13_ESM.zip › Appendix Figure S8/S8A/S8A_gel_original.jpg]

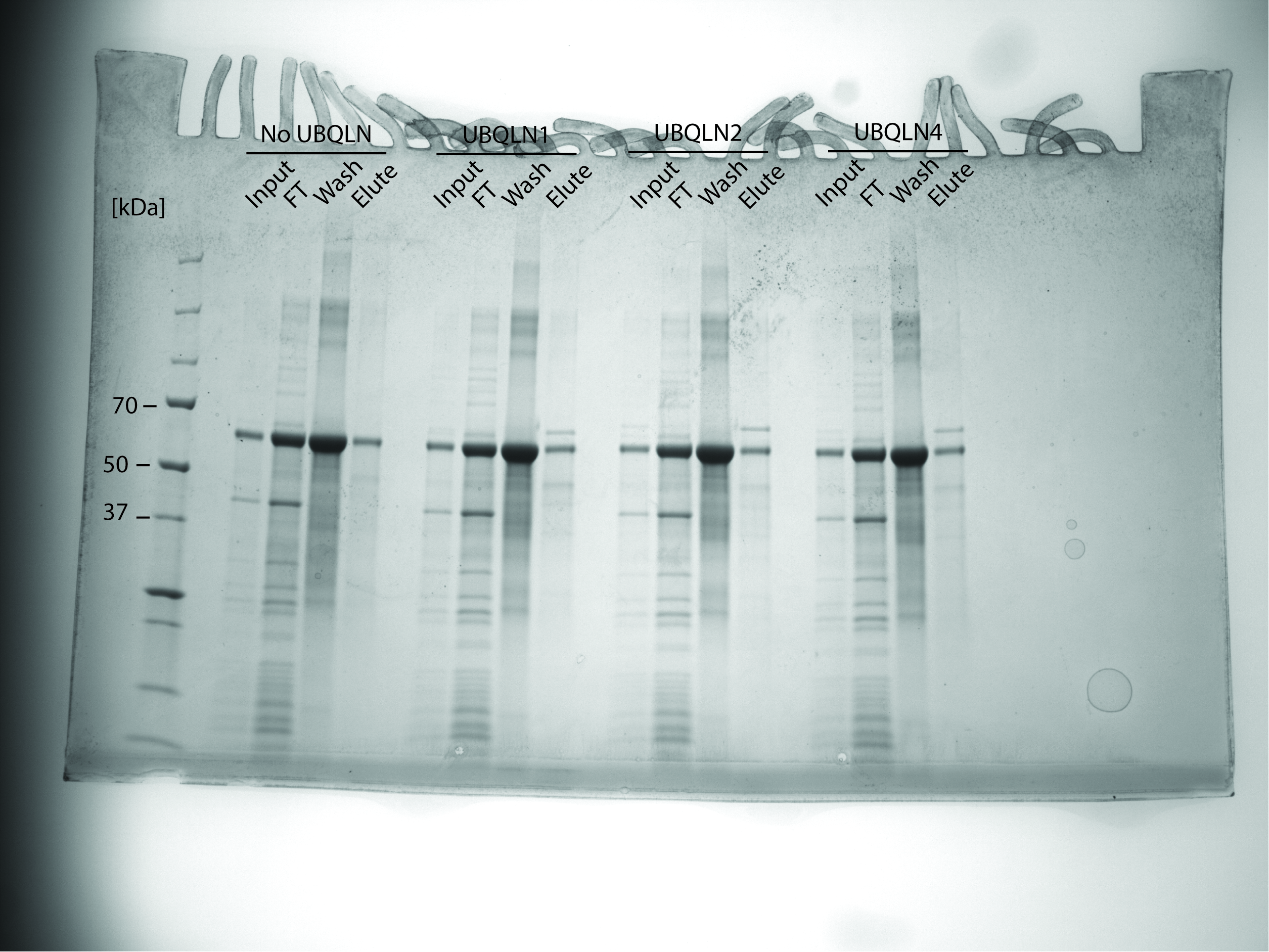

Supplement: Supplementary file 13 — Appendix Figure S8 Source Data [file 44318_2026_745_MOESM13_ESM.zip › Appendix Figure S8/S8A/S8A_gel_annotated.tif]

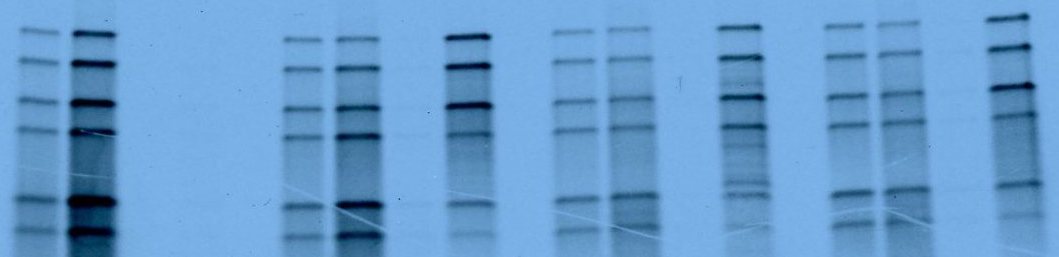

Supplement: Supplementary file 13 — Appendix Figure S8 Source Data [file 44318_2026_745_MOESM13_ESM.zip › Appendix Figure S8/S8A/S8A_film_original.jpg]

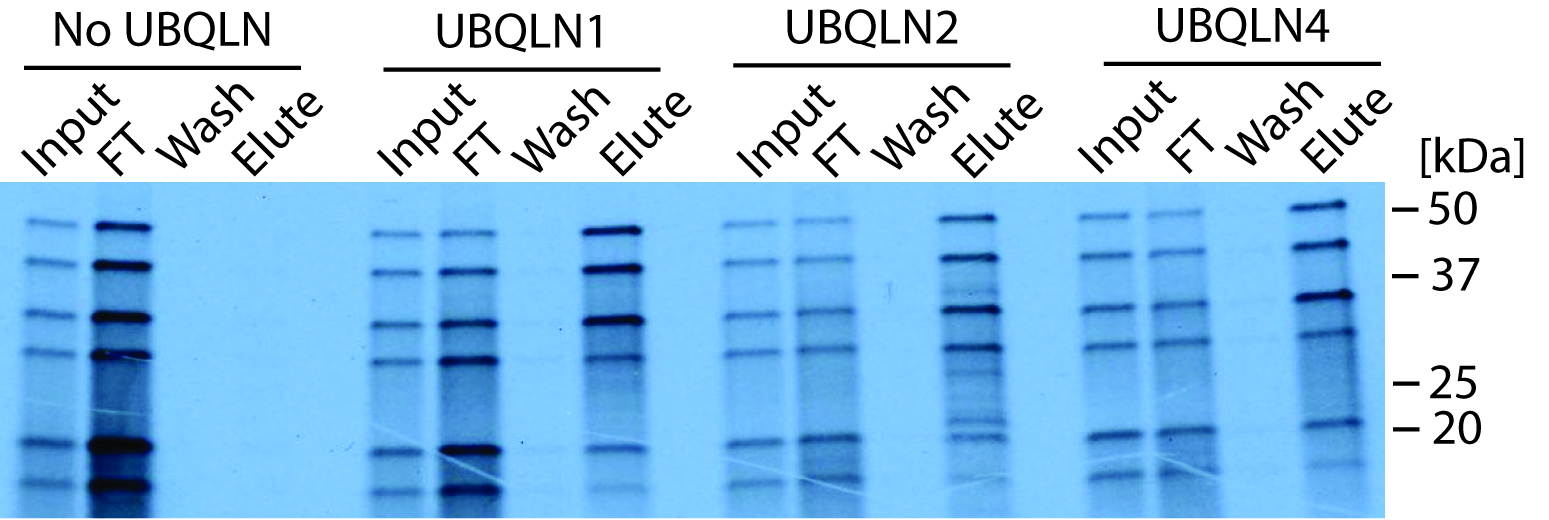

Supplement: Supplementary file 13 — Appendix Figure S8 Source Data [file 44318_2026_745_MOESM13_ESM.zip › Appendix Figure S8/S8A/S8A_film_annotated.tif]
